# Palladium-catalyzed incorporation of atmospheric CO_2_: efficient synthesis of functionalized oxazolidinones†
†Electronic supplementary information (ESI) available: Experimental procedures, characterization of new products and control experiments. CCDC 1439789, 1439720 and 1439788. For ESI and crystallographic data in CIF or other electronic format see DOI: 10.1039/c6sc00419a


**DOI:** 10.1039/c6sc00419a

**Published:** 2016-03-10

**Authors:** Patricia García-Domínguez, Lorenz Fehr, Giulia Rusconi, Cristina Nevado

**Affiliations:** a Department of Chemistry , University of Zürich , Winterthurerstrasse 180 , CH-8057 , Switzerland . Email: cristina.nevado@chem.uzh.ch

## Abstract

Methods to incorporate atmospheric CO_2_ into organic molecules are on demand. Here we present two Pd-catalyzed multicomponent reactions that provide functionalized oxazolidinones from propargylamines, aryl halides and CO_2_ as starting materials. These transformations, devoid of high CO_2_ pressures, represent a streamlined stereocontrolled synthesis of previously inaccessible versions of these useful heterocycles in an atom-economic manner, as up to four new single bonds are formed in a single synthetic operation.

## Abstract 2

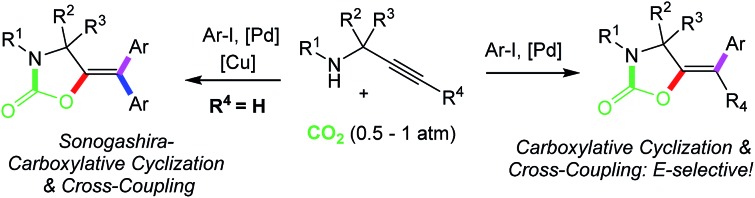
CO_2_ at atmospheric pressure is used in two Pd-catalyzed multicomponent reactions to produce functionalized 5-methylene-1,3-oxazolidin-2-ones from propargylamines and aryl halides.

## 

The large emissions of CO_2_ to the atmosphere represent an ever-growing problem^1^ that continues fostering the development of processes for capture and utilization (CCU)^2^ of this inexpensive and non-toxic C1 source towards valuable compounds within the chemical community.^3^ Multi-component reactions have emerged in recent years as powerful synthetic tools to assemble molecular complexity from either commercial or easily accessible starting materials.^4^ Among the value-added compounds that can be produced from CO_2_ activation and conversion, oxazolidinones are particularly appealing due to their broad application as chiral auxiliaries,^5^ intermediates in organic synthesis,^6^ as well as agrochemicals and antibacterial drugs.^7^ Strategies towards this attractive chemical blueprint employing simple starting materials combined with environmentally friendly CO_2_ as a C1 source are thus highly desirable. Several reports dealing with CO_2_ fixation by propargylamines or aminoalcohols to access these useful heterocycles have appeared during the last decade.^8^ Nevertheless, most of these methodologies involve harsh conditions including the use of supercritical CO_2_ (scCO_2_)^9^ or strong organic bases (super bases) at high temperatures and pressures of the gas.^10^ Very recently, milder protocols with protic ionic liquids and low CO_2_ pressures have been reported.^11^ π-Acid catalysts have also been employed for this purpose,^12^,^13^ with prevalence of coinage metals, as demonstrated by the seminal reports of Yamada and coworkers using silver^14^ and Ikariya's work employing gold(i) complexes.^15^ However, several limitations still hamper the broad synthetic applicability of these methods. The fact that C–C bond formation reactions have been scarcely coupled to the carboxylation of propargylamines prevented the formation of tetrasubstituted olefins,^16^ whereas commonly obtained trisubstituted alkenes could only be accessed in *Z*-selective fashion. Furthermore, due to CO_2_'s high thermodynamic and kinetic stability, substantial activation (temperature, pressure, strong agents) is still needed in most cases (Scheme 1, top). Inspired by the recognized flexibility of palladium catalysts, we have designed a one pot, 3-component carboxylation–cyclization–cross-coupling reaction to produce functionalized oxazolidinones out of simple building blocks (CO_2_, propargylamines and aryl halides) under mild reaction conditions. Up to four new bonds can be formed in a single operation during the reaction of terminal propargylamines through an additional Sonogashira cross-coupling reaction in the presence of copper salts. Our protocol, utilizing CO_2_ at atmospheric pressure furnishes, for the first time, tetrasubstituted 5-methylene-1,3-oxazolidin-2-ones in a stereocontrolled fashion. Moreover, the methodology described herein also allows the *E*-selective preparation of trisubstituted derivatives as well as modifications at the allylic position or at the N atom, thus encompassing different substitution patterns commonly found in oxazolidinone products (Scheme 1, bottom).

**Scheme 1:**
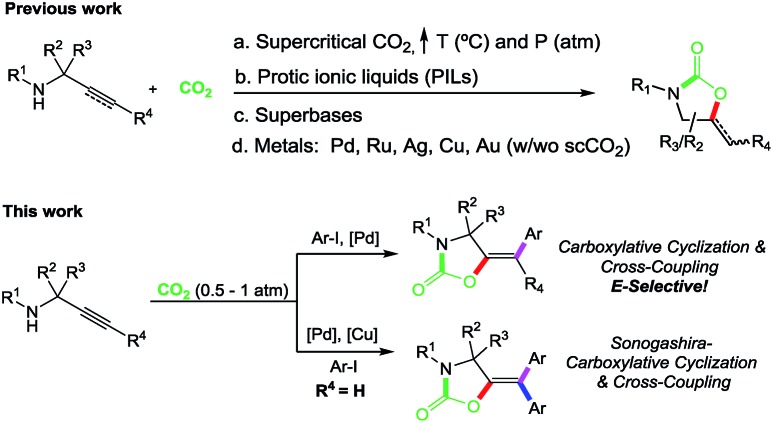
Synthesis of oxazolidinones from propargylamines.

Propargylamine **1** and iodobenzene (**2a**) were chosen as starting materials for the optimization of the reaction conditions. After a short preliminary screening,^17^ [PdCl_2_(dppf)] and NaO*t*Bu were identified as suitable catalyst and base respectively for the fine tuning of the reaction conditions. With a sustained atmospheric pressure of CO_2_ at 40 °C, different solvents were tested. The reaction with THF produced the desired product **3a** in 34% yield after 22 hours (Table 1, entry 1). A longer reaction time resulted in a limited increase of product yield (Table 1, entry 2). Different solvents were also screened (Table 1, entries 3–4) out of which DMSO was selected for the subsequent optimization as it delivered **3a** in 89% yield (Table 1, entry 5). Decreasing the amount of base turned out to be beneficial (Table 1, entries 6–7). In contrast, a decrease in the reaction time or temperature resulted in lower yields (Table 1, entries 8–9). A compromise could be found when 1.5 equiv. of base were used, resulting in a 95% yield of **3a** after 22 hours (Table 1, entry 10). Remarkably, no trace of 6-*endo*-dig cyclization products could be detected in these transformations.

**Table 1:** Optimization of the reaction conditions for carboxylative cyclization and cross-coupling reaction of propargylamine **1** and iodobenzene **2a**^*a*^

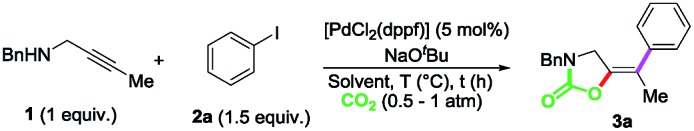					
Entry	Solvent	NaO*t*Bu (equiv.)	*T* [°C]	*t* [h]	Yield^*b*^ [%]
1	THF	3	40	22	34
2	THF	3	40	44	47
3	DMF	3	40	44	57
4	DCE	3	40	44	Traces^*c*^
5	DMSO	3	40	44	89
6	DMSO	1.5	40	44	94
7	DMSO	1.1	40	44	97
8	DMSO	1.1	40	22	87
9	DMSO	1.1	r.t.	22	72
**10**	**DMSO**	**1.5**	**40**	**22**	**95**

^*a*^The reactions were performed in a Schlenk tube directly connected to a bottle of CO_2_. The pressure was fixed with a manometer.

^*b*^Yields of isolated products after column chromatography on silica gel.

^*c*^The same result was obtained when MeCN or MeNO_2_ were used as solvents. [PdCl_2_(dppf)] = dichloro[1,1′-bis-(diphenylphosphino)-ferrocene]palladium(ii).

With the optimized reaction conditions in hand, we set out to explore the scope of this transformation (Scheme 2). The nature of the aryl iodide was explored first. Propargylamine **1**, reacted with aryl iodides bearing electron-withdrawing groups to give the corresponding oxazolidinones **3b–e** in excellent yields. Additional electron density in the aromatic ring appeared to be counterproductive given the reduced efficiency of the reaction to produce **3f**. Single crystals of compound **3c** allowed the unambiguous confirmation of the product structure and confirmed the *E* geometry of the exocyclic double bond.^18^

**Scheme 2:**
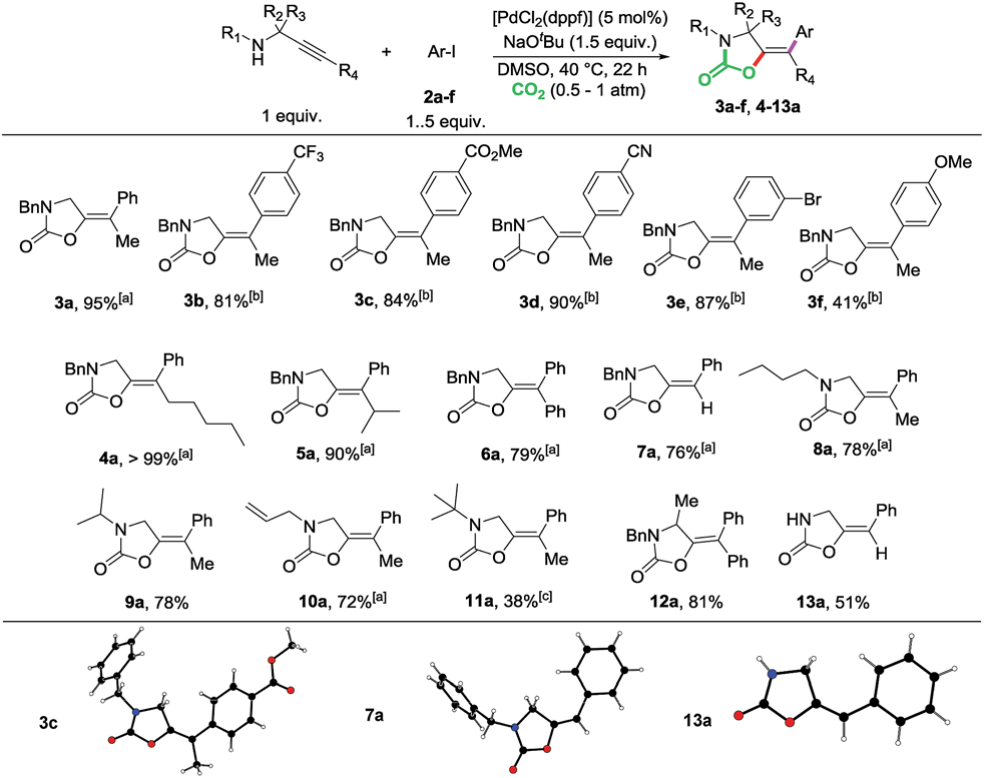
Scope for the carboxylative cyclization and cross-coupling reaction of propargylamines and aryl iodides. Yields of isolated products after column chromatography on silica gel are given. ^*a*^The reaction was performed with 1.1 equiv. of base. ^*b*^Yields obtained using [Pd] 2.5 mol%. ^*c*^The reaction was run for 3 days.

The reaction scope on the propargylamine substrates was explored next. Both alkyl (pentyl- and isopropyl-) as well as aryl-substituted alkynes were tolerated, delivering the corresponding cross coupling products **4–6a** in excellent yields. Terminal alkynes were also accommodated as demonstrated by the reaction to produce **7a** which could be isolated in 76% yield as a single isomer, as determined by X-ray diffraction analysis.^18^ Different substituents on the N-atom were also explored. While *N*-butyl, *N*-isopropyl and *N*-allyl propargylamines produced the corresponding oxazolidinones **8–10a** in good yields, the more sterically demanding *N-tert*-butyl substrate required longer reaction times for a productive outcome (**11a**). Substituents at the propargylic position were also well tolerated as demonstrated by the reaction to produce **12a**. Even commercially available propargylamine could be transformed into **13a** in 51% yield. The exquisite reaction stereocontrol could also be confirmed by X-ray diffraction analysis of this compound.^18^

Given the broad functional group tolerance observed in this carboxylative cyclization/cross-coupling reaction, we questioned whether an additional Csp–Csp^2^ cross coupling reaction could be incorporated in these multicomponent transformations to introduce, *in situ*, a substituent on the terminal position of the propargylamine substrates. To this end, we set out to explore the reaction between *N*-benzyl propargylamine **14** and three equivalents of iodobenzene (**2a**) in the presence of an additional Cu-cocatalyst. The reaction required a re-optimization of the reaction conditions as summarized in Table 2.^17^ To our delight, the reaction in the presence of 10 mol% of CuI at 60 °C delivered the desired product **6a** in 37% yield, although substantial amounts of the direct carboxylative cyclization and cross-coupling reaction (**7a**) could also be observed in this transformation (Table 2, entry 1).

**Table 2:** Optimization of the reaction conditions for the Sonogashira-carboxylative cyclization and cross-coupling reaction of propargylamine **14** and iodobenzene **2a**^*a*^

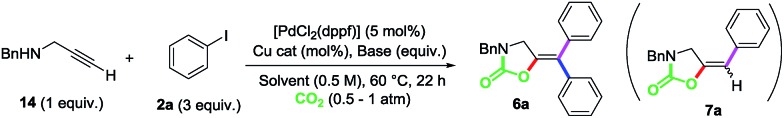				
Entry	Solvent	Cu cat. (mol%)	Base (equiv.)	**6a**, yield^*b*^ [%]
1	DMSO	CuI (10)	NaO*t*Bu (3)	37^*c*^
2	DMSO	CuI (10)	DBU (3)	—
3	DMSO	CuI (10)	Quinuclid. (3)	63
4	DMSO	CuI (10)	BEMP (3)	70 (65)
5	DMSO	CuI (10)	DABCO (3)	70 (64)
6	DMSO	CuI (5)	DABCO (2.6)	67 (65)
7	DMSO	CuTC (5)	DABCO (2.6)	68
8	DMSO	CuOAc (5)	DABCO (2.6)	70
9	DMSO	Cu-3-Me-salicyl (5)	DABCO (2.6)	72
**10**	**DMSO**	**CuI (5)**	**DABCO (2.6)**	**73 (71)** ^*d*^

^*a*^The reactions were performed in a Schlenk tube directly connected to a bottle of CO_2_. The pressure was fixed with a manometer.

^*b*^Yield determined by ^1^H-NMR spectroscopy using 1,2-dibromoethane as the internal standard. In brackets, isolated yields after column chromatography on silica gel.

^*c*^The by-product **7a** could be isolated in a significant amount.

^*d*^Concentration = 0.1 M. DABCO = 1,4-diazabicyclo[2.2.2]octane. DBU = 1,8-diazabicyclo[5.4.0]undec-7-ene. BEMP = 2-*tert*-butylimino-2-diethylamino-1,3-dimethylperhydro-1,3,2-diazaphosphorine.

Different bases were explored (Table 2, entries 2–5) of which DABCO turned out to be the most suitable and cost-effective one providing **6a** in a remarkable 64% isolated yield (Table 2, entry 5). A comparable efficiency was displayed when the Cu catalyst load was reduced to 5 mol% and the amount of base was adjusted to 2.6 equivalents (Table 2, entry 6). Copper salts such as CuTC, CuOAc and Cu-3-methylsalicylate offered comparable performances (Table 2, entries 7–9). Final adjustment of concentration and reaction time afforded compound **6a** in 71% isolated yield providing the optimal reaction conditions for this transformation (Table 2, entry 10).

Next, we set out to explore the scope of this multicomponent reaction (Scheme 3). Propargylamine substrates modified at both the C-backbone and the N-protecting group were well tolerated as shown by the reactions to produce compounds **6a**, **12a**, **15** and **16**. Taking **14** as a benchmark propargyl amine, different aryl iodides were explored. *meta*-Substituted-OMe, -OMOM, -OBn and -Me substrates could be efficiently coupled as shown by compounds **17–20**. The reaction is highly chemoselective, as demonstrated by the reaction to produce **21**, in which the Csp^2^–Br bond did not participate in the cross-coupling process. Di- and trimethoxy as well as 4-OMe and 4-methyl substituted 1-iodobenzenes could also be efficiently incorporated as demonstrated by the competent reactions to produce oxazolidinones **22–25**. Heteroaromatic rings could also be incorporated, as shown by the reaction to obtain bis-thiophene derivative **26**.

**Scheme 3:**
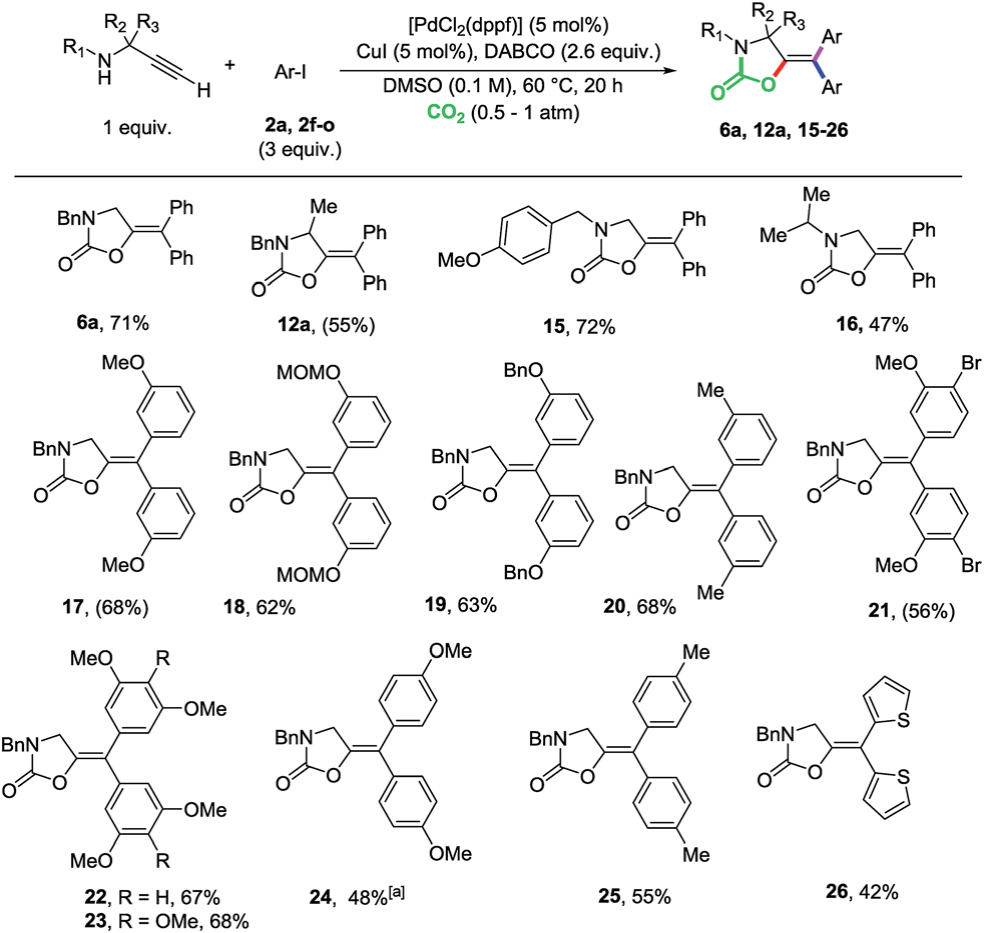
Scope for the Sonogashira-carboxylative cyclization and cross-coupling reaction of propargylamines and aryl iodides. Isolated yields after column chromatography are given. In brackets, yields determined by ^1^H-NMR spectroscopy using 1,2-dibromoethane as the internal standard. ^*a*^The reaction was performed with a 0.5 M concentration.

Several control experiments were designed in order to interrogate the reaction mechanism (Scheme 4).^17^ The reaction of propargylamines **1** and **27** with one equivalent of [PhPd(dppf)I]^19^ (**28**) under the standard conditions produced the corresponding oxazolidinones **3a** and **6a** in good yields respectively (eqn (1), right). When **1** reacted in the presence of a catalytic amount of **28**, a successful transformation into **3a** was also observed (eqn (1), left). As shown in eqn (2), disubstituted oxazolidinones such as **29** are not produced *via* Heck-reaction of monosubstituted ones. The reactions of *N*-benzyl propargylamine **14** with **2a**, 4-CF_3_– (**2b**) and 4-OMe-1-iodobenzene (**2f**) under the standard conditions from Table 2, entry 10 in the absence of CO_2_ delivered the corresponding substituted alkynes **27**, **30** and **31** in a >99%, 79% and 69% yield, respectively, which confirms the feasibility of a Sonogashira cross-coupling under the reaction conditions (eqn (3A)). The reactions of **27**, **30** and **31** with iodobenzene **2a** under the same conditions but in the presence of CO_2_ delivered the corresponding oxazolidinones **6a**, **32** and **33** in high yields (eqn (3B)). When **30** reacted with 4-CF_3_-1-iodobenzene **2b**, symmetrically substituted oxazolidinone **34** could be isolated in 38% yield (eqn (3C)). With these experiments in hand, the following mechanistic proposal can be envisioned (Scheme 5).

**Scheme 4:**
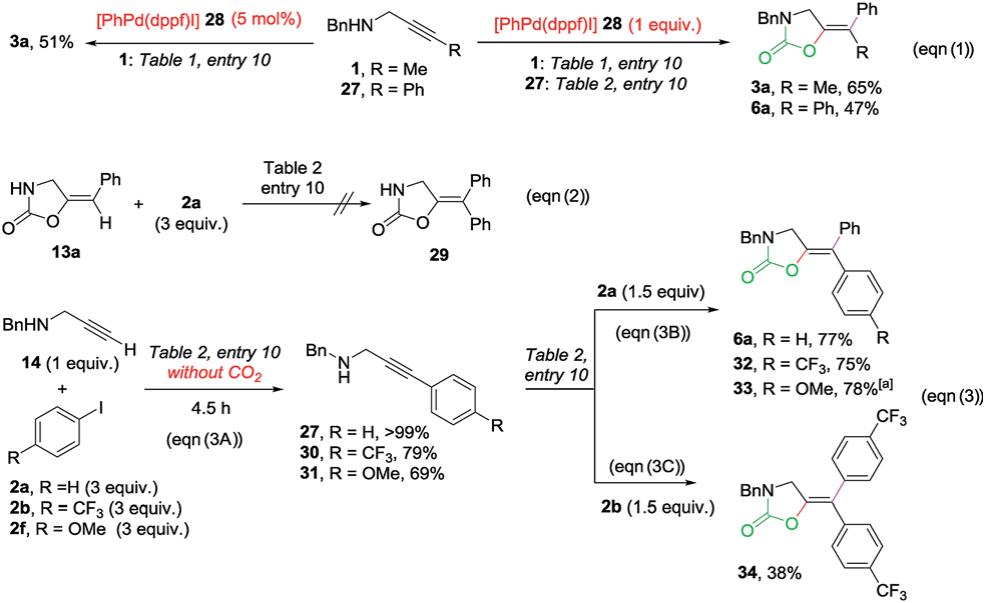
Control experiments. Isolated yields after column chromatography are given. ^*a*^Yield determined by ^1^H-NMR spectroscopy using 1,2-dibromoethane as the internal standard.

**Scheme 5:**
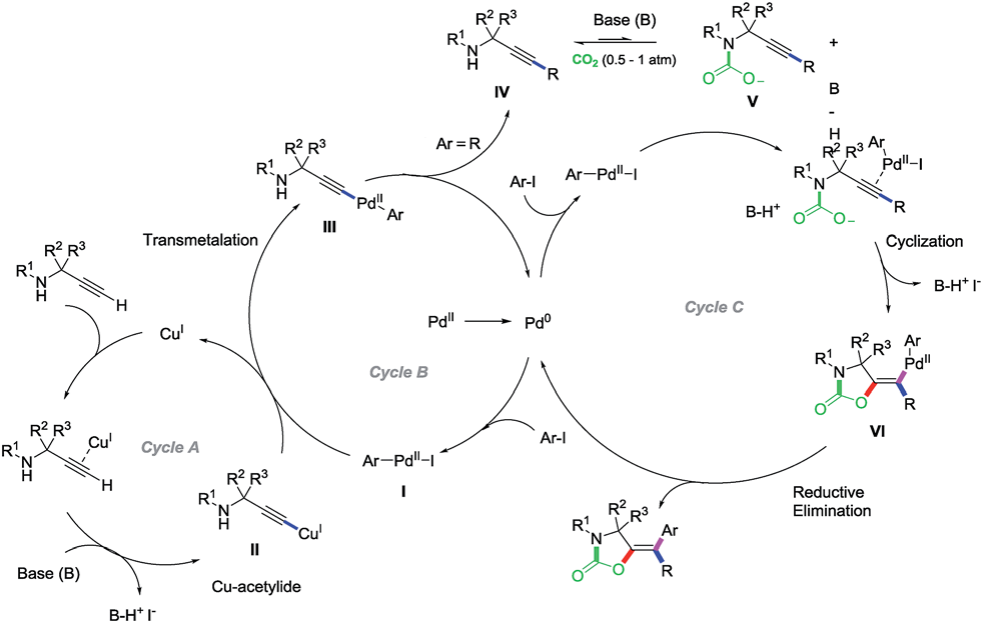
Mechanistic proposal.

*In situ* generated Pd(0)^20^ undergoes oxidative addition with aryl iodide present in the reaction media to give [ArPd(dppf)I] (**I**) (Cycle B). In the case of terminal alkynes, and in the presence of a catalytic amount of copper, transmetalation of **I** with copper acetylide **II** (Cycle A) delivers Pd–alkynyl intermediate **III**, which upon reductive elimination produces substituted propargylamine **IV**, as experimentally confirmed by the results reported in eqn (3A). Amine **IV** reacts with the base and CO_2_ to produce **V**^2^,10c,^21^ which reacts with additional **I** present in the reaction media to give, upon π-activation of the triple bond, vinyl palladium complex **VI**. Reductive elimination in **VI** delivers the observed oxazolidinone products (Cycle C). For starting materials with internal alkynes (Scheme 2), only *E* isomers were obtained as confirmed by the X-ray diffraction analysis of **3c**, **7a** and **13a**. Formation of **I** and its ability to catalyse the subsequent reaction steps is supported by experiments shown in eqn (1). The results collected in our control experiments provide evidence of the existence of a Csp–Csp^2^ coupling prior to the carboxylative-cyclization and Csp^2^–Csp^2^ cross-coupling, which indeed seems to be favourable with all kind of aryl iodides (neutral, electron-rich and electron-poor, eqn (3A)). In contrast, the cyclization process is sensitive to the electronic density on the active catalyst ([ArPd(dppf)I]), but not on the alkyne (carboxylative-cyclization & cross-coupling, eqn 3(B) and (C)). Together these results suggest that the cyclization process might be the responsible for the final outcome (yield) of this multicomponent reaction.

## Conclusions

In summary, two Pd-catalyzed multicomponent reactions producing highly functionalized 5-methylene-1,3-oxazolidin-2-ones from propargylamines and aryl iodides under mild conditions have been developed. These transformations employ CO_2_ at atmospheric pressure and provide a streamlined access to previously inaccessible versions of these useful heterocycles in a stereoselective and atom-economic manner. Further applications of these methodologies are currently being explored and will be reported in due course.

## Supplementary Material

Supplementary information

Crystal structure data
