# Supplementary material for: Palladium-catalyzed incorporation of atmospheric CO2: efficient synthesis of functionalized oxazolidinones
Source: Chem Sci. 2016 Mar 10;7(6):3914–8. doi: 10.1039/c6sc00419a (PMC6013789; doi:10.1039/c6sc00419a)
Supplement: Supplementary file 1 [file SC-007-C6SC00419A-s001.pdf]

Electronic Supplementary Material (ESI) for Chemical Science.
This journal is © The Royal Society of Chemistry 2016

Supporting Information

Palladium-Catalyzed Incorporation of Atmospheric CO_2: Efficient Synthesis of Functionalized Oxazolidinones

*Patricia García-Domínguez, Lorenz Fehr, Giulia Rusconi, and Cristina Nevado**

Table of Contents

1. General Information S3

2. Synthesis and Characterization of Substrates S5

3. General Procedures and Reaction Optimization S11

4. Control Experiments S19

5. Characterization of Products S22

6. ^{1}H and ^{13}C Spectra of all New Compounds S38

7. X-Ray Structure of Oxazolidinones **3c**, **7a** and **13a** S71

8. References S77

1. General information

NMR spectra were recorded on AV2 300, AV2 400 or AV2 500 MHz Bruker spectrometers. Chemical shifts are given in ppm. The spectra are calibrated to the residual ^{1}H and ^{13}C signals of the solvents. Multiplicities are abbreviated as follows: singlet (s), doublet (d), triplet (t), quartet (q), heptuplet (hept), multiplet (m), doublet-doublet (dd), doblet-triplet (dt), quartet-doublet (qd), doublet-doublet-doublet (ddd) and triplet-triplet (tt). Infrared spectra were recorded on a JASCO FT/IR-4100 spectrometer. Melting points were determined on a Büchi B-540 melting point instrument. High-resolution electrospray ionization and electronic impact mass spectrometry was performed on a Finnigan MAT 900 (Thermo Finnigan, San Jose, CA; USA) double focusing magnetic sector mass spectrometer. Ten spectra were acquired. A mass accuracy ≤ 2 ppm was obtained in the peak matching acquisition mode by using a solution containing 2 <l PEG200, 2 <l PPG450, and 1.5 mg NaOAc (all obtained from Sigma-Aldrich, CH-Buchs) dissolved in 100 mL MeOH (HPLC Supra grade, Scharlau, E-Barcelona) as internal standard. GC-MS analysis was done on a Finnigan Voyager GC8000 Top.

Materials and Methods: All reactions, unless otherwise stated, were carried out under inert gas atmosphere using standard Schlenk-techniques. All reagents were purchased from Aldrich, Fluorochem, ABCR, Across, Alfa Aesar, Fluka, TCI, Strem and/or Apollo and were used as received unless otherwise noted. Solvents were purchased in HPLC quality. DMSO, DCE, MeOH, glyme, $MeNO_2$ and Et_3N were degassed by purging thoroughly with nitrogen, dried according to published methods and distilled over activated molecular sieves of appropriate size before use. Alternatively, Alfa Aesar MeOH anhydrous 99.9% packaged under argon in a resealable ChemSeal™ bottle was used. Toluene, MeCN, THF, CH_2Cl_2, DMF and Et_2O were purged with argon and passed through alumina columns in a solvent purification system (Puresolv™, Innovative Technology). DMA anhydrous 99% was purchased from Sigma Aldrich. Reactions were monitored by thin layer chromatography (TLC) using Merck TLC silica gel 60 F254. Compounds were visualized by UV-light at 254 nm and by staining with an ethanolic solution of phosphomolybdic acid. Flash column chromatography was

performed over silica gel (230-400 mesh). CO_2 was purchased from by PanGas with a purity of 3.0.

<u>Catalysts</u>:

[$PdCl_2$(dppf)] was purchased from ABCR (99.9% purity) and used as received.

CuI was purchased from Aldrich (98% purity) and purified using the methods described in literature.[1]

2. Synthesis and Characterization of Substrates

2.1 Preparation of starting materials

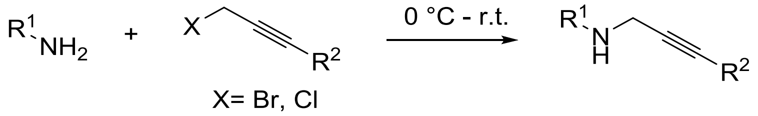

General Procedure for the synthesis of propargylamines.[2] For the preparation of propargyl amines, the propargyl halide (1 equiv.) was added dropwise to the corresponding amine (6 equiv.) at 0°C. Upon complete addition, the reaction was allowed to warm to room temperature and stirred for 17 h. Then, aqueous 1 M NaOH (4 mL/mmol) and Et_2O (4 mL/mmol) were added and the layers were separated. After extraction of the aqueous layer with Et_2O (2 x 4 mL/mmol), the combined organic layers were washed with brine, dried over $MgSO_4$ and the solvent was removed under reduced pressure. The crude was purified by flash column chromatography (silica gel, hexane/EtOAc). Spectral data were consistent with those previously reported.

***N*-Benzylbut-2-yn-1-amine (1)**[3]

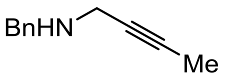

Following the general procedure, the reaction of 1-bromobut-2-yne (0.13 mL, 0.2 g, 1.5 mmol) and benzylamine (0.99 mL, 0.97 g, 9.02 mmol) afforded, after purification by flash column chromatography (silica gel, hexane/EtOAc, 80:20), 0.21 g (88%) of the titled compound as a colourless oil.

***N*-Benzyloct-2-yn-1-amine**[4]

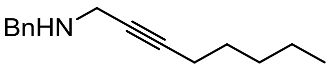

Following the general procedure, the reaction of 1-bromo-2-octyne (0.42 mL, 0.50 g, 2.64 mmol) and benzylamine (1.72 mL, 1.69 g, 15.9 mmol) afforded, after purification by flash column chromatography (silica gel, hexane/EtOAc, 80:20), 0.44 g (76%) of the titled compound as a pale yellow oil.

***N*-Benzyl-3-phenylprop-2-yn-1-amine (27)**[5]

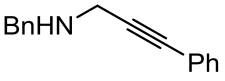

Following the general procedure, the reaction of 3-phenyl propargyl chloride (0.46 mL, 0.50 g, 3.32 mmol) and benzylamine (2.16 mL, 2.12 g, 19.9 mmol) afforded, after purification by flash column chromatography (silica gel, hexane/EtOAc, 80:20), 0.65 g (88%) of the titled compound as a yellow oil.

***N*-Benzylprop-2-yn-1-amine (14)**[5]

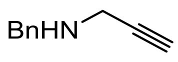

Following the general procedure, the reaction of propargyl bromide (0.50 g, 4.20 mmol) and benzylamine (2.75 mL, 2.70 g, 25.2 mmol) afforded, after purification by flash column chromatography (silica gel, hexane/EtOAc, 60:40), 0.48 g (79%) of the titled compound as a pale yellow oil.

***N*-Butylbut-2-yn-1-amine**[6]

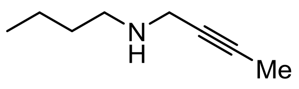

Following the general procedure described above for the synthesis of progargylamines, the reaction of 1-bromobut-2-yne (0.26 mL, 0.40 g, 3.01 mmol) and butylamine (1.78 mL, 1.32 g, 18.0 mmol) afforded, after purification by flash column chromatography (silica gel, hexane/EtOAc/Et_3N, 85:14:1), 0.28 g (73%) of the titled compound as a pale yellow oil.

***N*-(*iso*-Propyl)but-2-yn-1-amine**[7]

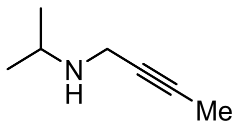

Following the general procedure, the reaction of 1-bromobut-2-yne (0.53 mL, 0.80 g, 6.02 mmol) and isopropylamine (3.1 mL, 2.13 g, 36.1 mmol) in CH_2Cl_2 (1 mL) for 2 days afforded, after purification by flash column chromatography (silica gel, hexane/EtOAc/Et_3N, 68:30:2), 0.37 g (55%) of the titled compound as a pale yellow oil.

***N*-Allylbut-2-yn-1-amine**[8]

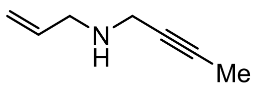

Following the general procedure, the reaction of 1-bromobut-2-yne (0.33 mL, 0.50 g, 3.76 mmol) and allylamine (1.70 mL, 1.29 g, 22.6 mmol) for 3 days afforded, after purification by flash column chromatography (silica gel, hexane/EtOAc/Et_3N, 80:19:1), 0.23 g (30%) of the titled compound as a pale yellow oil. Spectral data was consistent with a literature report.

***N*-(*tert*-Butyl)but-2-yn-1-amine**[9]

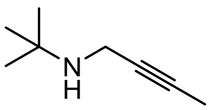

Following the general procedure, the reaction of 1-bromobut-2-yne (0.39 mL, 0.60 g, 4.51 mmol) and tertbutylamine (2.84 mL, 1.98 g, 27.1 mmol) in CH_2Cl_2 (1 mL) for 4 days afforded, after purification by flash column chromatography (silica gel, hexane/EtOAc/Et_3N, 95:3:2), 0.27 g (48%) of the titled compound as a colourless oil.

***N*-(*iso*-Propyl)prop-2-yn-1-amine**[10]

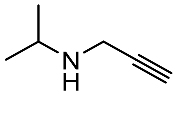

Following the general procedure, the reaction of propargyl bromide (3.0 g, 25.22 mmol) and isopropylamine (13.0 mL, 8.94 g, 151.31 mmol) afforded, after purification by flash column chromatography

(silica gel, hexane/EtOAc, from 70:30 to 40:60), 0.25 g (10%) of the titled compound as a colourless oil.

***N*-(4-methoxybenzyl)prop-2-yn-1-amine**[11]

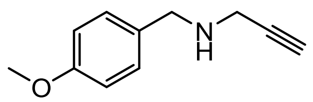

Following the general procedure, the reaction of propargyl bromide (0.78 g, 6.60 mmol) and 4-methoxybenzylamine (3.45 mL, 3.62 g, 26.4 mmol) afforded, after purification by flash column chromatography (silica gel, hexane/EtOAc, 80:20), 0.72 g (62%) of the titled compound as a pale yellow oil.

***N*-Benzyl-3-(*iso*-propyl)prop-2-yn-1-amine**

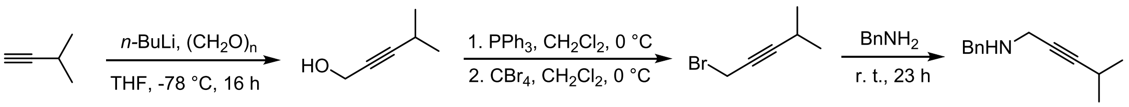

n-BuLi (3.8 mL, 1.6 M in hexane, 6.08 mmol) was added dropwise to a solution of 3-methylbut-1-yne (0.75 mL, 0.50 g, 7.3 mmol) in THF (7.5 mL) at –78 °C. After the mixture was stirred at this temperature for 45 min, dry paraformaldehyde (0.31 g, 10 mmol) was added. Then, the mixture was allowed to warm to 25 °C. and stirred for 16 h. A saturated aqueous solution of NH_4Cl (8 mL) was added and the aqueous layer was extracted with Et_2O (3x 20 mL). The combined organic layers were washed with brine (1 x 60 mL), dried over $MgSO_4$ and the solvent was removed under reduced pressure. The crude propargyl alcohol (0.58 g) was used in the next step without purification.

^{1}H-NMR (400 MHz, $CDCl_3$): δ 4.24 (d, *J* = 2.0 Hz, 2H), 2.67-2.50 (m, 1H), 1.16 (d, *J* = 6.9 Hz, 6H).

To a solution of the previous crude (0.58 g, 5.8 mmol) and PPh_3 (2.3 g, 8.7 mmol) in CH_2Cl_2 (12 mL) at 0 °C, a solution of CBr_4 (2.9 g, 8.7 mmol) in CH_2Cl_2 (7.3 ml) was added dropwise over 30 min. After the mixture was stirred at 0 °C for 2 h, it was diluted with hexane (20 mL), filtered through a pad of silica gel and the solvent removed under reduced pressure to give a propargyl bromide (0.61 g, 3.8 mmol) that was used in the next step without further purification.

^{1}H-NMR (400 MHz, $CDCl_3$): δ 3.93 (d, *J* = 2.2 Hz, 2H), 2.70-2.53 (m, 1H), 1.16 (d, *J* = 6.9 Hz, 6H).

Following the general procedure described above for the synthesis of propargylamines, the reaction of the propargyl bromide (0.61 g, 3.8 mmol) and benzylamine (2.5 g, 23 mmol) afforded, after purification by flash column chromatography (silica gel,

hexane/EtOAc, 80:20), 0.22 g (20%, over three steps) of the titled compound as a pale yellow oil.

^{1}H-NMR (400 MHz, $CDCl_3$): δ 7.37-7.29 (m, 4H), 7.28-7.23 (m, 1H), 3.86 (s, 2H), 3.40 (d, J = 2.0 Hz, 2H), 2.64-2.52 (m, 1H), 1.18 (d, J = 6.9 Hz, 6H).

***N*-Benzyl-4-phenylbut-3-yn-2-amine**[12]

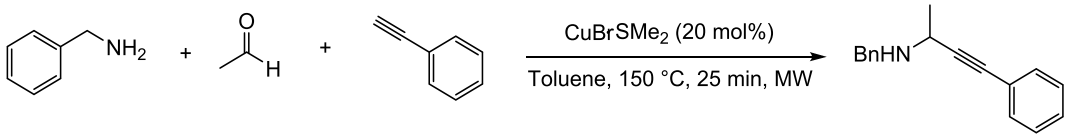

Phenylacetylene (0.54 mL, 0.50 g, 4.9 mmol), benzylamine (0.43 mL, 0.42 g, 4.0 mmol) and acetaldehyde (0.16 mL, 0.13 g, 3.0 mmol) were added to a suspension of CuBr • SMe_2 (0.13 g, 0.61 mmol) in toluene (61 mL). The mixture was heated by MW irradiation at 150 °C for 25 min and allowed to cool to room temperature. Then, the resulting slurry was concentrated under reduced pressure, providing the crude product. Purification by flash column chromatography (silica gel, hexane/EtOAc, 80:20) afforded 0.06 g (14%) of the propargyl amine as a pale orange oil. **^{1}H-NMR** (400 MHz, $CDCl_3$): δ 7.49-7.44 (m, 2H), 7.42-7.24 (m, 8H), 4.10 (d, J = 12.8 Hz, 1H), 3.91 (d, J = 12.8 Hz, 1H), 3.74 (q, J = 6.8 Hz, 1H), 1.47 (d, J = 6.8 Hz, 3H).

***N*-Benzylbut-3-yn-2-amine**[13]

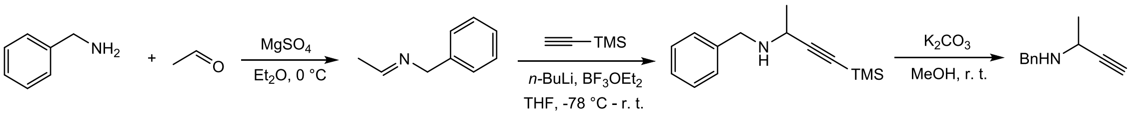

Benzylamine (3.06 mL, 3.0 g, 28 mmol) and acetaldehyde (1.91 mL, 1.5 g, 34 mmol) were added to a suspension of $MgSO_4$ (4.0 g, 33.23 mmol) in Et_2O (30 mL) at 0 ºC. The mixture was stirred at 0 ºC for 14 h. The solution was filtered and the filtrate was concentrated under reduced pressure to give a crude (3.3 g) which was used in the next step without further purification.

^{1}H-NMR (400 MHz, $CDCl_3$): δ 7.84 (d, J = 4.7 Hz, 1H), 7.37 – 7.20 (m, 5H), 4.56 (s, 2H), 2.02 (d, J = 4.8 Hz, 3H).

n-BuLi (4.9 mL, 1.6 M in hexane, 7.8 mmol) was added dropwise to a solution of trimethylsilylacetylene (1.11 mL, 0.77 g, 7.8 mmol) in THF (20 mL) at –78 °C. After stirring at this temperature for 30 min, a solution of the crude (0.80 g, 6.0 mmol) in THF (4.5 mL) was added. Subsequently, BF_3OEt_2 (1.7 g, 12 mmol) was added and the mixture was warmed up for 1 h from – 78 to 25 °C and stirred one more hour at room

temperature. The mixture was quenched with 2M NaOH (5 mL) and the aqueous layer extracted with Et_2O (3 x 5 mL). The combined organic layers were washed with brine (1 x 20 mL), dried over $MgSO_4$ and the solvent was removed under reduced pressure. The crude was purified by flash column chromatography (silica gel, hexane/EtOAc, 93:7) to afford the desired product (0.50 g, 37%) as a pale yellow oil.

^{1}H-NMR (400 MHz, $CDCl_3$): δ 7.38 – 7.29 (m, 4H), 7.28 – 7.22 (m, 1H), 4.00 (d, *J* = 12.7 Hz, 1H), 3.81 (d, *J* = 12.8 Hz, 1H), 3.49 (q, *J* = 6.8 Hz, 1H), 1.36 (d, *J* = 6.8 Hz, 3H), 0.19 (s, 9H).

The propargyl amine (0.25 g, 1.1 mmol) obtained in the previous step was added to suspension of K_2CO_3 (0.22 g, 1.6 mmol) in MeOH (3.3 mL). After stirring for 24 h, H_2O (5 mL) was added and the aqueous layer was extracted with EtOAc (3 x 5 mL). The combined organic layers were washed with brine (1 x 20 mL), dried over $MgSO_4$ and the solvent was removed under reduced pressure. The crude was purified by flash column chromatography (silica gel, hexane/EtOAc, 85:15) to afford the desired product (0.13 g, 75%) as a pale yellow oil.

^{1}H-NMR (400 MHz, $CDCl_3$): δ 7.39-7.30 (m, 4H), 7.28-7.22 (m, 1H), 4.02 (d, *J* = 12.8 Hz, 1H), 3.82 (d, *J* = 12.8 Hz, 1H), 3.50 (qd, *J* = 6.8, 2.1 Hz, 1H), 2.32 (d, *J* = 2.1 Hz, 1H), 1.39 (d, *J* = 6.8 Hz, 3H).

2.2 Synthesis of aryl iodides

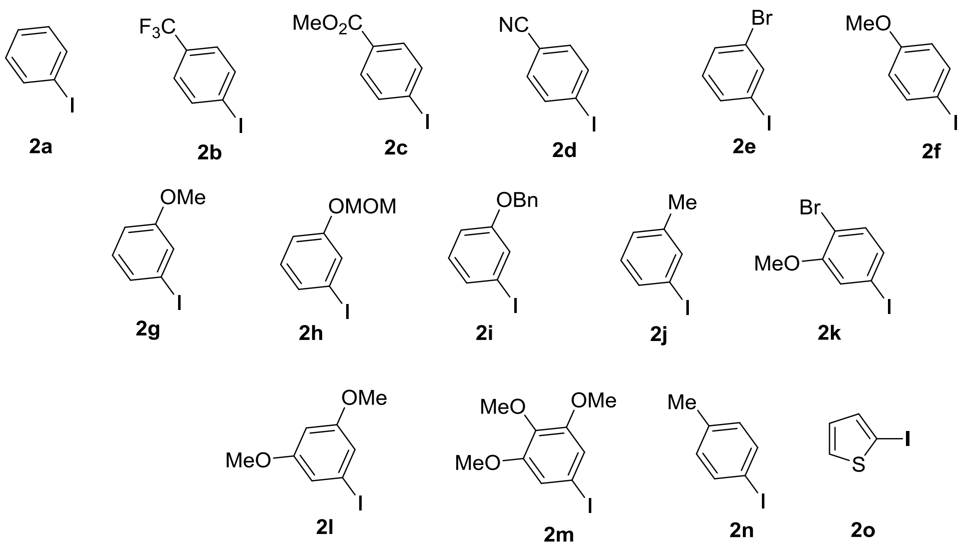

Aryl iodides **2a-2g**, **2j**, **2k**, **2m-o** were commercially available. **2h**,[14] **2i**[15] and **2l**[16] were prepared according to reported procedures.

3. General Procedures and Optimization of the Reaction Conditions

Reactions were run in Schlenk tubes with screw caps directly connected to a bottle of CO_2. The pressure was fixed with a manometer directly connected to the CO_2 bottle.

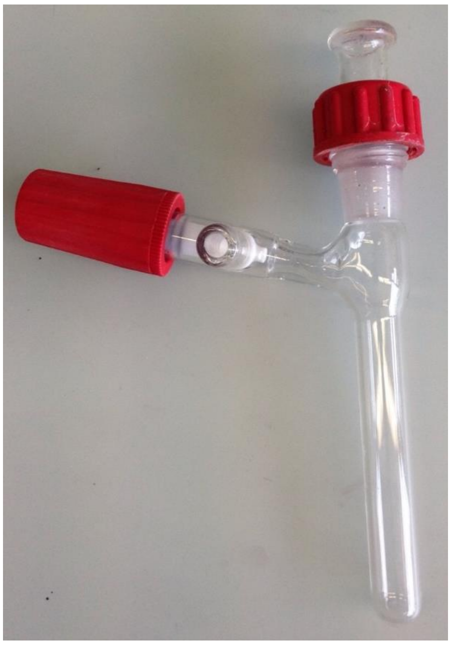

3.1 General procedures

Conditions A: carboxylative cyclization and cross-coupling reaction of propargylamines and aryl iodides

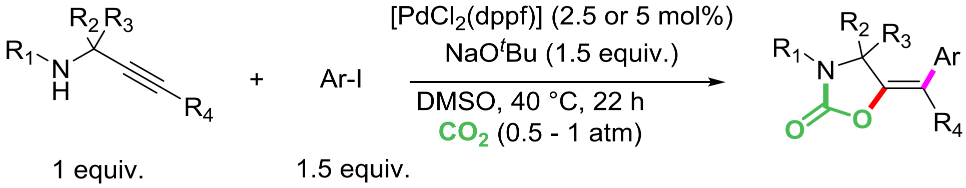

An oven-dried Schlenk tube with screw cap was connected to a bottle of CO_2, purged with this gas and charged with [PdCl$_2$(dppf)] (2.5 mol% or 5 mol%) and NaO^{t}Bu (1.5 equiv.). In a CO_2-purged vial the corresponding aryl iodide (1.5 equiv.) and propargyl amine (1.0 equiv.) were dissolved in DMSO previously degassed with CO_2 (0.50 M with respect to the limitant reagent). The resulting solution was transferred to the Schlenk tube containing the solids, the pressure of the gas was set up between 0.5-1.0 atm and the reaction mixture was stirred at 40 °C for 22 h. The reaction was quenched with H_2O (4 mL/mmol),) and diluted with EtOAc (4 mL/mmol). The layers were separated and the aqueous layer was extracted with EtOAc (2 x 4 mL/mmol). The combined organic layers were washed with brine (2 x 12 mL/mmol), dried over $MgSO_4$

and the solvent removed under reduced pressure. The crude was purified by flash column chromatography (silica gel, hexane/EtOAc).

Conditions B: Sonogashira-carboxylative cyclization and cross-coupling reaction of propargylamines and aryl iodides

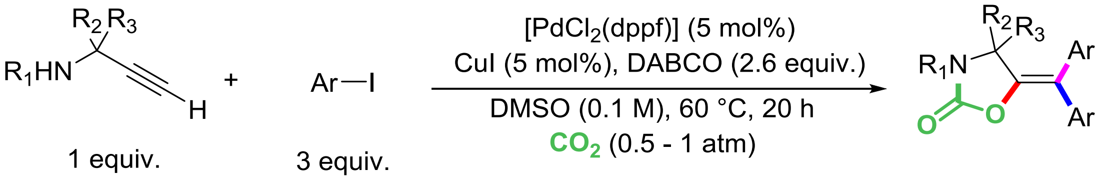

An oven-dried Schlenk tube with screw cap was connected to a bottle of CO_2, purged with this gas and charged with [$PdCl_2$(dppf)] (5 mol%), CuI (5 mol%) and DABCO (2.6 equiv.). In a CO_2-purged vial the corresponding aryl iodide (3.0 equiv.) and propargyl amine (1.0 equiv.) were dissolved in DMSO previously degassed with CO_2 (0.1M or 0.50 M with respect to the limitant reagent). The resulting solution was transferred to the Schlenk tube containing the solids, the pressure of the gas was set up between 0.5-1.0 atm and the reaction mixture was stirred at 60 °C for 20 h. The reaction was quenched with H_2O (4 mL/mmol),) and diluted with EtOAc (4 mL/mmol). The layers were separated and the aqueous layer was extracted with EtOAc (2 x 4 mL/mmol). The combined organic layers were washed with brine (2 x 12 mL/mmol), dried over $MgSO_4$ and the solvent removed under reduced pressure. The crude was purified by flash column chromatography (silica gel, hexane/EtOAc).

3.2 Optimization of the reaction conditions

3.2.1 Optimization for the carboxylative cyclization and cross-coupling reaction of propargylamines and aryl iodides[a]

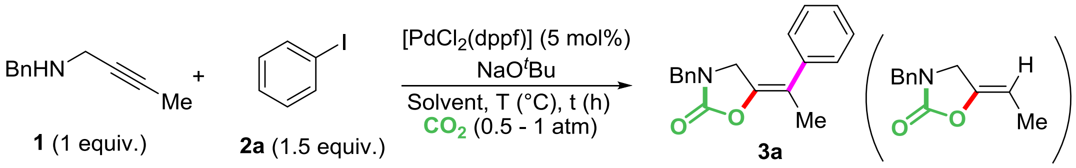

Entry	Solvent	Equivalents of NaO^{t}Bu	T [ºC]	t [h]	Yield [%]
1	THF	3	40	22	34[b]
2	THF	3[c]	40	44	47
3	DMF	3	40	44	57[e]
4	DMA	3	40	44	75[d][e]
5	DCE	3	40	44	Traces
6	$MeNO_2$	3	40	44	Traces

7	MeCN	3	40	44	Traces
8	DMSO	3	40	44	89[e]
9	DMSO	1.5	40	44	94
**10**	**DMSO**	**1.5**	**40**	**22**	**95**[f]
11	DMSO	1.1	40	22	87
12	DMSO	1.1	40	44	97
13	DMSO	1.1	r.t.	22	72
14	DMSO	1.1	r.t.	44	65

[a] The reactions were performed in a Schlenk tube directly connected to a bottle of CO_2. The pressure was fixed with a manometer. [b] When this reaction was performed with a balloon with CO_2 the yield decreased to 11%. [c] When a freshly prepared NaO^{t}Bu was used the yield of this entry dropped to 5% due to solubility issues with the base. [d] Yield determined by ^{1}H-NMR using 1,2-dibromoethane as internal standard. Otherwise, yields correspond to isolated products after column chromatography in Silica gel. [e] The protodemetallated product was isolated in small amount in these entries. [f] When DMSO was used as solvent the reactions were performed with a freshly prepared NaO^{t}Bu. [PdCl$_2$(dppf)] = Dichloro[1,1'-Bis(diphenylphosphino)ferrocene]palladium(II)

3.2.2 Optimization for the Sonogashira-carboxylative cyclization and cross-coupling reaction of propargylamines and aryl iodides[a]

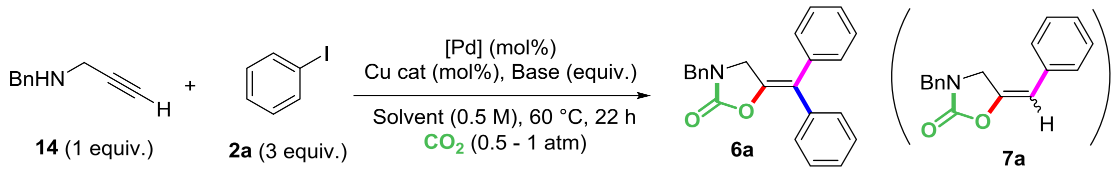

Entry	Solvent	Cu/Pd (mol%)	Base (equiv.)	T [°C]	Concentration [M]	t [h]	Yield [%][b] (Isolated Yield)	
							**6a**	**7a**
1	DMSO	CuI (10) [PdCl$_2$(dppf)] (10)	NaO^{t}Bu (3)	40	0.5	22	31	7 (***E*-7a**)
2	DMSO	CuI (10) [PdCl$_2$(dppf)] (10)	DBU (3)	40	0.5	22	-	50 (***Z*-7a**)
3	DMA	CuI (10) [PdCl$_2$(dppf)] (10)	NaO^{t}Bu (3)	40	0.5	22	29	11 (***E*-7a**)
4	DMF	CuI (10) [PdCl$_2$(dppf)] (10)	NaO^{t}Bu (3)	40	0.5	22	32	5 (***E*-7a**)
5	Et$_3$N	CuI (10) [PdCl$_2$(dppf)] (10)	NaO^{t}Bu (3)	40	0.5	22	15	41 (***Z*-7a**)
6	DMSO	CuI (10) [PdCl$_2$(dppf)] (10)	NaO^{t}Bu (3)	60	0.5	22	37	6 (***E*-7a**)
7	DMSO	CuBr (10) [PdCl$_2$(dppf)] (10)	NaO^{t}Bu (3)	60	0.5	22	30	13 (***E*-7a**)
8	DMSO	Cu$_2$O (10) [PdCl$_2$(dppf)] (10)	NaO^{t}Bu (3)	60	0.5	22	Trace	57 (***E*-7a**)

9	DMSO	CuSCN (10)<br>[$PdCl_2$(dppf)] (10)	NaO^{t}Bu (3)	60	0.5	22	28	9<br>(***E*-7a**)
10	DMSO	CuOTf (10)<br>[$PdCl_2$(dppf)] (10)	NaO^{t}Bu (3)	60	0.5	22	25	21<br>(***E*-7a**)
11	DMSO	CuI (10)<br>[$PdCl_2$(dppf)] (10)	KHMDS (3)	60	0.5	22	30	18<br>(***E*-7a**)
12	DMSO	CuI (10)<br>[$PdCl_2$(dppf)] (10)	DABCO (3)	60	0.5	22	70<br>(64)	traces
13	DMSO	CuI (10)<br>[$PdCl_2$(dppf)] (10)	DABCO (3)	60	0.5	44	65	traces
14	DMSO	CuI (10)<br>[$PdCl_2$(dppf)] (10)	DABCO (3)	60	0.5	12	58	traces
15	DMSO	CuI (10)<br>[$PdCl_2$(dppf)] (10)	DABCO (2.6)	60	0.5	22	67	traces
16	DMF	CuI (10)<br>[$PdCl_2$(dppf)] (10)	DABCO (2.6)	60	0.5	22	53	traces
17	Et_3N	CuI (10)<br>[$PdCl_2$(dppf)] (10)	DABCO (2.6)	60	0.5	22	26	traces
18	DMSO	CuI (10)<br>[$PdCl_2$(dppf)] (10)	BEMP (2.6)	60	0.5	22	70<br>(65)	traces
19	DMSO	CuI (10)<br>[$PdCl_2$(dppf)] (10)	Quinuclidine (2.6)	60	0.5	22	63	traces
20	DMSO	CuI (10)<br>[$PdCl_2$(dppf)] (5)	DABCO (2.6)	60	0.5	22	63	traces
**21**	**DMSO**	**CuI (5)**<br>**[$PdCl_2$(dppf)] (5)**	**DABCO (2.6)**	**60**	**0.5**	**22**	**67**<br>**(65)**	**traces**
22	THF	CuI (5)<br>[$PdCl_2$(dppf)] (5)	DABCO (2.6)	60	0.5	22	8	traces
23	DCE	CuI (5)<br>[$PdCl_2$(dppf)] (5)	DABCO (2.6)	60	0.5	22	4	traces
24	MeCN	CuI (5)<br>[$PdCl_2$(dppf)] (5)	DABCO (2.6)	60	0.5	22	56	traces
25	Toluene	CuI (5)<br>[$PdCl_2$(dppf)] (5)	DABCO (2.6)	60	0.5	22	6	traces
26	$MeNO_2$	CuI (5)<br>[$PdCl_2$(dppf)] (5)	DABCO (2.6)	60	0.5	22	45	traces
27	Glyme	CuI (5)<br>[$PdCl_2$(dppf)] (5)	DABCO (2.6)	60	0.5	22	30	traces
28	DMSO	CuI (5)<br>[$PdCl_2$(dppf)] (5)	Cs_2CO_3 (2.6)	60	0.5	22	28	traces
29	DMSO	CuI (5)<br>[$PdCl_2$(dppf)] (5)	$CsHCO_3$ (2.6)	60	0.5	22	34	traces
30	DMSO	CuI (5)<br>[$PdCl_2$(dppf)] (5)	CsOAc (2.6)	60	0.5	22	38	traces
31	DMSO	CuI (5)<br>[$PdCl_2$(dppf)] (5)	^{t}Bu-p4 (2.6)	60	0.5	22	0	traces
32	DMSO	CuI (5)<br>[$PdCl_2$(dppf)] (5)	HMPA (2.6)	60	0.5	22	0	traces
33	DMSO	CuI (5)<br>[$PdCl_2$(dppf)] (5)	HMDS (2.6)	60	0.5	22	0	traces
34	DMSO	CuI (5)<br>[$PdCl_2$(dppf)] (5)	TMEDA (2.6)	60	0.5	22	0	traces
35	DMSO	CuI (5)<br>[$PdCl_2$(dppf)] (5)	Pyridine (2.6)	60	0.5	22	0	traces

36	DMSO	CuI (5) [$PdCl_2$(dppf)] (5)	DBN (2.6)	60	0.5	22	40	traces
37	DMSO	CuI (5) [$PdCl_2$(dppf)] (5)	DMPU (2.6)	60	0.5	22	0	traces
38	DMSO	CuI (5) [$PdCl_2$(dppf)] (5)	Guanine (2.6)	60	0.5	22	0	traces
39	DMSO	CuI (5) [$PdCl_2$(dppf)] (5)	Cytosine (2.6)	60	0.5	22	0	traces
40	DMSO	CuI (5) [$PdCl_2$(dppf)] (5)	TBD (2.6)	60	0.5	22	38	traces
41	DMSO	CuI (5) [$PdCl_2$(dppf)] (5)	Tetramethylguanidine (2.6)	60	0.5	22	36	traces
42	DMSO	CuTC (5) [$PdCl_2$(dppf)] (5)	DABCO (2.6)	60	0.5	22	68	traces
43	DMSO	CuOAc (5) [$PdCl_2$(dppf)] (5)	DABCO (2.6)	60	0.5	22	72	traces
44	DMSO	Cu-3-methylsalicylate (5) [$PdCl_2$(dppf)] (5)	DABCO (2.6)	60	0.5	22	70	traces
45	DMSO	CuCN (5) [$PdCl_2$(dppf)] (5)	DABCO (2.6)	60	0.5	22	59	traces
46	DMSO	$CuPF_6(CN)_4$ (5) [$PdCl_2$(dppf)] (5)	DABCO (2.6)	60	0.5	22	56	traces
47	DMSO	CuSPh(5) [$PdCl_2$(dppf)] (5)	DABCO (2.6)	60	0.5	22	69	traces
48	DMSO	Cu_2S (5) [$PdCl_2$(dppf)] (5)	DABCO (2.6)	60	0.5	22	52	traces
49	DMSO	Copper (I)-diphenylphosphinate (5) [$PdCl_2$(dppf)] (5)	DABCO (2.6)	60	0.5	22	68	traces
50	DMSO	CuI (5) [$PdCl_2$(dppf)] (5)	DABCO (2.6)	60	1	22	55	traces
51	DMSO	CuI (5) [$PdCl_2$(dppf)] (5)	DABCO (2.6)	60	0.25	22	67	traces
52	DMSO	CuI (5) [$PdCl_2$(dppf)] (5)	DABCO (2.6)	60	0.10	22	74 (70)	traces
53	DMSO	CuI (5) [$PdCl_2$(dppf)] (5)	DABCO (2.6)	60	0.05	22	50	traces
54	DMSO	CuI (5) [$PdCl_2$(dppf)] (5)	DABCO (2.6)	60	0.5	4	63	traces
55	DMSO	CuI (5) [$PdCl_2$(dppf)] (5)	DABCO (2.6)	60	0.5	8	63	traces
56	DMSO	CuI (5) [$PdCl_2$(dppf)] (5)	DABCO (2.6)	60	0.5	12	66	traces
57	DMSO	CuI (5) [$PdCl_2$(dppf)] (5)	DABCO (2.6)	60	0.5	16	66	traces
58	DMSO	CuI (5) [$PdCl_2$(dppf)] (5)	DABCO (2.6)	60	0.5	20	65	traces
59	DMSO	CuI (5) [$PdCl_2$(dppf)] (5)	DABCO (2.6)	60	0.5	22	65	traces
60	DMSO	CuI (5) [$PdCl_2$(dppf)] (5)	DABCO (2.6)	60	0.5	30	66	traces

[a] The reactions were performed in a Schlenk tube directly connected to a bottle of CO_2. The pressure was fixed with a manometer. [b] Yield determined by ^{1}H-NMR spectroscopy using 1,2-dibromoethane as the internal standard. [c] **Z-7a** and the product resulting from the thermal isomerization to the internal alkene were the compounds obtained with this base. DABCO = 1,4-Diazabicyclo[2.2.2]octane. DBU = 1,8-Diazabicyclo[5.4.0]undec-7-ene. DBN = 1,5-Diazabicyclo[4.3.0]non-5-ene. BEMP = 2-*tert*-butylimino-2-diethylamino-1,3-dimethylperhydro-1,3,2-diazaphosphorine. [$PdCl_2$(dppf)] = Dichloro[1,1'-Bis-(diphenylphosphino)-ferrocene]palladium(II). Glyme = 1,2-dimethoxyethane. HMDS = Potassium hexamethyldisilazide. KHMDS = Potassium hexamethyldisilazide. HMPA = Hexamethylphosphoramide. TMEDA = *N*,*N*,*N′*,*N′*-Tetramethylethylenediamine. DMPU = 1,3-Dimethyl-2-oxohexahydropyrimidine. TBD = 1,5,7-Triazabicyclo[4.4.0]dec-5-ene.

Quinuclidine = 1-Azabicyclo[2.2.2]octane

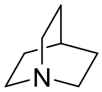

Phosphazene base P_4-*t*-Bu = 1-*tert*-Butyl-4,4,4-tris(dimethylamino)-2,2-bis[tris(dimethylamino)-phosphoranylidenamino]-2λ^5,4λ^5-catenadi(phosphazene).

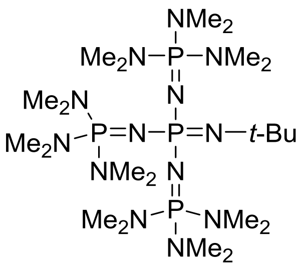

Cu-3-methylsalicylate

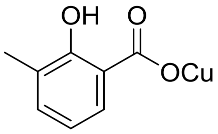

Final optimization with the combination of the best parameters:

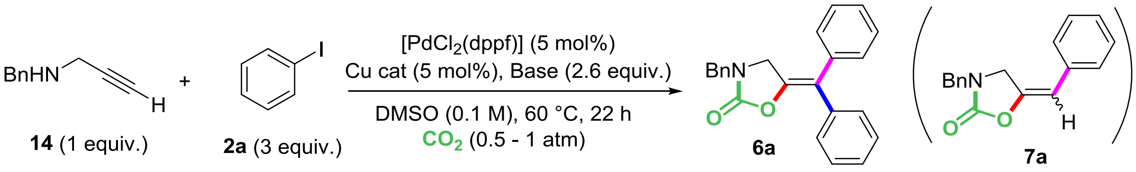

- Concentration: 0.1 M
- Bases: DABCO, BEMP
- Cu(I)-salt: Cu(TC), Copper(I)-3-methylsalicylate, CuOAc

Entry	Cu(I)-salt	Base	^{1}H-NMR yield[a]
1	Cu(TC)	DABCO	71%
2	Cu(I)-3-methyls.	DABCO	69%
3	CuOAc	DABCO	69%
4	Cu(TC)	BEMP	46%, (34% ***E*-7a**)
5	Cu(I)-3-methyls.	BEMP	55%
6	CuOAc	BEMP	64%

[a] Yield determined by ^{1}H-NMR spectroscopy using 1,2-dibromoethane as the internal standard.

Screening of palladium catalysts with the best conditions above for the Sonogashira-carboxylative cyclization and cross-coupling reaction of propargylamines and aryl iodides.

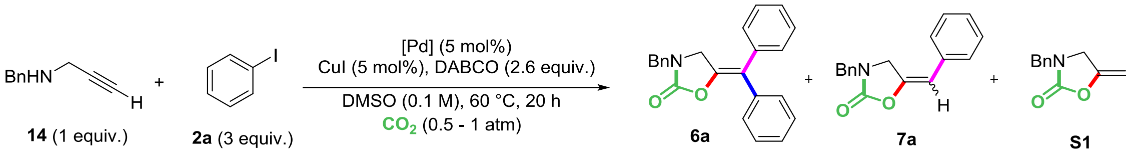

Entry	Pd catalysts	^{1}H-NMR Yield[a]			
		**6a**	***E*-7a**	***Z*-7a**	**S1**
1	**[PdCl$_2$(dppf)]**	**74%**	**4%**	**6%**	**-**
2	[PdCl$_2$(dcpf)C]	-	-	5%	75%
3	[PdCl$_2$(dtbpf)]	22%	21%	2%	26%
4	[PdCl$_2$(BPPFA)]	54%	7%	7%	-
5	[PdCl$_2$(dippp)]	46%	3%	19%	-
6	[PdCl$_2$(XantPhos)]	50%	3%	18%	-
7	[PdCl$_2$(DPEPhos)]	57%	10%	2%	-
8	[PdCl$_2$(quinox)]	27%	10%	7%	25%
9	[PdCl$_2$(Phen)]	6%	8%	3%	28%

[a] Yield determined by ^{1}H-NMR spectroscopy using 1,2-dibromoethane as the internal standard.

[PdCl$_2$(dppf)] = Dichloro[1,1'-Bis(diphenylphosphino)ferrocene]palladium(II)

[$PdCl_2$(dcypf)] = Dichloro[bis(2-(di-cyclohexylbutylphosphino)phenyl)ether]palladium(II)

[$PdCl_2$(dtbpf)] = Dichloro[bis(2-(di-*tert*-butylphosphino)phenyl)ether]palladium(II)

[$PdCl_2$([(*R*,Sp)-BPPFA)]= Dichloro[N,N-dimethyl-l [l',2-bis(diphenylphosphino)ferrocenyl]ethylamine) palladium(II)

[$PdCl_2$(dippp)] = Dichloro[1,3-bis(diphenylphosphino)propane]palladium(II)

[$PdCl_2$(XantPhos)] = Dichloro[9,9-dimethyl-4,5-bis(diphenylphosphino)xanthene]palladium(II)

[$PdCl_2$(DPEPhos)] = Dichloro[bis(2-(diphenylphosphino)phenyl)ether] palladium(II)

[$PdCl_2$[$PdCl_2$(quinox)] = Dichloro[2-(4,5-dihydro-2-oxazolyl)quinoline]palladium(II)

[[$PdCl_2$(Phen)] = Dichloro[1,10-phenanthroline]palladium(II)

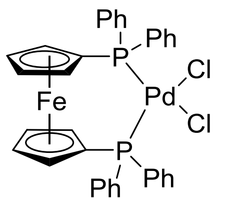

[$PdCl_2$(dtbpf)]

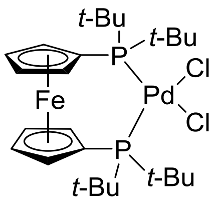

[$PdCl_2$(dtbpf)]

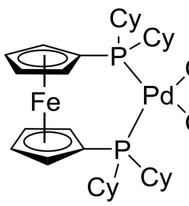

[$PdCl_2$(dcypf)]

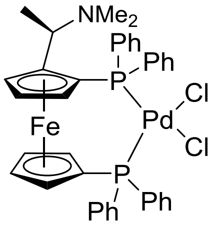

[$PdCl_2$(BPPFA)]

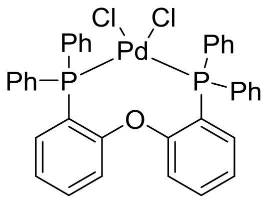

[$PdCl_2$(DPEPhos)]

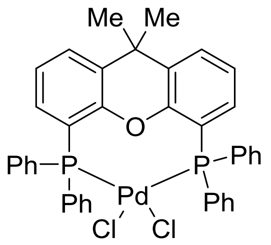

[$PdCl_2$(XantPhos)]

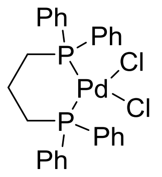

[$PdCl_2$(dippp)]

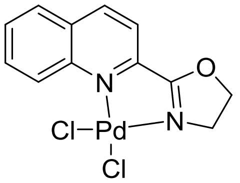

[$PdCl_2$(quinox)]

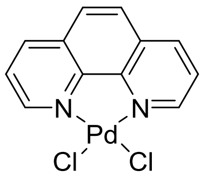

[$PdCl_2$(Phen)]

4. Control Experiments

N-Benzyl-3-[4-(trifluoromethyl)phenyl]prop-2-yn-1-amine (30)

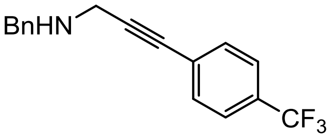

Conditions B: Following the general procedure described above for the Sonogashira-carboxylative cyclization and cross-coupling reaction of propargylamines and aryl iodides, the reaction of *N*-benzylprop-2-yn-1-amine (**14**) (0.2 g, 1.38 mmol), 4-iodobenzotrifluoride (1.12 g, 4.13 mmol), DABCO (0.40 g, 5.59 mmol), CuI (13.1 mg, 69.0 μmol) and [$PdCl_2$(dppf)] (0.05 g, 69.0 μmol) in DMSO (13.8 mL) at 60 ºC for 4.5 h afforded**,** after purification by flash column chromatography (silica gel, hexane/EtOAc, 80:20), 0.31 g (79%) of the titled compound as a pale yellow oil.

^{1}H-NMR (400 MHz, $CDCl_3$): δ 7.53-7.39 (m, 4H), 7.42 – 7.33 (m, 4H), 7.31 – 7.25 (m, 1H), 3.96 (s, 2H), 3.68 (s, 2H). **^{13}C-NMR** (100 MHz, $CDCl_3$): δ 139.3 (s), 131.9 (d), 129.9 (q, $^2J_{C\text{-}F}$= 32.6 Hz), 128.6 (d), 128.5 (d), 127.4 (d), 127.1 (s), 125.3 (q, $^3J_{C\text{-}F}$= 3.8 Hz), 124.0 (q, $^1J_{C\text{-}F}$= 272.2 Hz), 90.1 (s), 82.6 (s), 52.6 (t), 38.2 (t). **^{19}F-NMR** (376 MHz, $CDCl_3$): δ = -62.75 ppm. IR (neat): ν 3030, 2926, 2837, 1615, 1495, 1454, 1404, 1319, 1253, 1164, 1121, 1104, 1065, 1017, 946, 840, 734, 697, 596, 568, 542 cm^{-1}. **HRMS (ESI)** *m/z* calcd for $C_{17}H_{14}NF_3$ [M+H$^+$] 290.1156, found 290.1150.

N-Benzyl-3-(4-methoxyphenyl)prop-2-yn-1-amine (31)[17]

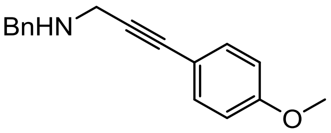

Conditions B: Following the general procedure described above for the Sonogashira-carboxylative cyclization and cross-coupling reaction of propargylamines and aryl iodides, the reaction of *N*-benzylprop-2-yn-1-amine **(14)** (0.25 g, 1.72 mmol), 4-iodoanisole (1.2 g, 5.16 mmol), DABCO (0.5 g, 4.47 mmol), CuI (16.4 mg, 86.0 μmol) and [$PdCl_2$(dppf)] (63.0 mg, 86.0 μmol) in DMSO (17 mL) at 60 ºC for 4.5 h afforded**,** after purification by flash column chromatography (silica gel, hexane/EtOAc, 70:30), 0.3 g (69%) of the titled compound as a pale yellow solid.

^{1}H-NMR (400 MHz, $CDCl_3$): δ 7.41 – 7.32 (m, 6H), 7.30 – 7.23 (m, 1H), 6.89 – 6.79 (m, 2H), 3.95 (s, 2H), 3.81 (s, 3H), 3.64 (s, 2H).

(*Z*)-3-Benzyl-5-[phenyl(4-(trifluoromethyl)phenyl)methylene]oxazolidin-2-one (32)

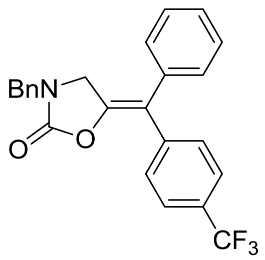

Following the general procedure described above for the Sonogashira-carboxylative cyclization and cross-coupling reaction of propargylamines and aryl iodides, the reaction of *N*-Benzyl-3-[4-(trifluoromethyl)phenyl]prop-2-yn-1-amine (**30**) (0.80 g, 0.28 mmol), iodobenzene (0.17 g, 0.84 mmol), DABCO (0.08 g, 0.73 mmol), CuI (2.7 mg, 14 μmol) and [PdCl$_2$(dppf)] (10.2 mg, 14 μmol) in DMSO (2.8 mL) at 60 ºC under CO_2 atmosphere (0.5-1.0 atm) afforded**,** after purification by flash column chromatography (silica gel, hexane/EtOAc, 80:20), 85.2 mg (75%) of the titled compound as a colourless solid. **^{1}H-NMR** (400 MHz, CDCl$_3$): δ 7.59-7.48 (m, 4H), 7.41 – 7.29 (m, 6H), 7.29 – 7.24 (m, 2H), 7.16 – 7.11 (m, 2H), 4.48 (s, 2H), 4.05 (s, 2H). **^{13}C-NMR** (100 MHz, CDCl$_3$): δ 155.4 (s), 141.5 (s), 140.4 (s), 137.1 (s), 134.9 (s), 129.8 (d), 129.4 (d), 129.3 (d), 129.2 (d), 128.8 (s), 128.4 (d), 128.2 (d), 128.2 (d), 125.1 (q, $^3J_{C-F}$= 3.7 Hz), 124.2 (d, $^1J_{C-F}$= 271.9 Hz), 115.6 (s), 48.4 (t), 48.0 (t). **^{19}F-NMR** (376 MHz, CDCl$_3$): δ = -62.58 ppm. IR (neat): ν 3031, 1788, 1668, 1616, 1471, 1419, 1325, 1261, 1165, 1119, 1067, 1050, 1017, 963, 845, 789, 749, 702, 548 cm^{-1}. **HRMS (ESI)** *m/z* calcd for $C_{24}H_{19}NO_2F_3$ [M+H$^+$] 410.1362, found 410.1358.

(*Z*)-3-Benzyl-5-[(4-methoxyphenyl)phenylmethylene]oxazolidin-2-one (33)

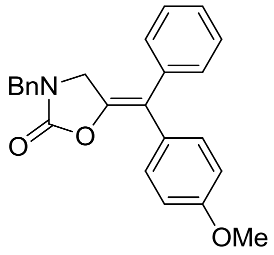

Following the general procedure described above for the Sonogashira-carboxylative cyclization and cross-coupling reaction of propargylamines and aryl iodides, the reaction of *N*-benzyl-3-(4-methoxyphenyl)prop-2-yn-1-amine (**31**) (0.60 g, 0.24 mmol), iodobenzene (0.15 g, 0.72 mmol), DABCO (0.07 g, 0.62 mmol), CuI (2.3 mg, 12 μmol) and [PdCl$_2$(dppf)] (8.8 mg, 12 μmol) in DMSO (2.4 mL) at 60 ºC under CO_2 atmosphere (0.5-1.0 atm) afforded**,** after purification by flash column chromatography (silica gel, hexane/EtOAc, 80:20), 69.5 mg (78% calculated by ^{1}H-NMR with 1,2-dibromoethane as internal standard) of the titled compound as a colourless liquid. **^{1}H-NMR** (400 MHz, CDCl$_3$): δ 7.40 – 7.23 (m, 10H), 7.16 – 7.12 (m, 2H), 6.85 – 6.80 (m, 2H), 4.47 (s, 2H), 4.01 (s, 2H), 3.79 (s, 3H). **^{13}C-NMR** (100 MHz, CDCl$_3$): δ 158.7 (s), 156.0 (s), 138.6 (s), 138.2 (s), 135.2 (s), 130.4 (d), 129.9 (d, 2 x CH), 129.5 (s), 129.1 (d), 128.3 (d), 128.2 (d), 127.8 (d), 116.6 (s), 113.6 (d), 55.4 (q), 48.4 (t), 48.0 (t). **IR** (neat): ν 3031, 2932, 2833, 1780, 1669,

1606, 1510, 1471, 1440, 1420, 1250, 1179, 1081, 1054, 959, 833, 784, 751, 702 cm^{-1}. **HRMS (ESI)** *m/z* calcd for $C_{24}H_{22}NO_3$ [M+H$^+$] 372.1954, found 272.1956.

3-benzyl-5-[bis(4-(trifluoromethyl)phenyl)methylene]oxazolidin-2-one (34)

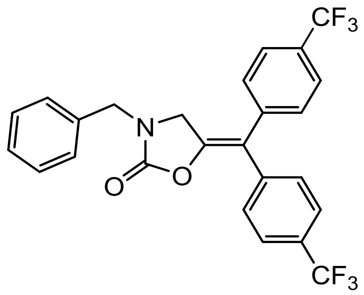

Following the general procedure described above for the Sonogashira-carboxylative cyclization and cross-coupling reaction of propargylamines and aryl iodides, the reaction of *N*-Benzyl-3-[4-(trifluoromethyl)phenyl]prop-2-yn-1-amine **(30)** (90 mg, 0.31 mmol), iodobenzotrifluoride (253 mg, 0.93 mmol), DABCO (91 mg, 0.8 mmol), CuI (2.9 mg, 15.0 μmol) and [PdCl$_2$(dppf)] (10.9 mg, 15.0 μmol) in DMSO (3.1 mL) at 60 ºC under CO_2 atmosphere (0.5-1.0 atm) afforded**,** after purification by flash column chromatography (silica gel, hexane/EtOAc, 90:10), 56 mg (38%) of the titled compound as a pale yellow solid. **M.p.** 158-160 ºC.^{1}**H-NMR** (400 MHz, CDCl$_3$): δ 7.60 – 7.52 (m, 2H), 7.50 – 7.43 (m, 2H), 7.37 (d, *J* = 8.2 Hz, 2H), 7.33 – 7.22 (m, 3H), 7.18 (ddd, *J* = 6.5, 2.9, 1.0 Hz, 4H), 4.40 (s, 2H), 3.96 (s, 2H). ^{13}**C-NMR** (100 MHz, CDCl$_3$): δ 155.0 (s), 142.2 (s), 140.9 (s), 139.7 (s), 134.7 (s), 130.4 (q, $^2J_{C-F}$= 33.0 Hz), 130.3 (d), 129.4 (d), 129.3 (q, $^2J_{C-F}$= 33.0 Hz), 129.2 (d), 128.6 (d), 128.3 (d), 126.3 (q, $^3J_{C-F}$= 3.7 Hz), 125.3 (q, $^3J_{C-F}$= 3.7 Hz), 124.2 (q, $^1J_{C-F}$= 272.0 Hz), 124.0 (q, $^1J_{C-F}$= 272.3 Hz), 114.6 (s), 48.3 (t), 48.1 (t). ^{19}**F-NMR** (376 MHz, CDCl$_3$): δ -62.66 (s), -62.72 (s). **IR** (neat): ν 1785, 1671, 1614, 1421, 1323, 1265, 1170, 1131, 1068, 1051, 1014, 962, 903, 845, 723, 649, 545 cm^{-1}. **HRMS (ESI)** *m/z* calcd for $C_{25}H_{18}F_6NO_2$ [M+H$^+$] 478.1242, found 478.1240.

5. Characterization of products

5.1 Carboxylative cyclization and cross-coupling reaction of propargylamines and aryl iodides

*Note: for compounds **3b-f**, similar yields were obtained when using 2.5 mol% Pd catalyst compared to the 5 mol% described standard conditions.*

(*E*)-3-Benzyl-5-(1-phenylethylidene)oxazolidin-2-one (3a)

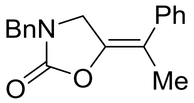

<u>**Conditions A**</u>**:** Following the general procedure described above for the carboxylative cyclization and cross-coupling reaction of propargylamines and aryl iodides, the reaction of *N*-benzylbut-2-yn-1-amine (**1**) (0.05 g, 0.31 mmol), iodobenzene (0.09 g, 0.46 mmol), NaO*t*Bu (0.03 g, 0.34 mmol) and [$PdCl_2$(dppf)] (11.3 mg, 15.5 μmol) in DMSO (0.62 mL) at 40 ºC under CO_2 atmosphere (0.5-1.0 atm) afforded**,** after purification by flash column chromatography (silica gel, hexane/EtOAc, 85:15), 83 mg (95%) of the titled compound as an orange oil. **^{1}H-NMR** (400 MHz, $CDCl_3$): δ 7.37-7.28 (m, 5H), 7.27-7.20 (m, 3H), 7.16-7.12 (m, 2H), 4.46 (s, 2H), 4.04 (q, *J* = 2.1 Hz, 2H), 2.09 (t, *J* = 2.1 Hz, 3H). **^{13}C-NMR** (100 MHz, $CDCl_3$): δ 156.0 (s), 139.1 (s), 138.7 (s), 135.3 (s), 129.1 (d), 128.8 (d), 128.3 (d), 128.2 (d), 127.4 (d), 127.3 (d), 112.4 (s), 48.1 (t), 47.5 (t), 16.6 (q). **IR** (neat): ν 3031, 2922, 2865, 1774, 1693, 1495, 1474, 1420, 1254, 1219, 1084, 1061, 1026, 753, 700, 678 cm^{-1}. **HRMS (ESI)** *m/z* calcd for $C_{18}H_{17}NO_2Na$ [M+Na$^+$] 302.11524, found 302.11515.

(*E*)-3-Benzyl-5-[1-(4-(trifluromethyl)phenyl)ethylidene]oxazolidin-2-one (3b)

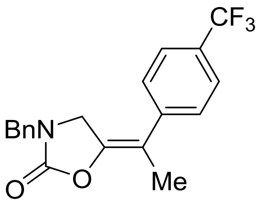

<u>**Conditions A**</u>**:** Following the general procedure described above for the carboxylative cyclization and cross-coupling reaction of propargylamines and aryl iodides, the reaction of *N*-benzylbut-2-yn-1-amine (**1**) (0.05 g, 0.31 mmol), 4-iodobenzotrifluoride (0.2 g, 0.46 mmol), NaO*t*Bu (0.04 g, 0.46 mmol) and [$PdCl_2$(dppf)] (5.7 mg, 7.75 μmol, 2.5 mol%) in DMSO (0.62 mL) at 40 ºC under CO_2 atmosphere (0.5-1.0 atm) afforded, after purification by flash column chromatography (silica gel, hexane/EtOAc, 85:15), 86 mg (81%) of the titled compound as yellow oil. **^{1}H-NMR** (400 MHz, $CDCl_3$): δ 7.56 (d, *J* = 8.1 Hz, 2H), 7.38-7.28 (m, 3H), 7.28-7.22 (m, 4H), 4.46 (s, 2H), 4.03 (q, *J* = 2.1 Hz, 2H), 2.10 (t, *J* = 2.1 Hz, 3H). **^{13}C-NMR** (100 MHz, $CDCl_3$): δ 155.6 (s), 142.8 (s), 139.9 (s), 135.1 (s), 129.3 (q, $^2J_{C-F}$= 32.7 Hz), 129.1 (d), 128.4 (d), 128.2 (d), 127.7 (d), 125.8 (q, $^3J_{C-F}$= 3.8 Hz), 124.1 (q, $^1J_{C-F}$= 272.0 Hz),

111.3 (s), 48.1 (t), 47.5 (t), 16.4 (q). ^{19}**F-NMR** (376 MHz, $CDCl_3$): δ - 62.6 (s). **IR** (neat): ν 1775, 1692, 1614, 1426, 1407, 1322, 1255, 1161, 1119, 1057, 1014, 843, 749, 702, 681, 656, 620 cm^{-1}. **HRMS (ESI)** *m/z* calcd for $C_{19}H_{16}F_3NO_2Na$ $[M+Na^+]$ 370.1025, found 370.1028.

Methyl (*E*)-4-[1-(3-benzyl-2-oxooxazolidin-5-ylidene)ethyl]benzoate (3c)

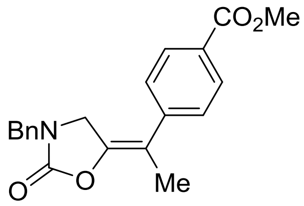

<u>Conditions A</u>: Following the general procedure described above for the carboxylative cyclization and cross-coupling reaction of propargylamines and aryl iodides, the reaction of *N*-benzylbut-2-yn-1-amine (**1**) (0.05 g, 0.31 mmol), methyl 4-iodobenzoate (0.12 g, 0.46 mmol), NaO*t*Bu (0.04 g, 0.46 mmol) and $[PdCl_2(dppf)]$ (5.7 mg, 7.75 μmol, 2.5 mol%) in DMSO (0.62 mL) at 40 ºC under CO_2 atmosphere (0.5-1.0 atm) afforded**,** after purification by flash column chromatography (silica gel, hexane/EtOAc, 80:20), 87 mg (84%) of the titled compound as colourless solid. **M.p.** 82-84 ºC. ^{1}**H-NMR** (400 MHz, $CDCl_3$): δ 8.02-7.91 (m, 2H), 7.38-7.28 (m, 3H), 7.27-7.23 (m, 2H), 7.23-7.19 (m, 2H), 4.46 (s, 2H), 4.05 (q, *J* = 2.1 Hz, 2H), 3.90 (s, 3H), 2.11 (t, *J* = 2.1 Hz, 3H). ^{13}**C-NMR (100 MHz, $CDCl_3$)**: δ 166.7 (s), 155.7 (s), 143.8 (s), 139.9 (s), 135.1 (s), 130.1 (d, 2 x CH), 129.1 (d), 128.9 (s), 128.4 (d), 128.3 (d), 127.3 (d), 111.7 (s), 52.3 (q), 48.1 (t), 47.6 (t), 16.3 (q). **IR** (neat): ν 3036, 2948, 1775, 1718, 1686, 1604, 1432, 1281, 1187, 1109, 1057, 775, 749, 702 cm^{-1}. **HRMS (ESI)** *m/z* calcd for $C_{20}H_{19}NO_4Na$ $[M+Na^+]$ 360.1206, found 360.1211.

(*E*)-4-[1-(3-Benzyl-2-oxooxazolidin-5-ylidene)ethyl]benzonitrile (3d)

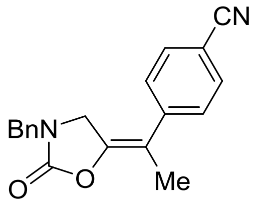

<u>Conditions A</u>: Following the general procedure described above for the carboxylative cyclization and cross-coupling reaction of propargylamines and aryl iodides, the reaction of *N*-benzylbut-2-yn-1-amine (**1**) (0.05 g, 0.31 mmol), 4-iodobenzonitrile (0.11 g, 0.46 mmol), NaO*t*Bu (0.04 g, 0.46 mmol) and $[PdCl_2(dppf)]$ (5.7 mg, 7.75 μmol, 2.5 mol%) in DMSO (0.62 mL) at 40 ºC under CO_2 atmosphere (0.5-1.0 atm) afforded**,** after purification by flash column chromatography (silica gel, hexane/EtOAc, 80:20), 85 mg (90%) of the titled compound as a pale yellow oil. ^{1}**H-NMR** (400 MHz, $CDCl_3$): δ = 7.62-7.58 (m, 2H), 7.39-7.29 (m, 3H), 7.28-7.22 (m, 4H), 4.47 (s, 2H), 4.06 (q, *J* = 2.0 Hz, 2H), 2.10 (t, *J* = 2.1 Hz, 3H). ^{13}**C-NMR** (100 MHz, $CDCl_3$): δ 155.4 (s), 143.9 (s), 140.6 (s), 134.9 (s), 132.6 (d), 129.1 (d), 128.4 (d), 128.2 (d), 128.0 (d), 118.7 (s), 111.1 (s), 111.0 (s), 48.1 (t), 47.5 (t), 16.1 (q). **IR** (neat):

ν 3031, 2917, 2870, 2224, 1773, 1682, 1605, 1473, 1421, 1309, 1255, 1221, 1053, 957, 904, 837, 751, 733, 701, 673, 579, 532 cm^{-1}. **HRMS (ESI)** *m/z* calcd for $C_{19}H_{16}N_2O_2Na$ [M+Na$^+$] 327.1104, found 327.1103.

(*E*)-3-Benzyl-5-[1-(3-bromophenyl)ethylidene]oxazolidin-2-one (3e)

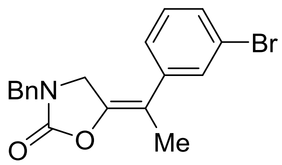

Conditions A: Following the general procedure described above for the carboxylative cyclization and cross-coupling reaction of propargylamines and aryl iodides, the reaction of *N*-benzylbut-2-yn-1-amine (**1**) (0.05 g, 0.31 mmol), methyl 3-bromoiodobenzene (0.13 g, 0.46 mmol), NaO*t*Bu (0.04 g, 0.46 mmol) and [PdCl$_2$(dppf)] (5.7 mg, 7.75 μmol, 2.5 mol%) in DMSO (0.62 mL) at 40 ºC under CO_2 atmosphere (0.5-1.0 atm) afforded**,** after purification by flash column chromatography (silica gel, hexane/EtOAc, 85:15), 98 mg (87%) of the titled compound as pale yellow oil. **^{1}H-NMR** (400 MHz, $CDCl_3$): δ 7.38-7.22 (m, 7H), 7.17 (t, *J* = 7.8 Hz, 1H), 7.08-7.02 (m, 1H), 4.45 (s, 2H), 4.02 (q, *J* = 2.1 Hz, 2H), 2.05 (t, *J* = 2.2 Hz, 3H). **^{13}C-NMR** (100 MHz, $CDCl_3$): δ 155.7 (s), 141.2 (s), 139.5 (s), 135.2 (s), 130.5 (d), 130.3 (d, 2 x CH), 129.1 (d), 128.3 (d), 128.2 (d), 126.0 (d), 122.9 (s), 111.2 (s), 48.1 (t), 47.4 (t), 16.6 (q). **IR** (neat): ν 2917, 1775, 1692, 1588, 1556, 1473, 1416, 1255, 1218, 1057, 882, 791, 749, 702 cm^{-1}. **HRMS (ESI)** *m/z* calcd for $C_{18}H_{16}BrNO_2Na$ [M+Na$^+$] 380.0257, found 380.0259.

(*E*)-3-Benzyl-5-[1-(4-methoxyphenyl)ethylidene]oxazolidin-2-one (3f)

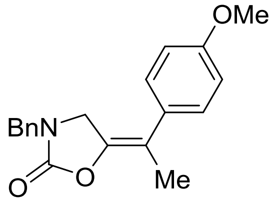

Conditions A: Following the general procedure described above for the carboxylative cyclization and cross-coupling reaction of propargylamines and aryl iodides, the reaction of *N*-benzylbut-2-yn-1-amine (**1**) (0.05 g, 0.31 mmol), 4-iodoanisole (0.11 g, 0.46 mmol), NaO*t*Bu (0.04 g, 0.46 mmol) and [PdCl$_2$(dppf)] (5.7 mg, 7.75 μmol, 2.5 mol%) in DMSO (0.62 mL) at 40 ºC under CO_2 atmosphere (0.5-1.0 atm) afforded**,** after purification by flash column chromatography (silica gel, hexane/EtOAc, 85:15), 98 mg (41%) of the titled compound as pale yellow oil. **^{1}H-NMR** (400 MHz, $CDCl_3$): δ 7.38-7.22 (m, 5H), 7.08-7.04 (m, 2H), 6.87-6.81 (m, 2H), 4.45 (s, 2H), 4.02 (q, *J* = 2.1 Hz, 2H), 3.78 (s, 3H), 2.05 (t, *J* = 2.1 Hz, 3H). **^{13}C-NMR** (100 MHz, $CDCl_3$): δ 158.7 (s), 156.2 (s), 138.1 (s), 135.4 (s), 131.3 (s), 129.1 (d), 128.5 (d), 128.3 (d), 128.2 (d), 114.2 (d), 112.0 (s), 55.4 (q), 48.1 (t), 47.6 (t), 16.7 (q). **IR** (neat): ν 2922, 2838, 1773, 1697, 1608, 1510, 1473, 1420, 1285, 1244, 1179, 1062,

1030, 832, 702 cm^{-1}. **HRMS (ESI)** *m/z* calcd for $C_{19}H_{19}NO_3Na$ [M+Na$^+$] 332.1257, found 332.1257.

(*E*)-3-Benzyl-5-(1-phenylhexylidene)oxazolidin-2-one (4a)

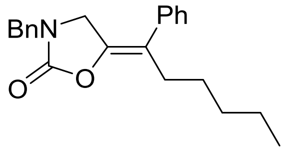

<u>Conditions A</u>: Following the general procedure described above for the carboxylative cyclization and cross-coupling reaction of propargylamines and aryl iodides, the reaction of *N*-benzyloct-2-yn-1-amine (0.05 g, 0.23 mmol), iodobenzene (0.07 g, 0.35 mmol), NaO*t*Bu (0.02 g, 0.25 mmol) and [PdCl$_2$(dppf)] (8.4 mg, 11.5 μmol) in DMSO (0.5 mL) at 40 ºC under CO_2 atmosphere (0.5-1.0 atm) afforded**,** after purification by flash column chromatography (silica gel, hexane/EtOAc, 90:10), 76 mg (> 99%) of the titled compound as pale yellow oil. **^{1}H-NMR** (400 MHz, $CDCl_3$): δ 7.37-7.27 (m, 5H), 7.26-7.21 (m, 3H), 7.14-7.10 (m, 2H), 4.44 (s, 2H), 3.96 (t, *J* = 1.3 Hz, 2H), 2.57-2.48 (m, 2H), 1.37-1.22 (m, 6H), 0.87-0.81 (m, 3H). **^{13}C-NMR** (100 MHz, $CDCl_3$): δ 156.2 (s), 138.4 (s), 138.0 (s), 135.3 (s), 129.0 (d), 128.8 (d), 128.2 (d, 2 x CH), 128.1 (d), 127.3 (d), 117.6 (s), 48.1 (t), 47.3 (t), 31.6 (t), 30.6 (t), 27.5 (t), 22.5 (t), 14.1 (q). **IR** (neat): ν 2953, 2926, 2859, 1775, 1696, 1474, 1419, 1326, 1251, 1219, 1081, 1058, 921, 752, 700, 679 cm^{-1}. **HRMS (ESI)** *m/z* calcd for $C_{22}H_{25}NO_2Na$ [M+Na$^+$] 358.1775, found 358.1778.

(*E*)-3-Benzyl-5-(1-phenyl-2-methylpropylidene)oxazolidin-2-one (5a)

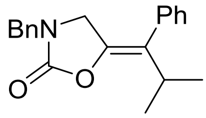

<u>Conditions A</u>: Following the general procedure described above for the carboxylative cyclization and cross-coupling reaction of propargylamines and aryl iodides, the reaction of *N*-benzyl-3-(*iso*-propyl)prop-2-yn-1-amine (0.05 g, 0.27 mmol), iodobenzene (0.08 g, 0.40 mmol), NaO*t*Bu (0.03 g, 0.30 mmol) and [PdCl$_2$(dppf)] (9.9 mg, 13.5 μmol) in DMSO (0.54 mL) at 40 ºC under CO_2 atmosphere (0.5-1.0 atm) afforded**,** after purification by flash column chromatography (silica gel, hexane/EtOAc, 80:20), 73 mg (90%) of the titled compound as colourless oil. **^{1}H-NMR** (400 MHz, $CDCl_3$): δ 7.40-7.16 (m, 8H), 7.12-6.98 (m, 2H), 4.40 (s, 2H), 3.70 (s, 2H), 3.22 (hept, *J* = 6.9 Hz, 1H), 0.99 (d, *J* = 7.0 Hz, 6H). **^{13}C-NMR** (100 MHz, $CDCl_3$): δ 156.3 (s), 137.5 (s), 136.0 (s), 135.4 (s), 129.7 (d), 129.0 (d), 128.6 (d), 128.2 (d), 128.2 (d), 127.5 (d), 122.5 (s), 48.0 (t), 47.0 (t), 28.7 (d), 21.0 (q, 2 x CH_3). **IR** (neat): ν 2962, 2927, 2870, 1777, 1696, 1419, 1362, 1332, 1301, 1251, 1167, 1080, 1058, 1000, 941, 783, 752, 702, 676, 614, 571 cm^{-1}. **HRMS (ESI)** *m/z* calcd for $C_{20}H_{21}NO_2Na$ [M+Na$^+$] 330.1465, found 330.1464.

(*E*)-3-Benzyl-5-(diphenylmethylene)oxazolidin-2-one (6a)

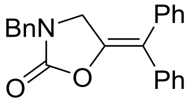

<u>Conditions A</u>: Following the general procedure described above for the carboxylative cyclization and cross-coupling reaction of propargylamines and aryl iodides, the reaction of *N*-benzyl-3-phenylprop-2-yn-1-amine (0.05 g, 0.23 mmol), iodobenzene (0.07 g, 0.35 mmol), NaO*t*Bu (0.02 g, 0.25 mmol) and [$PdCl_2$(dppf)] (8.4 mg, 11.5 μmol) in DMSO (0.5 mL) at 40 ºC under CO_2 atmosphere (0.5-1.0 atm) afforded**,** after purification by flash column chromatography (silica gel, hexane/EtOAc, 80:20), 61 mg (79%) of the titled compound as colourless oil. **^{1}H-NMR** (400 MHz, $CDCl_3$): δ 7.42-7.18 (m, 13H), 7.16-7.12 (m, 2H), 4.47 (s, 2H), 4.03 (s, 2H). **^{13}C-NMR** (100 MHz, $CDCl_3$): δ 155.8 (s), 139.8 (s), 137.9 (s), 136.9 (s), 135.1 (s), 129.8 (d), 129.2 (d), 129.1 (d, 2 x CH), 128.3 (d), 128.22 (d), 128.20 (d), 127.8 (d), 127.2 (d), 116.9 (s), 48.4 (t), 48.0 (t). **IR** (neat): ν 3057, 3031, 1777, 1666, 1495, 1470, 1418, 1260, 1080, 1051, 959, 767, 749, 697, 678, 640 cm^{-1}. **HRMS (ESI)** *m/z* calcd for $C_{23}H_{19}NO_2Na$ [M+Na$^+$] 364.1308, found 364.1304.

(*E*)-3-Benzyl-5-benzylideneoxazolidin-2-one (7a)

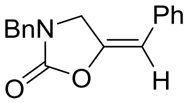

<u>Conditions A</u>: Following the general procedure described above for the carboxylative cyclization and cross-coupling reaction of propargylamines and aryl iodides, the reaction of *N*-benzylprop-2-yn-1-amine (14) (0.05 g, 0.34 mmol), iodobenzene (0.11 g, 0.52 mmol), NaO*t*Bu (0.04 g, 0.37 mmol) and [$PdCl_2$(dppf)] (12.4 mg, 17.0 μmol) in DMSO (0.68 mL) at 40 ºC under CO_2 atmosphere (0.5-1.0 atm) afforded**,** after purification by flash column chromatography (silica gel, hexane/EtOAc, 85:15), 69 mg (76%) of the titled compound as colourless solid. **M.p.** 130-132 °C. **^{1}H-NMR** (400 MHz, $CDCl_3$): δ 7.42-7.27 (m, 7H), 7.19 (t, *J* = 7.4 Hz, 1H), 7.06-7.02 (m, 2H), 6.29 (t, *J* = 2.6 Hz, 1H), 4.54 (s, 2H), 4.32 (d, *J* = 2.7 Hz, 2H). **^{13}C-NMR** (100 MHz, $CDCl_3$): δ 155.3 (s), 144.3 (s), 135.1 (s), 133.7 (s), 129.1 (d), 128.8 (d), 128.3 (d), 128.1 (d), 127.3 (d), 126.7 (d), 105.5 (d), 48.2 (t), 47.9 (t). **IR** (neat): ν 1767, 1685, 1494, 1469, 1423, 1366, 1242, 1224, 1081, 1063, 910, 749, 699, 674, 514 cm^{-1}. **HRMS (ESI)** *m/z* calcd for $C_{17}H_{15}NO_2Na$ [M+Na$^+$] 288.0995, found 288.0995.

(*E*)-3-Butyl-5-(1-phenylethylidene)-oxazolidin-2-one (8a)

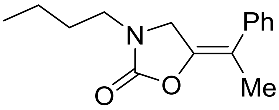

Conditions A: Following the general procedure described above for the carboxylative cyclization and cross-coupling reaction of propargylamines and aryl iodides, the reaction of *N*-butylbut-2-yn-1-amine (0.05 g, 0.40 mmol), iodobenzene (0.12 g, 0.60 mmol), NaO*t*Bu (0.04 g, 0.44 mmol) and [PdCl$_2$(dppf)] (14.6 mg, 20.0 μmol) in DMSO (0.80 mL) at 40 ºC under CO_2 atmosphere (0.5-1.0 atm) afforded**,** after purification by flash column chromatography (silica gel, hexane/EtOAc, 85:15), 77 mg (78%) of the titled compound an orange oil. **^{1}H-NMR** (400 MHz, $CDCl_3$): δ 7.38-7.32 (m, 2H), 7.25 (tt, *J* = 6.6, 1.3 Hz, 1H), 7.22-7.18 (m, 2H), 4.15 (q, *J* = 2.1 Hz, 2H), 3.30-3.24 (m, 2H), 2.08 (t, *J* = 2.2 Hz, 3H), 1.54-1.45 (m, 2H), 1.38-1.25 (m, 2H), 0.91 (t, *J* = 7.3 Hz, 3H). **^{13}C-NMR** (100 MHz, $CDCl_3$): δ 155.8 (s), 139.2 (s), 138.9 (s), 128.8 (d), 127.4 (d), 127.2 (d), 111.9 (s), 48.0 (t), 43.7 (t), 29.3 (t), 19.9 (t), 16.5 (t), 13.7 (q). **IR** (neat): ν 2958, 2865, 1773, 1691, 1474, 1442, 1421, 1280, 1255, 1216, 1112, 1063, 1048, 1005, 755, 700 cm^{-1}. **HRMS (ESI)** *m/z* calcd for $C_{15}H_{19}NO_2Na$ [M+Na$^+$] 268.1308, found 268.1307.

(*E*)-3-*iso*-Propyl-5-(1-phenylethylidene)-oxazolidin-2-one (9a)

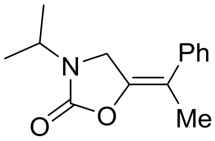

Conditions A: Following the general procedure described above for the carboxylative cyclization and cross-coupling reaction of propargylamines and aryl iodides, the reaction of *N*-(*iso*-propyl)but-2-yn-1-amine (0.04 g, 0.22 mmol), iodobenzene (65 mg, 0.32 mmol), NaO*t*Bu (0.03 g, 0.32 mmol) and [PdCl$_2$(dppf)] (8.0 mg, 11.0 μmol) in DMSO (0.44 mL) at 40 ºC under CO_2 atmosphere (0.5-1.0 atm) afforded**,** after purification by flash column chromatography (silica gel, hexane/EtOAc, 85:15), 65 mg (78%) of the titled compound as a pale yellow oil. **^{1}H-NMR** (400 MHz, $CDCl_3$): δ 7.39-7.34 (m, 2H), 7.27 (tt, *J* = 6.2, 1.3 Hz, 1H), 7.23-7.19 (m, 2H), 4.21-4.11 (m, 1H), 4.10 (q, *J* = 2.2 Hz, 2H), 2.08 (t, *J* = 2.2 Hz, 3H), 1.15 (d, *J* = 6.8 Hz, 6H). **^{13}C-NMR** (100 MHz, $CDCl_3$): δ 155.0 (s), 139.3 (s), 139.1 (s), 128.8 (d), 127.4 (d), 127.2 (d), 111.9 (s), 44.8 (t), 43.2 (d), 19.8 (q, 2 x CH_3), 16.5 (q). **IR** (neat): ν 2974, 2932, 2874, 1769, 1691, 1473, 1418, 1364, 1249, 1201, 1164, 1074, 1035, 903, 807, 757, 700, 660 cm^{-1}. **HRMS (ESI)** *m/z* calcd for $C_{14}H_{17}NO_2Na$ [M+Na$^+$] 254.1152, found 254.1150.

(*E*)-3-Allyl-5-(1-phenylethylidene)-oxazolidin-2-one (10a)

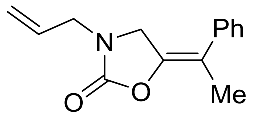

Conditions A: Following the general procedure described above for the carboxylative cyclization and cross-coupling reaction of propargylamines and aryl iodides, the reaction of *N*-allylbut-2-yn-1-amine (0.05 g, 0.46 mmol), iodobenzene (0.14 g, 0.69 mmol), NaO*t*Bu (0.05 g, 0.51 mmol) and [$PdCl_2$(dppf)] (16.8 mg, 23.0 μmol) in DMSO (0.92 mL) at 40 ºC under CO_2 atmosphere (0.5-1.0 atm) afforded**,** after purification by flash column chromatography (silica gel, hexane/EtOAc, 80:20), 76 mg (72%) of the titled compound as a colourless oil. **^{1}H-NMR** (400 MHz, $CDCl_3$): δ 7.38-7.32 (m, 2H), 7.29-7.24 (m, 1H), 7.22-7.17 (m, 2H), 5.80-5.68 (m, 1H), 5.26-5.23 (m, 1H), 5.22-5.19 (m, 1H), 4.12 (q, *J* = 2.1 Hz, 2H), 3.90 (t, *J* = 1.4 Hz, 1H), 3.88 (t, *J* = 1.3 Hz, 1H), 2.09 (t, *J* = 2.2 Hz, 3H). **^{13}C-NMR** (100 MHz, $CDCl_3$): δ 155.7 (s), 139.2 (s), 138.8 (s), 131.5 (d), 128.8 (d), 127.4 (d), 127.3 (d), 119.3 (t), 112.4 (s), 47.7 (t), 46.7 (t), 16.6 (q). **IR** (neat): ν 2917, 2859, 1777, 1693, 1475, 1417, 1313, 1254, 1220, 1064, 1026, 943, 918, 770, 757, 702 cm^{-1}. **HRMS (ESI)** *m/z* calcd for $C_{14}H_{15}NO_2Na$ [M+Na^+] 252.0995, found 252.0990.

(*E*)-3-*tert*-Butyl-5-(1-phenylethylidene)-oxazolidin-2-one (11a)

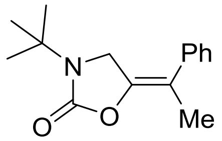

Conditions A: Following the general procedure described above for the carboxylative cyclization and cross-coupling reaction of propargylamines and aryl iodides, the reaction of *N*-(*tert*-butyl)but-2-yn-1-amine (0.05 g, 0.40 mmol), iodobenzene (0.12 g, 0.60 mmol), NaO*t*Bu (0.04 g, 0.44 mmol) and [$PdCl_2$(dppf)] (14.6 mg, 20.0 μmol) in DMSO (0.8 mL) at 40 ºC under CO_2 atmosphere (0.5-1.0 atm) for 3 days afforded**,** after purification by flash column chromatography (silica gel, hexane/EtOAc, 90:10), 36 mg (38%) of the titled compound as a pale yellow solid. **M.p.** 91-93 °C. **^{1}H-NMR** (400 MHz, $CDCl_3$): δ 7.39-7.33 (m, 2H), 7.29-7.23 (m, 1H), 7.22-7.18 (m, 2H), 4.22 (q, *J* = 2.2 Hz, 2H), 2.06 (t, *J* = 2.2 Hz, 3H), 1.38 (s, 9H). **^{13}C-NMR** (100 MHz, $CDCl_3$): δ 154.5 (s), 139.5 (s), 138.8 (s), 128.8 (d), 127.5 (d), 127.1 (d), 111.0 (s), 53.9 (s), 46.7 (t), 27.6 (q, 3 x CH_3), 16.4 (q). **IR** (neat): ν 2978, 1750, 1691, 1461, 1445, 1393, 1367, 1270, 1226, 1162, 1065, 1016, 915, 760, 700, 654, 586 cm^{-1}. **HRMS (ESI)** *m/z* calcd for $C_{15}H_{20}NO_2$ [M+H^+] 246.1489, found 246.1489.

(*E*)-3-Benzyl-5-(diphenylmethylene)-4-methyl-oxazolidin-2-one (12a)

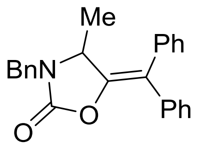

Conditions A: Following the general procedure described above for the carboxylative cyclization and cross-coupling reaction of propargylamines and aryl iodides, the reaction of *N*-benzyl-4-phenylbut-3-yn-2-amine (0.05 g, 0.21 mmol), iodobenzene (65 mg, 0.32 mmol), NaO*t*Bu (0.03 g, 0.32 mmol) and [$PdCl_2$(dppf)] (7.7 mg, 10.5 μmol) in DMSO (0.42 mL) at 40 ºC under CO_2 atmosphere (0.5-1.0 atm) afforded**,** after purification by flash column chromatography (silica gel, hexane/EtOAc, 85:15), 62 mg (81%) of the titled compound as a colourless oil. **^{1}H-NMR** (400 MHz, $CDCl_3$): δ 7.40-7.25 (m, 12H), 7.23-7.18 (m, 1H), 7.17-7.14 (m, 2H), 4.86 (d, *J* = 15.4 Hz, 1H), 4.48 (q, *J* = 6.3 Hz, 1H), 4.12 (d, *J* = 15.4 Hz, 1H), 0.97 (d, *J* = 6.4 Hz, 3H). **^{13}C-NMR** (100 MHz, $CDCl_3$): δ 155.2 (s), 145.5 (s), 138.0 (s), 137.5 (s), 135.5 (s), 130.3 (d), 129.3 (d), 129.1 (d), 129.0 (d), 128.2 (d), 128.1 (d), 128.0 (d), 127.8 (d), 127.2 (d), 117.4 (s), 53.9 (t), 45.4 (t), 17.0 (q). **IR** (neat): ν 1777, 1663, 1494, 1443, 1413, 1251, 1083, 1074, 1029, 999, 769, 735, 699 cm^{-1}. **HRMS (ESI)** *m/z* calcd for $C_{24}H_{21}NO_2Na$ [$M+Na^+$] 378.1465, found 378.1460.

(*E*)-5-Benzylideneoxazolidin-2-one (13a)

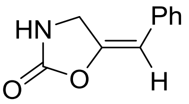

Conditions A: Following the general procedure described above for the carboxylative cyclization and cross-coupling reaction of propargylamines and aryl iodides, the reaction of propargylamine (0.04 g, 0.72 mmol), iodobenzene (0.22 g, 1.08 mmol), NaO*t*Bu (0.10 g, 1.08 mmol) and [$PdCl_2$(dppf)] (26.3 mg, 36.0 μmol) in DMSO (1.44 mL) at 40 ºC under CO_2 atmosphere (0.5-1.0 atm) afforded**,** after purification by flash column chromatography (silica gel, hexane/CH_2Cl_2/EtOAc, 50:20:30), 65 mg (51%) of the titled compound as a colourless solid. **M.p.** 162-164 °C. **^{1}H-NMR** (400 MHz, $CDCl_3$): δ 7.39-7.32 (m, 2H), 7.27-7.21 (m, 1H), 7.12-7.08 (m, 2H), 6.32 (s, 1H), 5.84 (br. s, 1H), 4.56 (d, *J* = 2.5 Hz, 2H). **^{13}C-NMR** (100 MHz, $CDCl_3$): δ 156.8 (s), 146.6 (s), 133.8 (s), 129.0 (d), 127.5 (d), 126.9 (d), 105.5 (d), 45.0 (t). **IR** (neat): ν 3250, 3167, 2361, 2162, 2013, 1775, 1682, 1381, 1241, 1218, 1124, 971, 754, 687, 529, 421 cm^{-1}. **HRMS (ESI)** *m/z* calcd for $C_{10}H_9NO_2Na$ [$M+Na^+$] 198.0526, found 198.0524.

5.2 Sonogashira-carboxylative cyclization and cross-coupling reaction of propargylamines and aryl iodides

3-Benzyl-5-(diphenylmethylene)oxazolidin-2-one (6a)

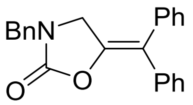

<u>Conditions B</u>: Following the general procedure described above for the Sonogashira-carboxylative cyclization and cross-coupling reaction of propargylamines and aryl iodides, the reaction of *N*-benzylprop-2-yn-1-amine **(14)** (0.04 g, 0.28 mmol), iodobenzene (0.17 g, 0.83 mmol), DABCO (0.08 g, 0.73 mmol), CuI (2.7 mg, 14.0 μmol) and [PdCl$_2$(dppf)] (10.2 mg, 14.0 μmol) in DMSO (2.8 mL) at 60 ºC under CO_2 atmosphere (0.5-1.0 atm) afforded**,** after purification by flash column chromatography (silica gel, hexane/EtOAc, 85:15), 67 mg (71%) of the titled compound as a pale yellow oil.

3-Benzyl-5-(diphenylmethylene)-4-methyl-oxazolidin-2-one (12a)

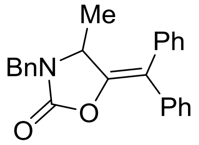

<u>Conditions B</u>: Following the general procedure described above for the Sonogashira-carboxylative cyclization and cross-coupling reaction of propargylamines and aryl iodides, the reaction of *N*-benzylbut-3-yn-2-amine (0.05 g, 0.31 mmol), iodobenzene (0.19 g, 0.94 mmol), DABCO (0.09 g, 0.81 mmol), CuI (3.0 mg, 15.5 μmol) and [PdCl$_2$(dppf)] (11.3 mg, 15.5 μmol) in DMSO (3.1 mL) at 60 ºC under CO_2 atmosphere (0.5-1.0 atm) afforded**,** after purification by flash column chromatography (silica gel, hexane/EtOAc, 85:15), 61 mg (55%, calculated by ^{1}H-NMR with 1,2-dibromoethane as internal standard) of the titled compound as a colourless oil.

5-(Diphenylmethylene)-3-(4-methoxybenzyl)oxazolidin-2-one (15)

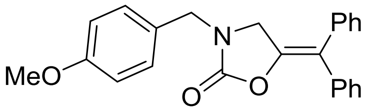

<u>Conditions B</u>: Following the general procedure described above for the Sonogashira-carboxylative cyclization and cross-coupling reaction of propargylamines and aryl iodides, the reaction of *N*-(4-methoxybenzyl)prop-2-yn-1-amine (0.23 g, 1.34 mmol), iodobenzene (0.82 g, 4.02 mmol), DABCO (0.39 g, 3.48 mmol), CuI (12.7 mg, 67.0 μmol) and [PdCl$_2$(dppf)] (49.0 mg, 67.0 μmol) in DMSO (13 mL) at 60 ºC under CO_2 atmosphere (0.5-1.0 atm) afforded**,** after purification by flash column chromatography (silica gel, hexane/EtOAc, 90:10), 359 mg (72%) of the titled compound as a yellow solid (purity > 95%).

M. p. 135-137 °C. **^{1}H-NMR** (400 MHz, $CDCl_3$): δ 7.41-7.12 (m, 12H), 6.87 (d, J = 8.7 Hz, 2H), 4.41 (s, 2H), 4.01 (s, 2H), 3.80 (s, 3H). **^{13}C-NMR** (100 MHz, $CDCl_3$): δ 159.7 (s), 155.7 (s), 139.9 (s), 138.0 (s), 136.9 (s), 129.9 (d), 129.7 (d), 129.2 (d), 129.1 (d), 128.2 (d), 127.8 (d), 127.2 (d), 127.2 (s), 116.9 (s), 114.4 (d), 55.4 (q), 48.2 (t), 47.5 (t).

IR (neat): ν 3057, 3026, 2927, 2838, 1781, 1668, 1611, 1513, 1468, 1441, 1417, 1249, 1177, 1054, 959, 788, 767, 700, 677 cm^{-1}. **HRMS (ESI)** *m/z* calcd for $C_{24}H_{22}NO_3$ $[M+H^+]$ 372.1594, found 372.1592.

5-(Diphenylmethylene)-3-*Iso*-propyloxazolidin-2-one (16)

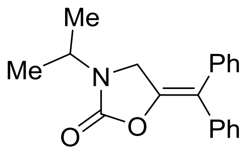

Conditions B: Following the general procedure described above for the Sonogashira-carboxylative cyclization and cross-coupling reaction of propargylamines and aryl iodides, the reaction of *N*-(*iso*-propyl)prop-2-yn-1-amine (0.04 g, 0.41 mmol), iodobenzene (0.25 g, 1.23 mmol), DABCO (0.12 g, 1.07 mmol), CuI (3.9 mg, 20.5 μmol) and $[PdCl_2(dppf)]$ (15.0 mg, 20.5 μmol) in DMSO (4.1 mL) at 60 ºC under CO_2 atmosphere (0.5-1.0 atm) afforded**,** after purification by flash column chromatography (silica gel, hexane/EtOAc, 85:15), 0.06 g (47%) of the titled compound as a pale yellow solid. **M. p.** 142-144 °C. **^{1}H-NMR** (400 MHz, $CDCl_3$): δ 7.46-7.16 (m, 10H), 4.20 (hept, *J* = 6.7 Hz, 1H), 4.07 (s, 2H), 1.17 (d, *J* = 6.8 Hz, 6H). **^{13}C-NMR** (100 MHz, $CDCl_3$): δ 154.9 (s), 140.3 (s), 138.2 (s), 137.0 (s), 130.0 (d), 129.2 (d), 129.1 (d), 128.2 (d), 127.8 (d), 127.1 (d), 116.5 (s), 45.0 (d), 44.2 (t), 19.9 (q, 2 x CH_3). **IR** (neat): ν 3057, 2974, 2932, 2874, 1777, 1666, 1497, 1469, 1444, 1416, 1367, 1251, 1205, 1161, 1072, 1023, 955, 811, 767, 753, 698, 667, 646 cm^{-1}. **HRMS (ESI)** *m/z* calcd for $C_{19}H_{20}NO_2$ $[M+H^+]$ 294.1489, found 294.1487.

3-Benzyl-5-[bis(3-methoxyphenyl)methylene]oxazolidin-2-one (17)

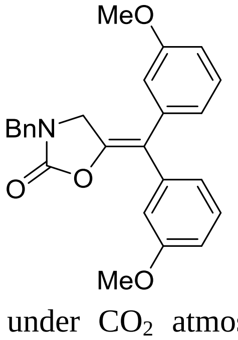

Conditions B: Following the general procedure described above for the Sonogashira-carboxylative cyclization and cross-coupling reaction of propargylamines and aryl iodides, the reaction of *N*-benzylprop-2-yn-1-amine **(14)** (0.05 g, 0.34 mmol), 3-iodoanisole (0.24 g, 1.03 mmol), DABCO (0.10 g, 0.88 mmol), CuI (3.2 mg, 17.0 μmol) and $[PdCl_2(dppf)]$ (12.4 mg, 17.0 μmol) in DMSO (3.4 mL) at 60 ºC under CO_2 atmosphere (0.5-1.0 atm) afforded**,** after purification by flash column chromatography (silica gel, hexane/EtOAc, 85:15), 0.09 g (68%, calculated by ^{1}H-NMR with 1,2-dibromoethane as internal standard) of the titled compound as a pale yellow oil.

^{1}H-NMR (400 MHz, $CDCl_3$): δ 7.38-7.29 (m, 3H), 7.27-7.22 (m, 3H), 7.19 (d, *J* = 8.0 Hz, 1H), 7.02-6.98 (m, 1H), 6.93-6.90 (m, 1H), 6.82 (ddd, *J* = 8.3, 2.6, 0.9 Hz, 1H), 6.76 (ddd, *J* = 8.3, 2.6, 0.9 Hz, 1H), 6.72 (ddd, *J* = 7.5, 1.5, 1.0 Hz, 1H), 6.66 (dd, *J* =

2.5, 1.6 Hz, 1H), 4.45 (s, 2H), 4.02 (s, 2H), 3.74 (s, 3H), 3.73 (s, 3H). **^{13}C-NMR** (100 MHz, $CDCl_3$): δ 160.1 (s), 159.4 (s), 155.7 (s), 140.1 (s), 139.1 (s), 138.0 (s), 135.1 (s), 130.1 (d), 129.14 (d), 129.08 (d), 128.3 (d), 128.2 (d, 2), 122.1 (d), 121.7 (d), 116.6 (s), 115.5 (d), 115.0 (d), 113.2 (d), 112.6 (d), 55.3 (q), 48.4 (t), 48.0 (t). **IR** (neat): ν 2937, 2833, 1778, 1668, 1597, 1578, 1487, 1466, 1418, 1320, 1284, 1259, 1209, 1159, 1081, 1046, 982, 956, 856, 780, 749, 700, 677 cm^{-1}. **HRMS (ESI)** *m/z* calcd for $C_{25}H_{24}NO_4$ [M+H$^+$] 402.1700, found 402.1695.

3-Benzyl-5-[bis(3-(methoxymethoxy)phenyl)methylene]oxazolidin-2-one (18)

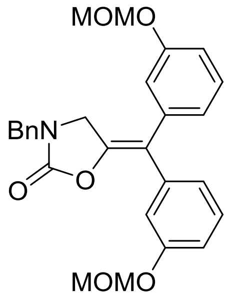

<u>Conditions B</u>: Following the general procedure described above for the Sonogashira-carboxylative cyclization and cross-coupling reaction of propargylamines and aryl iodides, the reaction of *N*-benzylprop-2-yn-1-amine **(14)** (0.08 g, 0.55 mmol), 1-iodo-3-(methoxymethoxy)benzene (0.44 g, 1.65 mmol), DABCO (0.16 g, 1.43 mmol), CuI (5.2 mg, 27.5 μmol) and [$PdCl_2$(dppf)] (20.1 mg, 27.5 μmol) in DMSO (5.5 mL) at 60 ºC under CO_2 atmosphere (0.5-1.0 atm) afforded**,** after purification by flash column chromatography (silica gel, hexane/EtOAc, 80:20), 0.16 g (62%) of the titled compound as a colourless oil. **^{1}H-NMR** (400 MHz, $CDCl_3$): δ 7.39-7.29 (m, 3H), 7.28-7.18 (m, 4H), 7.08-7.02 (m, 2H), 6.98 (ddd, *J* = 8.3, 2.5, 0.9 Hz, 1H), 6.93 (ddd, *J* = 8.2, 2.3, 1.1 Hz, 1H), 6.83-6.80 (m, 1H), 6.80-6.76 (m, 1H), 5.13 (s, 2H), 5.12 (s, 2H), 4.47 (s, 2H), 4.04 (s, 2H), 3.46 (s, 3H), 3.43 (s, 3H). **^{13}C-NMR** (100 MHz, $CDCl_3$): δ 157.8 (s), 157.1 (s), 155.7 (s), 140.2 (s), 139.1 (s), 138.1 (s), 135.1 (s), 130.1 (d), 129.2 (d), 129.1 (d, 2), 128.3 (d), 128.2 (d), 123.3 (d), 123.0 (d), 117.8 (d), 117.5 (d), 116.4 (s), 115.5 (d), 114.8 (d), 94.7 (t), 94.6 (t), 56.2 (q), 56.1 (q), 48.3 (t), 48.0 (t). **IR** (neat): ν 2953, 2903, 2828, 1782, 1668, 1599, 1579, 1486, 1419 , 1314 , 1263, 1244, 1150, 1079, 1053, 1022, 972, 922, 879, 857, 789, 750, 701, 677 cm^{-1}. **HRMS (ESI)** *m/z* calcd for $C_{27}H_{28}NO_6$ [M+H$^+$] 462.1911, found 462.1915.

3-Benzyl-5-[bis(3-(bencyloxy)phenyl)methylene]oxazolidin-2-one (19)

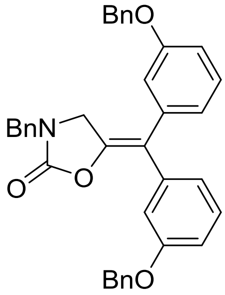

<u>Conditions B</u>: Following the general procedure described above for the Sonogashira-carboxylative cyclization and cross-coupling reaction of propargylamines and aryl iodides, the reaction of *N*-benzylprop-2-yn-1-amine **(14)** (0.25 g, 1.72 mmol), 3-(benzyloxy)-1-iodo-benzene (1.6 g, 5.16 mmol), DABCO (0.5 g, 4.47 mmol),

CuI (16.4 mg, 86.0 μmol) and [$PdCl_2$(dppf)] (63.0 mg, 86.0 μmol) in DMSO (17 mL) at 60 °C under CO_2 atmosphere (0.5-1.0 atm) afforded**,** after purification by flash column chromatography (silica gel, hexane/EtOAc, 90:10), 602 mg (63%) of the titled compound as a pale yellow oil. **^{1}H-NMR** (400 MHz, $CDCl_3$): δ 7.43-7.19 (m, 15H), 7.08-7.04 (m, 1H), 6.99-6.97 (m, 1H), 6.99-6.97 (m, 1H), 6.93 (ddd, *J* = 8.3, 2.5, 1.0 Hz, 1H), 6.93 (ddd, *J* = 8.3, 2.5, 1.0 Hz, 1H), 6.86 (ddd, *J* = 8.2, 2.6, 0.8 Hz, 1H), 6.75-6.71 (m, 2H), 5.01 (s, 4H), 4.46 (s, 2H), 3.96 (s, 2H). **^{13}C-NMR** (100 MHz, $CDCl_3$): δ 159.2 (s), 158.7 (s), 155.7 (s), 140.1 (s), 139.1 (s), 138.0 (s), 137.1 (s), 136.8 (s), 135.2 (s), 130.2 (d), 129.2 (d), 129.1 (d), 129.1 (d), 128.7 (d), 128.7 (d), 128.3 (d), 128.2 (d), 128.0 (d), 127.8 (d), 127.6 (d), 122.4 (d), 122.0 (d), 116.6 (s), 116.3 (d), 115.8 (d), 114.5 (d), 113.7 (d), 70.1 (t, 2 x CH_2), 48.3 (t), 48.0 (t). **IR** (neat): ν 3031, 1783, 1670, 1577, 1488, 1420, 1319, 1262, 1192, 1158, 1081, 1049, 874, 786, 740, 699 cm^{-1}. **HRMS (ESI)** *m/z* calcd for $C_{37}H_{32}NO_4$ [$M+H^+$] 554.2326, found 554.2327.

3-Benzyl-5-(bis-m-tolylmethylene)oxazolidin-2-one (20)

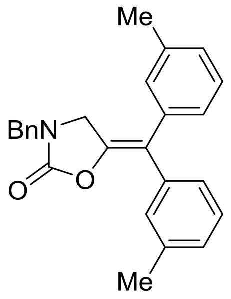

<u>Conditions B</u>: Following the general procedure described above for the Sonogashira-carboxylative cyclization and cross-coupling reaction of propargylamines and aryl iodides, the reaction of *N*-benzylprop-2-yn-1-amine **(14)** (0.05 g, 0.34 mmol), 3-iodotoluene (0.23 g, 1.03 mmol), DABCO (0.10 g, 0.88 mmol), CuI (3.2 mg, 17.0 μmol) and [$PdCl_2$(dppf)] (12.4 mg, 17.0 μmol) in DMSO (3.4 mL) at 60 °C under CO_2 atmosphere (0.5-1.0 atm) afforded**,** after purification by flash column chromatography (silica gel, hexane/EtOAc, 85:15), 86 mg (68%) of the titled compound as a pale yellow oil. **^{1}H-NMR** (400 MHz, $CDCl_3$): δ 7.40-7.15 (m, 9H), 7.14-7.09 (m, 1H), 7.07-7.02 (m, 1H), 6.98-6.94 (m, 2H), 4.48 (s, 2H), 4.03 (s, 2H), 2.31 (s, 6H). **^{13}C-NMR** (100 MHz, $CDCl_3$): δ 156.0 (s), 139.5 (s), 138.8 (s), 138.0 (s), 137.7 (s), 136.9 (s), 135.3 (s), 130.4 (d), 129.7 (d), 129.1 (d), 128.9 (d), 128.5 (d), 128.3 (d), 128.2 (d), 128.1 (d), 128.0 (d), 126.8 (d), 126.4 (d), 117.1 (s), 48.4 (t), 48.0 (t), 21.7 (q), 21.5 (q). **IR** (neat): ν 3034, 2922, 2859, 1780, 1739, 1666, 1602, 1418, 1264, 1082, 1055, 957, 790, 749, 702, 678 cm^{-1}. **HRMS (ESI)** *m/z* calcd for $C_{25}H_{24}NO_2$ [$M+H^+$] 370.1802, found 370.1794.

3-Benzyl-5-[bis-(4-bromo-3-methoxyphenyl)methylene]oxazolidin-2-one (21)

<u>Conditions B</u>: Following the general procedure described above for the Sonogashira-carboxylative cyclization and cross-coupling reaction of propargylamines and aryl

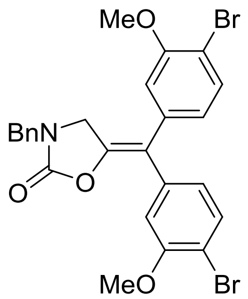

iodides, the reaction of *N*-benzylprop-2-yn-1-amine **(14)** (0.04 g, 0.28 mmol), 2-bromo-5-iodoanisole (0.26 g, 0.83 mmol), DABCO (0.08 g, 0.73 mmol), CuI (2.7 mg, 14.0 μmol) and [$PdCl_2$(dppf)] (10.2 mg, 14.0 μmol) in DMSO (2.8 mL) at 60 ºC under CO_2 atmosphere (0.5-1.0 atm) afforded**,** after purification by flash column chromatography (silica gel, hexane/EtOAc, 85:15), 87 mg (56%, calculated by ^{1}H-NMR with 1,2-dibromoethane as internal standard) of the titled compound as a colourless solid. **M.p.** 189-191 °C. **^{1}H-NMR** (400 MHz, $CDCl_3$): δ 7.53 (d, *J* = 8.0 Hz, 1H), 7.43 (d, *J* = 8.3 Hz, 1H), 7.40-7.29 (m, 3H), 7.28-7.24 (m, 2H), 7.05 (d, *J* = 2.0 Hz, 1H), 6.74 (dd, *J* = 8.3, 2.0 Hz, 1H), 6.65 (dd, *J* = 8.0, 1.9 Hz, 1H), 6.61 (d, *J* = 1.9 Hz, 1H), 4.47 (s, 2H), 3.99 (s, 2H), 3.83 (s, 3H), 3.78 (s, 3H). **^{13}C-NMR** (100 MHz, $CDCl_3$): δ 156.6 (s), 155.7 (s), 155.3 (s), 140.8 (s), 138.0 (s), 136.9 (s), 134.9 (s), 134.1 (d), 133.0 (d), 129.2 (d), 128.5 (d), 128.4 (d), 123.2 (d), 122.6 (d), 115.3 (s), 113.4 (d), 112.7 (d), 111.7 (s), 110.9 (s), 56.4 (q, 2 x CH_3), 48.4 (t), 48.1 (t). **IR** (neat): ν 2937, 2854, 1783, 1667, 1585, 1567, 1484, 1465, 1420, 1395, 1325, 1260, 1237, 1215, 1170, 1048, 1025, 997, 909, 860, 819, 786, 732, 702, 670 cm^{-1}. **HRMS (ESI)** *m/z* calcd for $C_{25}H_{22}Br_2NO_4$ [$M+H^+$] 557.9910, found 557.9916.

3-Benzyl-5-[bis-(3,5-dimethoxyphenyl)methylene]oxazolidin-2-one (22)

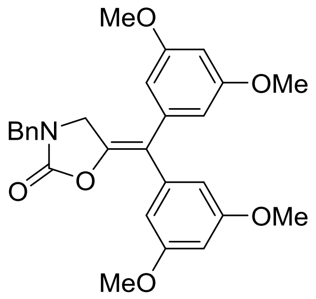

Conditions B: Following the general procedure described above for the Sonogashira-carboxylative cyclization and cross-coupling reaction of propargylamines and aryl iodides, the reaction of *N*-benzylprop-2-yn-1-amine **(14)** (0.25 g, 1.72 mmol), 1-iodo-3,5-dimethoxybenzene (1.36 g, 5.16 mmol), DABCO (0.5 g, 4.47 mmol), CuI (16.4 mg, 86.0 μmol) and [$PdCl_2$(dppf)] (63.0 mg, 86.0 μmol) in DMSO (17 mL) at 60 ºC under CO_2 atmosphere (0.5-1.0 atm) afforded**,** after purification by flash column chromatography (silica gel, hexane/EtOAc, 90:10), 534 mg (67%) of the titled compound as an orange oil (purity > 95%).. **^{1}H-NM**R (400 MHz, $CDCl_3$): δ 7.38-7.28 (m, 3H), 7.28-7.24 (m, 2H), 6.59 (d, *J* = 2.3 Hz, 2H), 6.39 (t, *J* = 2.3 Hz, 1H), 6.36 (t, *J* = 2.3 Hz, 1H), 6.29 (d, *J* = 2.3 Hz, 2H), 4.46 (s, 2H), 4.04 (s, 2H), 3.75 (s, 6H), 3.72 (s, 6H). **^{13}C-NMR** (100 MHz, $CDCl_3$): δ 161.2 (s), 160.5 (s), 155.6 (s), 140.2 (s), 139.6 (s), 138.3 (s), 135.1 (s), 129.1 (d), 128.3 (d), 128.2 (d), 116.6 (s), 107.8 (d), 107.5 (d), 99.7 (s), 99.4 (s), 55.4 (q, 4 x CH_3), 48.4 (t), 48.0 (t). **IR** (neat): ν 2937, 2838, 1779, 1669, 1587, 1454, 1419, 1351,

1325, 1295, 1274, 1201, 1152, 1047, 1009, 989, 925, 838, 728, 701, 672, 542 cm^{-1}. **HRMS (ESI)** *m/z* calcd for $C_{27}H_{28}NO_6$ [M+H$^+$] 462.1911, found 462.1910.

3-Benzyl-5-[bis-(3,4,5-trimethoxyphenyl)methylene]oxazolidin-2-one (23)

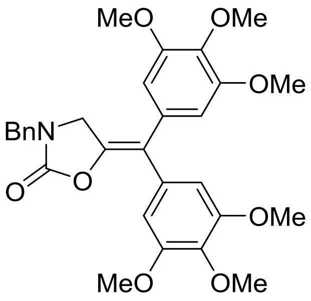

Conditions B: Following the general procedure described above for the Sonogashira-carboxylative cyclization and cross-coupling reaction of propargylamines and aryl iodides, the reaction of *N*-benzylprop-2-yn-1-amine **(14)** (0.04 g, 0.28 mmol), 5-iodo-1,2,3-trimethoxybenzene (0.24 g, 0.83 mmol), DABCO (0.08 g, 0.73 mmol), CuI (2.7 mg, 14.0 μmol) and [$PdCl_2$(dppf)] (10.2 mg, 14.0 μmol) in DMSO (2.8 mL) at 60 ºC under CO_2 atmosphere (0.5-1.0 atm) afforded**,** after purification by flash column chromatography (silica gel, hexane/EtOAc, 60:40), 98.7 mg (68%) of the titled compound as colourless solid. **M.p**. 61-63 °C. **^{1}H-NMR** (400 MHz, $CDCl_3$): δ 7.41-7.24 (m, 5H), 6.65 (s, 2H), 6.35 (s, 2H), 4.48 (s, 2H), 4.03 (s, 2H), 3.87 (s, 3H), 3.85 (s, 3H), 3.79 (s, 6H), 3.76 (s, 6H). **^{13}C-NMR** (100 MHz, $CDCl_3$): δ 155.6 (s), 153.7 (s), 152.9 (s), 139.6 (s), 137.7 (s), 137.6 (s), 135.1 (s), 133.1 (s), 132.1 (s), 129.1 (d), 128.4 (d), 128.3 (d), 116.8 (s), 107.0 (d), 106.7 (d), 61.1 (q), 61.0 (q), 56.4 (q), 48.5 (t), 48.1 (t). **IR** (neat): ν 2937, 2833, 1781, 1669, 1580, 1503, 1453, 1413, 1356, 1326, 1271, 1235, 1124, 1061, 1005, 875, 840, 729, 704 cm^{-1}**. HRMS (ESI)** *m/z* calcd for $C_{29}H_{32}NO_8$ [M+H$^+$] 522.2122, found 522.2126.

3-Benzyl-5-[bis-(4-methoxyphenyl)methylene]oxazolidin-2-one (24)

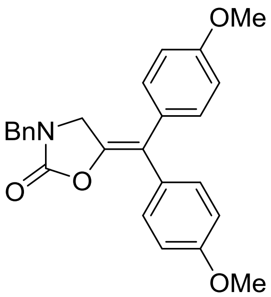

Conditions B: Following the general procedure described above for the Sonogashira-carboxylative cyclization and cross-coupling reaction of propargylamines and aryl iodides, the reaction of *N*-benzylprop-2-yn-1-amine **(14)** (0.05 g, 0.34 mmol), 4-iodoanisole (0.24 g, 1.03 mmol), DABCO (0.10 g, 0.88 mmol), CuI (3.2 mg, 17.0 μmol) and [$PdCl_2$(dppf)] (12.4 mg, 17.0 μmol) in DMSO (0.68 mL, 0.5M) at 60 ºC under CO_2 atmosphere (0.5-1.0 atm) afforded**,** after purification by flash column chromatography (silica gel, hexane/EtOAc, 85:15), 66.3 mg (48%) of the titled compound as a pale yellow oil. **^{1}H-NMR** (400 MHz, $CDCl_3$): δ 7.68-7.62 (m, 2H), 7.14-7.05 (m, 5H), 6.96-6.86 (m, 4H), 6.81-6.75 (m, 2H), 4.18 (s, 2H), 3.70 (s, 2H), 3.42 (s, 3H), 3.39 (s, 3H). **^{13}C-NMR** (100 MHz, $CDCl_3$): δ 159.4 (s), 159.1 (s), 155.7 (s), 139.0 (s), 136.0 (s), 131.3 (d), 130.8 (s), 130.8 (d), 130.5

(s), 129.1 (d), 128.3 (d), 128.1 (d), 115.7 (s), 114.6 (d), 114.0 (d), 54.8 (q), 48.3 (t), 47.9 (t). **IR** (neat): ν 2931, 2837, 1777, 1669, 1606, 1574, 1511, 1467, 1419, 1363, 1247, 1176, 1108, 1081, 1055, 1033, 959, 834, 751, 702, 598, 577 cm^{-1}. **HRMS (ESI)** *m/z* calcd for $C_{25}H_{24}NO_4$ $[M+H^+]$ 402.1700, found 402.1694.

3-Benzyl-5-(bis-(p-tolylmethylene)oxazolidin-2-one (25)

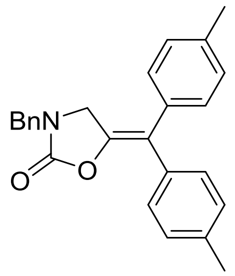

<u>Conditions B</u>: Following the general procedure described above for the Sonogashira-carboxylative cyclization and cross-coupling reaction of propargylamines and aryl iodides, the reaction of *N*-benzylprop-2-yn-1-amine **(14)** (0.25 g, 1.72 mmol), 4-iodotoluene (1.12 g, 5.16 mmol), DABCO (0.5 g, 4.47 mmol), CuI (16.4 mg, 86.0 μmol) and [$PdCl_2$(dppf)] (63.0 mg, 86.0 μmol) in DMSO (17 mL) at 60 ºC under CO_2 atmosphere (0.5-1.0 atm) afforded**,** after purification by flash column chromatography (silica gel, hexane/EtOAc, 90:10), 375 mg (55%) of the titled compound as a pale yellow solid. **M.p.** 147-149 °C. ^{1}**H-NMR** (400 MHz, $CDCl_3$): δ 7.38-7.23 (m, 7H), 7.16-7.12 (m, 2H), 7.12-7.08 (m, 2H), 7.04-7.00 (m, 2H), 4.47 (s, 2H), 4.02 (s, 2H), 2.35 (s, 3H), 2.32 (s, 3H). ^{13}**C-NMR** (100 MHz, $CDCl_3$): δ 156.0 (s), 139.1 (s), 137.5 (s), 136.9 (s), 135.3 (s), 135.1 (s), 134.2 (s), 129.8 (d), 129.7 (d), 129.07 (d), 129.06 (d), 128.9 (d), 128.3 (d), 128.2 (d), 116.8 (s), 48.4 (t), 48.0 (t), 21.3 (q, 2 x CH_3). **IR** (neat): ν 3026, 2922, 2865, 2359, 1782, 1668, 1512, 1471, 1419, 1260, 1054, 959, 822, 750, 702, 673 cm^{-1}. **HRMS (ESI)** *m/z* calcd for $C_{25}H_{24}NO_2$ $[M+H^+]$ 370.1802, found 370.1798.

3-Benzyl-5-(di(thiophen-2-yl)methylene)oxazolidin-2-one (**26**)

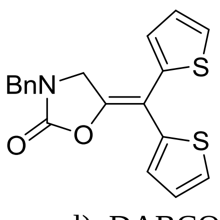

<u>Conditions B</u>: Following the general procedure described above for the Sonogashira-carboxylative cyclization and cross-coupling reaction of propargylamines and aryl iodides, the reaction of *N*-benzylprop-2-yn-1-amine (**14**) (0.05 g, 0.34 mmol), 2-iodothiophene (0.21 g, 1.03 mmol), DABCO (0.10 g, 0.88 mmol), CuI (3.2 mg, 17.0 μmol) and [$PdCl_2$(dppf)] (12.4 mg, 17.0 μmol) in DMSO (3.4 mL) at 60 ºC under CO_2 atmosphere (0.5-1.0 atm) afforded**,** after purification by flash column chromatography (silica gel, hexane/EtOAc, 85:15), 52 mg (42%) of the titled compound as a pale yellow oil. ^{1}**H-NMR** (400 MHz, $CDCl_3$): δ 7.38-7.30 (m, 4H), 7.29 (dd, *J* = 5.2, 1.2 Hz, 1H), 7.28-7.25 (m, 2H), 7.09-7.06 (m, 1H), 7.05 (dd, *J* = 5.2, 3.5 Hz, 1H), 6.99 (dd, *J* = 5.1, 3.7 Hz, 1H), 6.96 (dd, *J* = 3.5, 1.2 Hz, 1H), 4.50 (s, 2H), 4.09 (s, 2H). ^{13}**C-NMR** (100 MHz, $CDCl_3$): δ 155.0 (s),

140.3 (s), 139.5 (s), 137.2 (s), 134.9 (s), 129.1 (d), 128.5 (d), 128.4 (d), 128.2 (d), 127.4 (d), 126.87 (d), 126.85 (d), 125.9 (d), 105.2 (s), 48.2 (t), 48.1 (t). **IR** (neat): ν 3073, 3030, 2921, 2854, 1782, 1665, 1466, 1417, 1327, 1298, 1254, 1228, 1165, 1079, 1044, 954, 934, 847, 746, 699, 674 cm^{-1}. **HRMS (ESI)** *m/z* calcd for $C_{19}H_{16}NO_2S_2$ [M+H$^+$] 354.06170, found 354.06142.

Other products obtained in the optimization of the reaction conditions

(*Z*)-3-Benzyl-5-benzylideneoxazolidin-2-one (*Z*-7a)

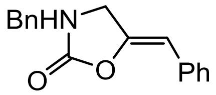

^{1}H-NMR (400 MHz, $CDCl_3$): δ 7.55 (d, *J* = 7.4 Hz, 2H), 7.42 – 7.28 (m, 7H), 7.21 (t, *J* = 7.4 Hz, 1H), 5.45 (t, *J* = 2.0 Hz, 1H), 4.53 (s, 2H), 4.17 (d, *J* = 2.0 Hz, 2H). **^{13}C-NMR** (100 MHz, $CDCl_3$): δ 155.8 (s), 141.9 (s), 135.0 (s), 133.5 (s), 129.2 (d), 128.6 (d), 128.5 (d), 128.38 (d), 128.35 (d), 127.0 (d), 103.3 (d), 48.3 (t), 48.1 (t). **IR** (neat): ν 1774, 1695, 1496, 1473, 1428, 1329, 1305, 1268, 1077, 1052, 956, 926, 834, 749, 698, 667 cm^{-1}. **HRMS (ESI)** *m/z* calcd for $C_{17}H_{15}NNaO_2$ [M+H$^+$] 288.0995, found 288.0994.

3-Benzyl-5-methyleneoxazolidin-2-one (S1)

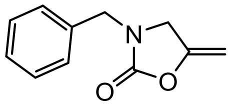

^{1}H-NMR (400 MHz, $CDCl_3$): δ 7.40 – 7.30 (m, 3H), 7.29 – 7.24 (m, 2H), 4.73 (dd, *J* = 5.7, 2.6 Hz, 1H), 4.46 (s, 2H), 4.23 (dt, *J* = 3.1, 2.2 Hz, 1H), 4.03 – 3.99 (m, 2H). **^{13}C-NMR** (100 MHz, $CDCl_3$): δ 155.8 (s), 149.1 (s), 135.1 (s), 129.1 (d), 128.4 (d), 128.3 (d), 86.9 (t), 48.0 (t), 47.4 (t). **IR** (neat): ν 3031, 2927, 1776, 1679, 1472, 1425, 1382, 1364, 1328, 1281, 1237, 1202, 1172, 1082, 1057, 969, 877, 833, 753, 701, 682, 629, 541 cm^{-1}. **HRMS (ESI)** *m/z* calcd for $C_{11}H_{12}NO_2$ [M+H$^+$] 190.0863, found: 190.0863.

6. ^{1}H, ^{13}C and ^{19}F spectra of all new compounds

(*E*)-3-Benzyl-5-(1-phenylethylidene)oxazolidin-2-one (3a)

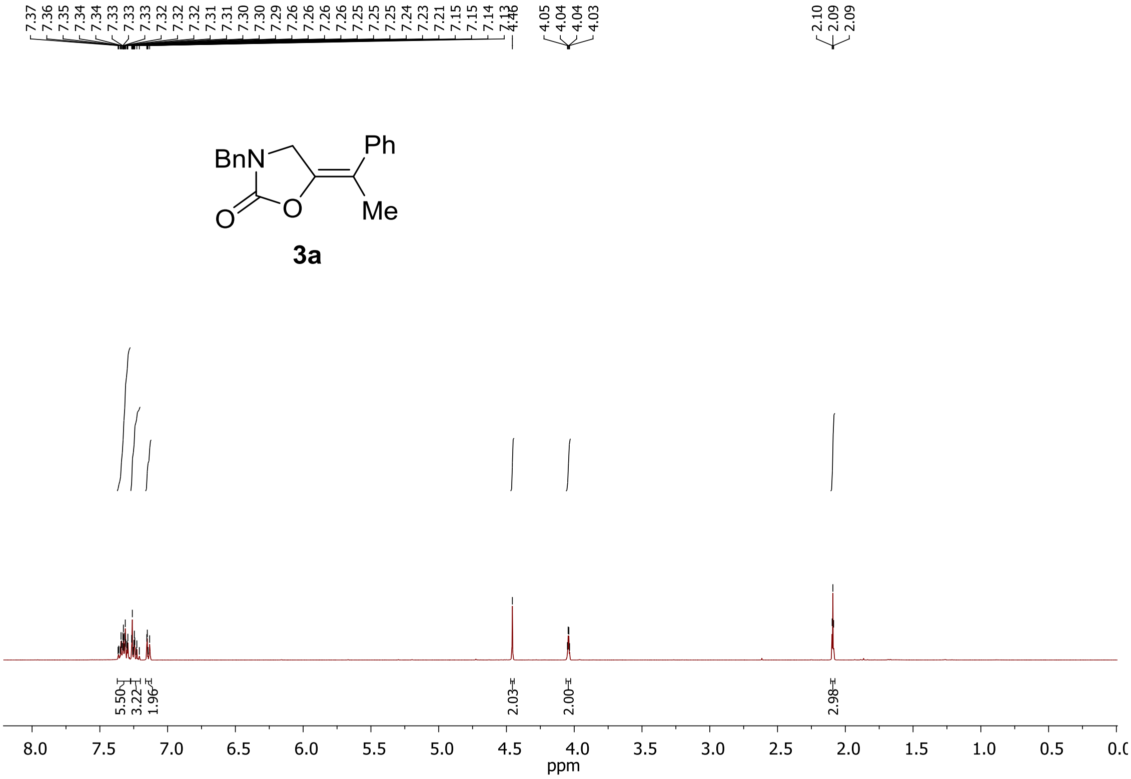

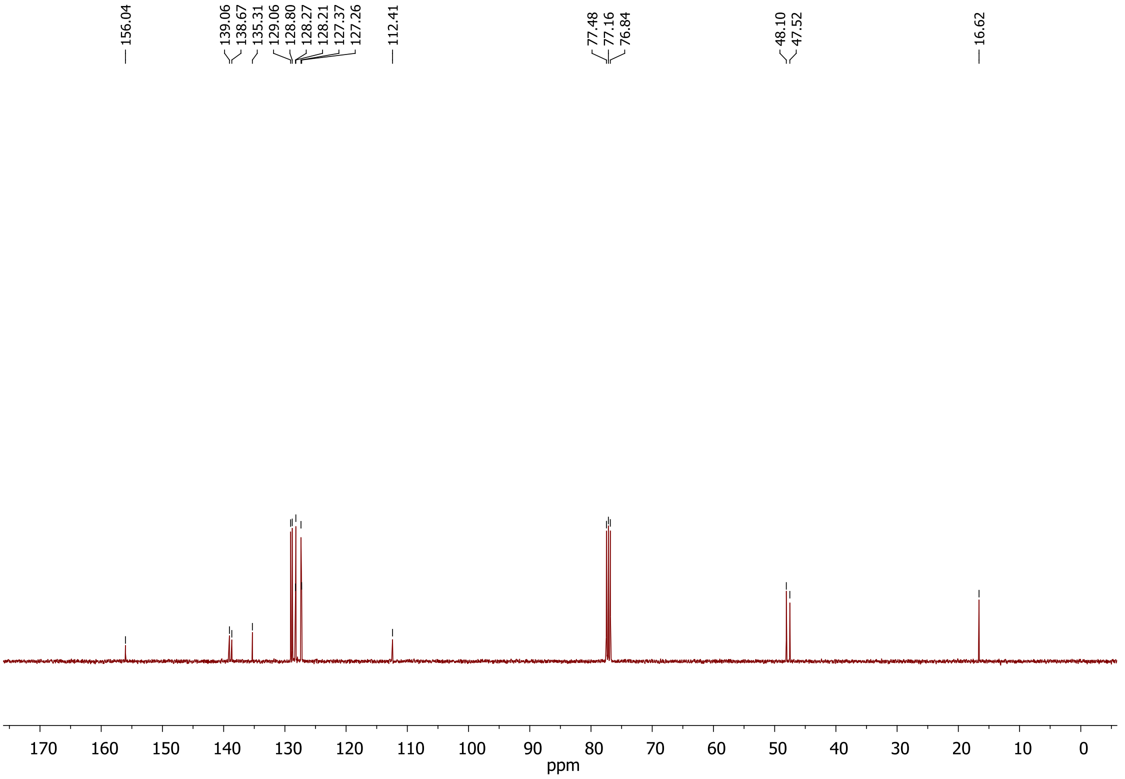

(*E*)-3-Benzyl-5-[1-(4-(trifluromethyl)phenyl)ethylidene]oxazolidin-2-one (3b)

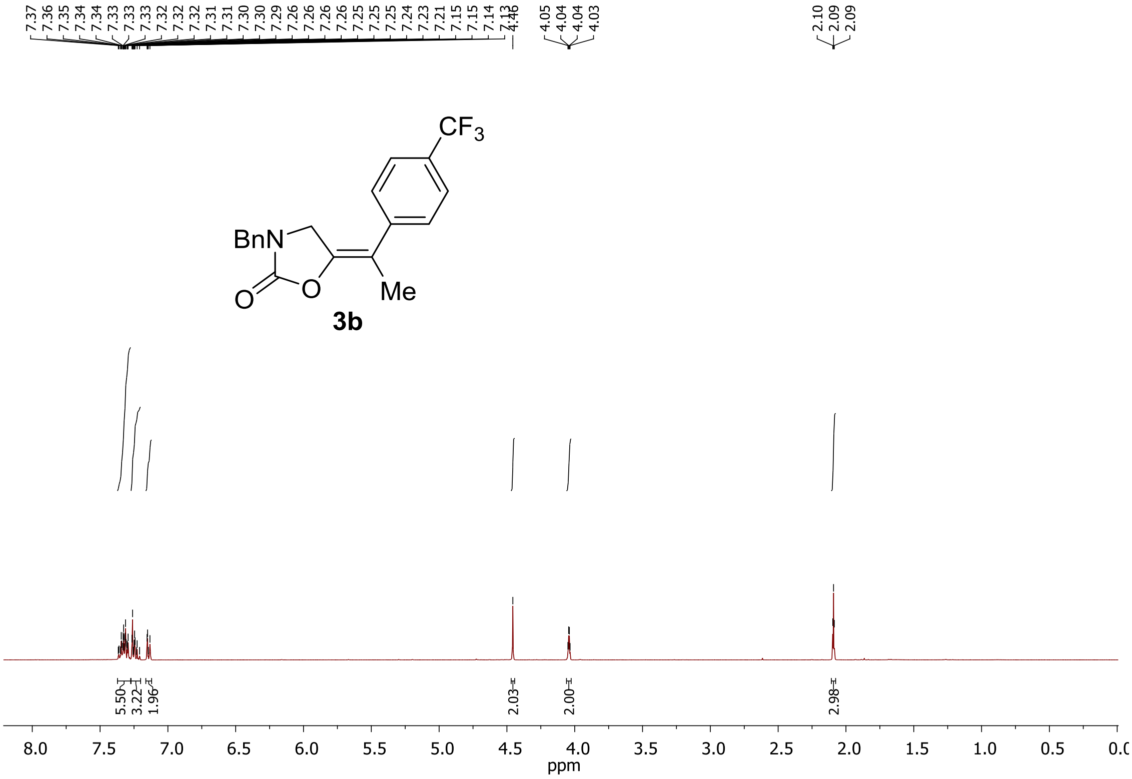

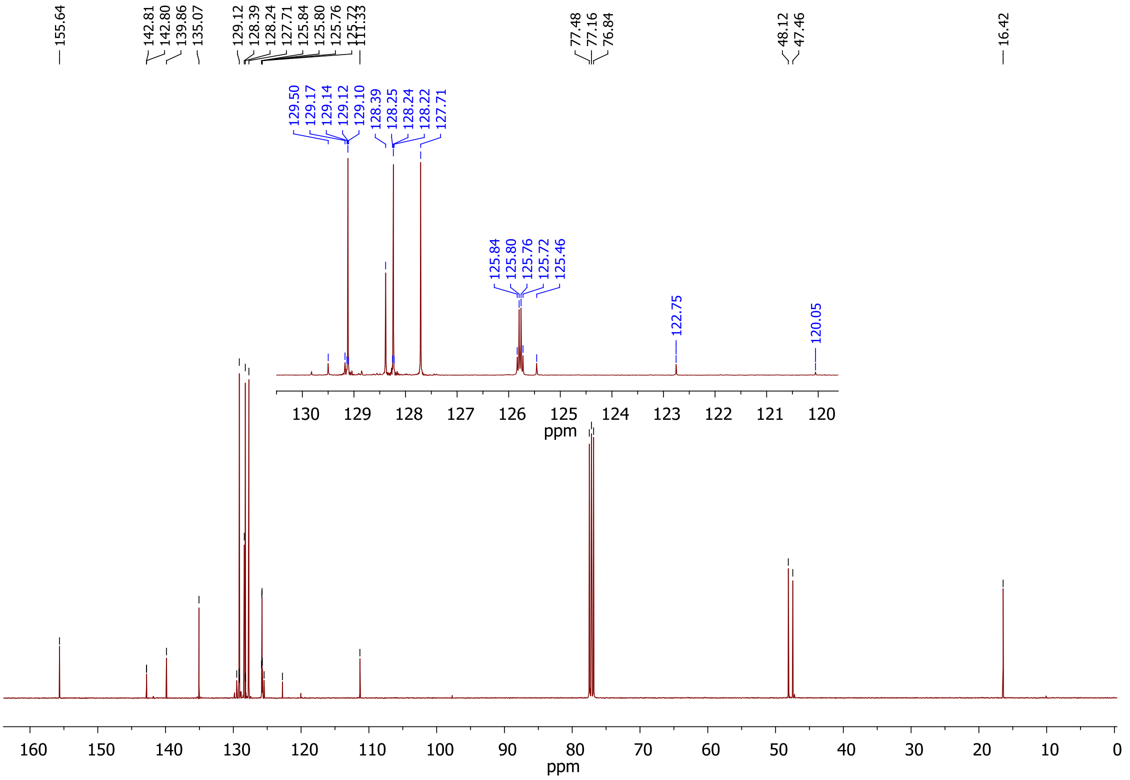

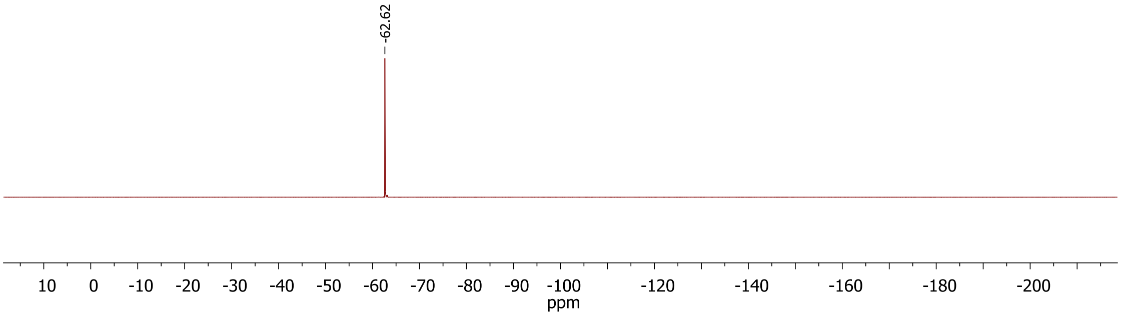
-62.62
10
0
-10
-20
-30
-40
-50
-60
-70
-80
-90
-100
-120
-140
-160
-180
-200
ppm

Methyl (*E*)-4-[1-(3-benzyl-2-oxooxazolidin-5-ylidene)ethyl]benzoate (3c)

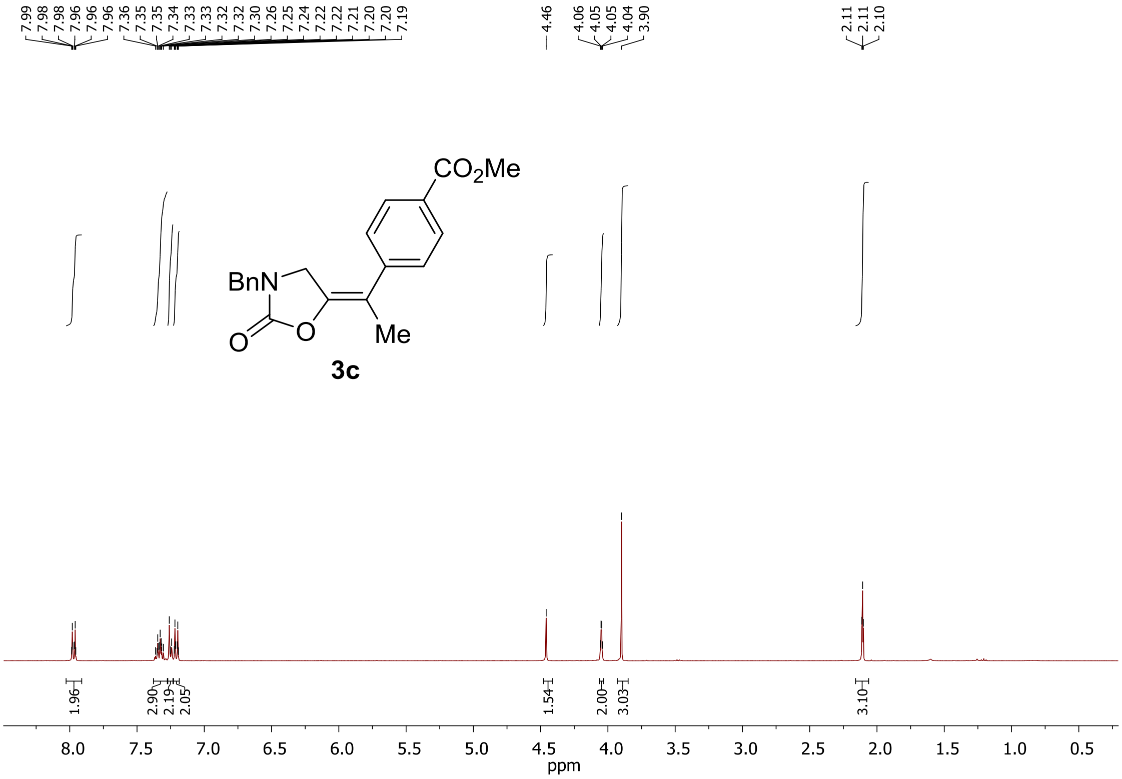

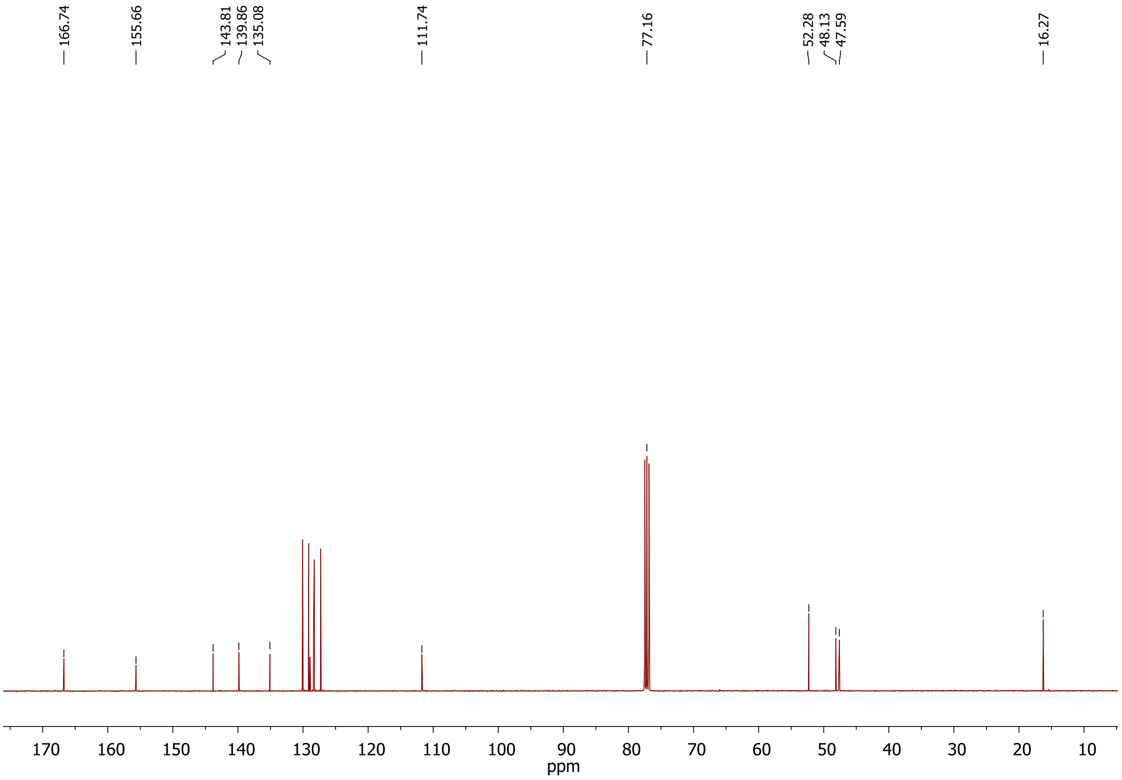

(*E*)-4-[1-(3-benzyl-2-oxooxazolidin-5-ylidene)ethyl]benzonitrile (3d)

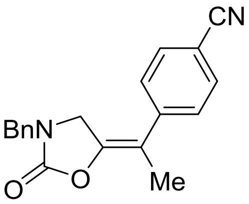

3d

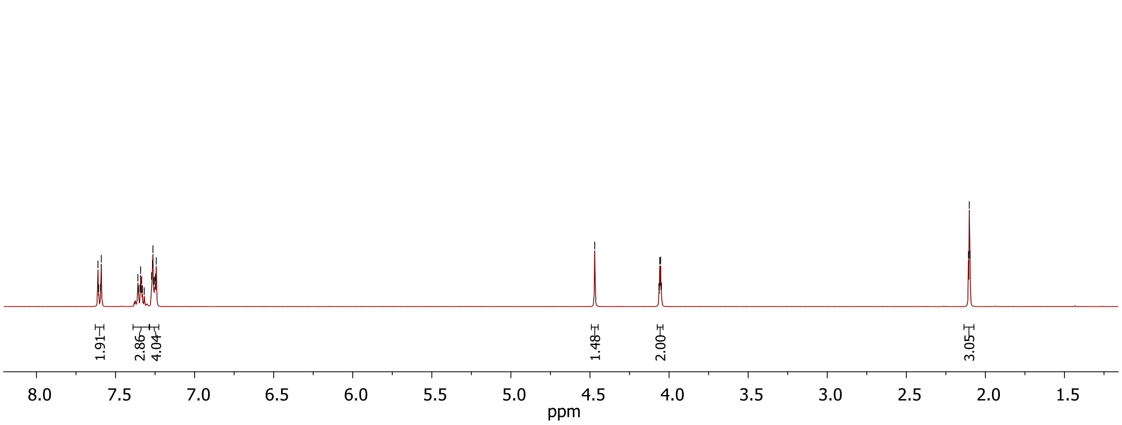

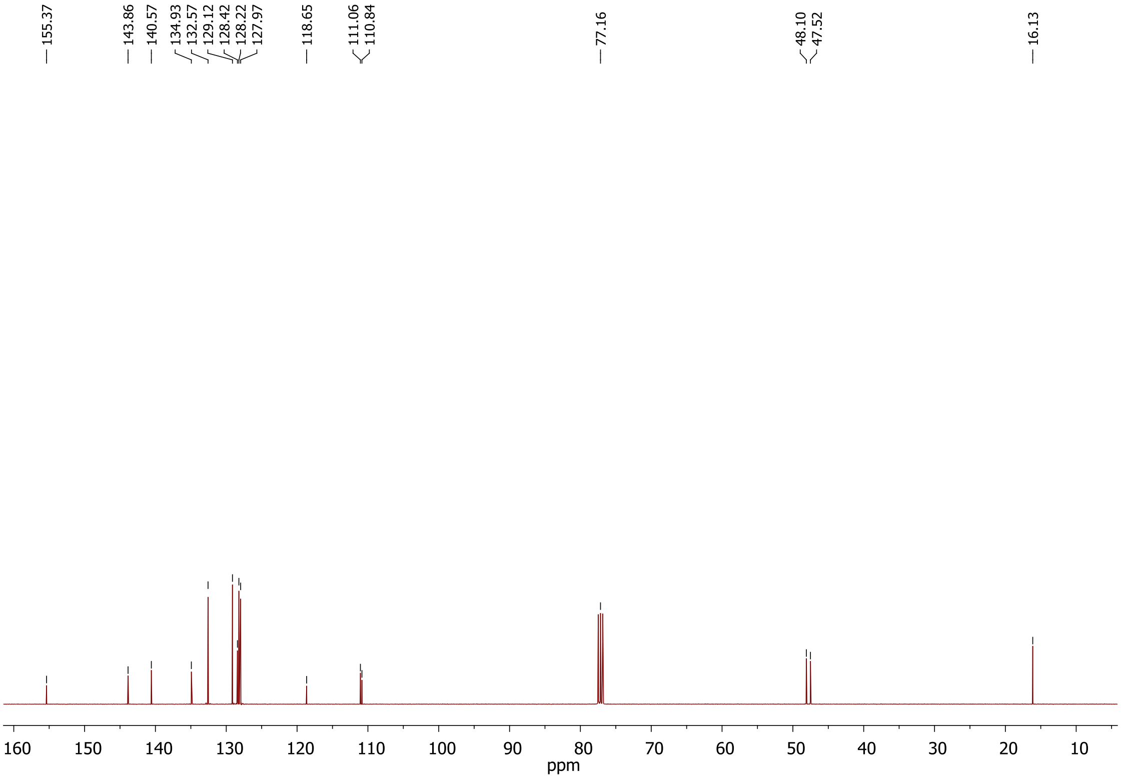

(*E*)-3-Benzyl-5-[1-(3-bromophenyl)ethylidene]oxazolidin-2-one (3e)

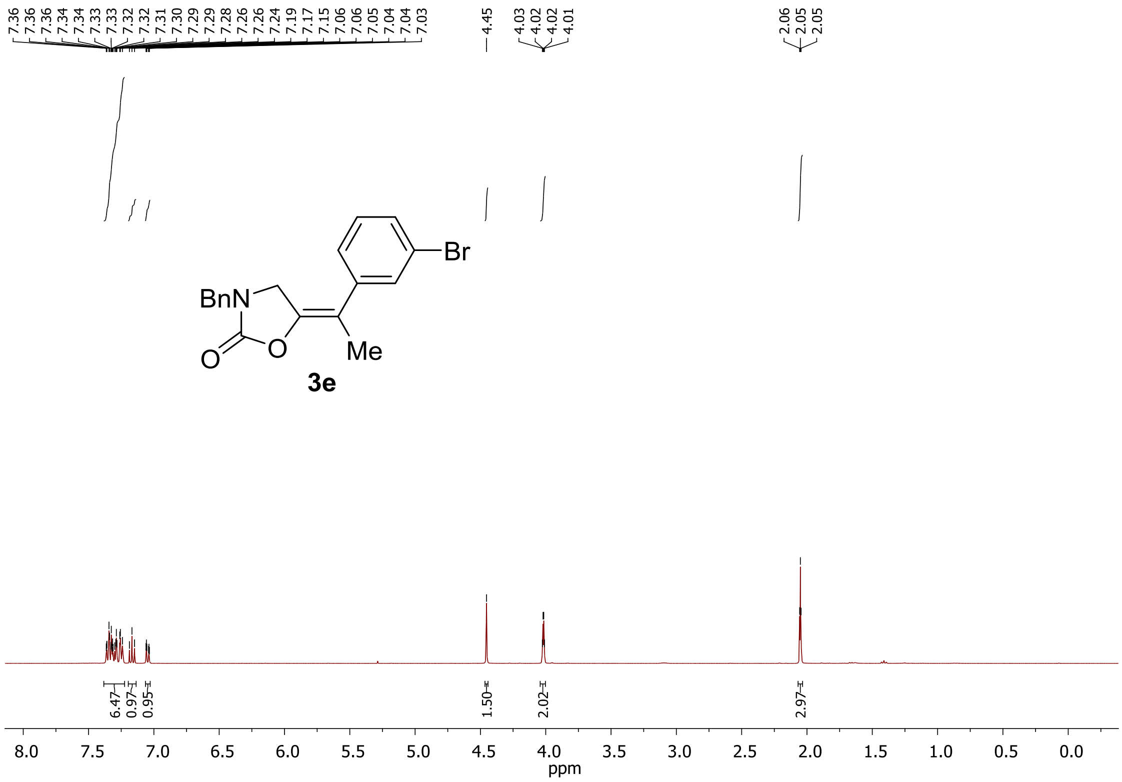

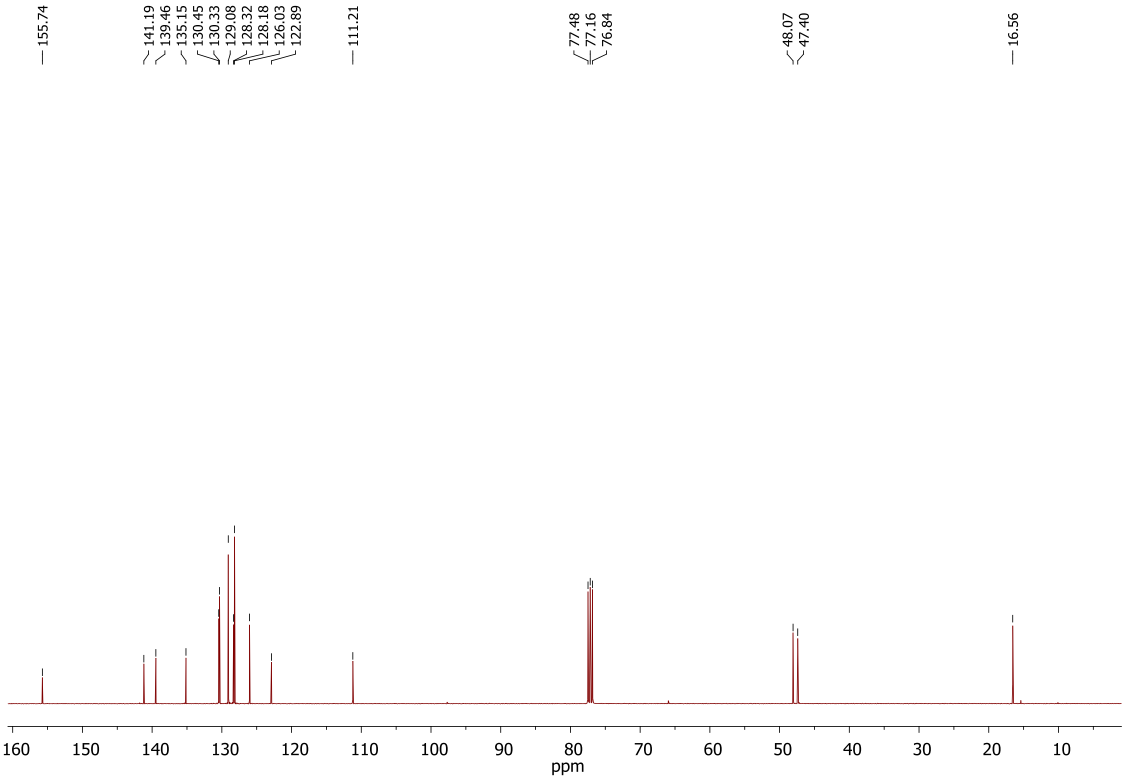

(*E*)-3-Benzyl-5-[1-(4-methoxyphenyl)ethylidene]oxazolidin-2-one (3f)

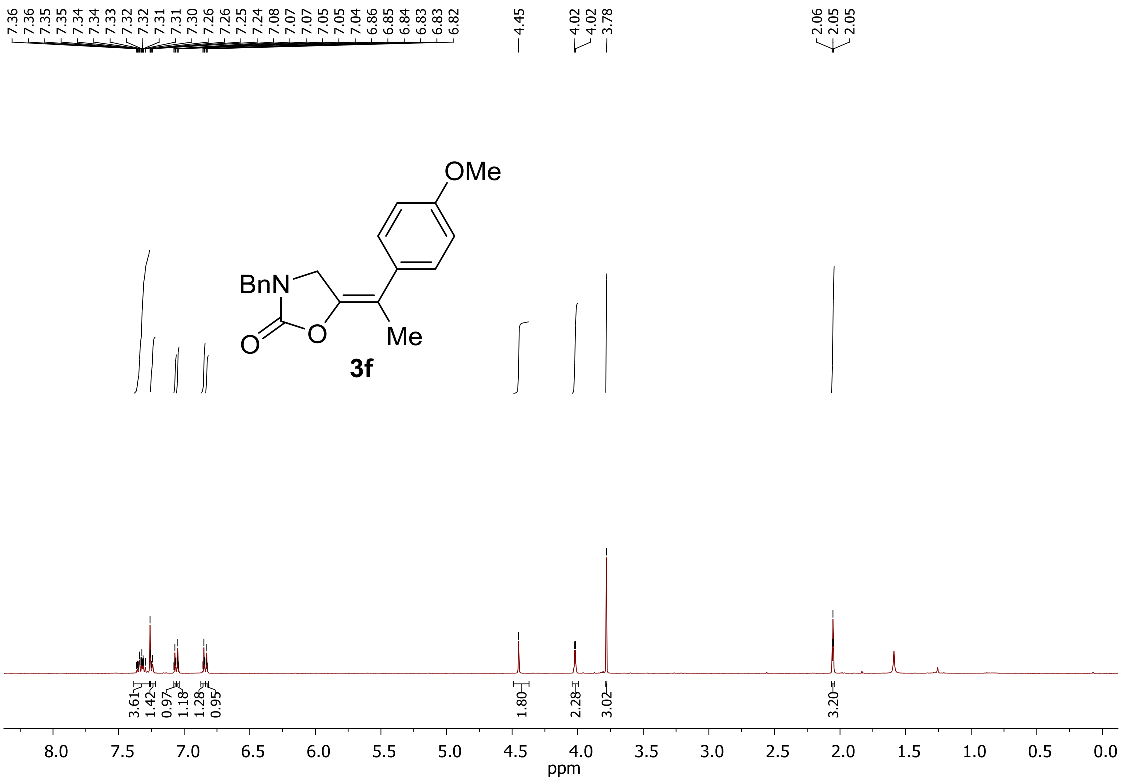

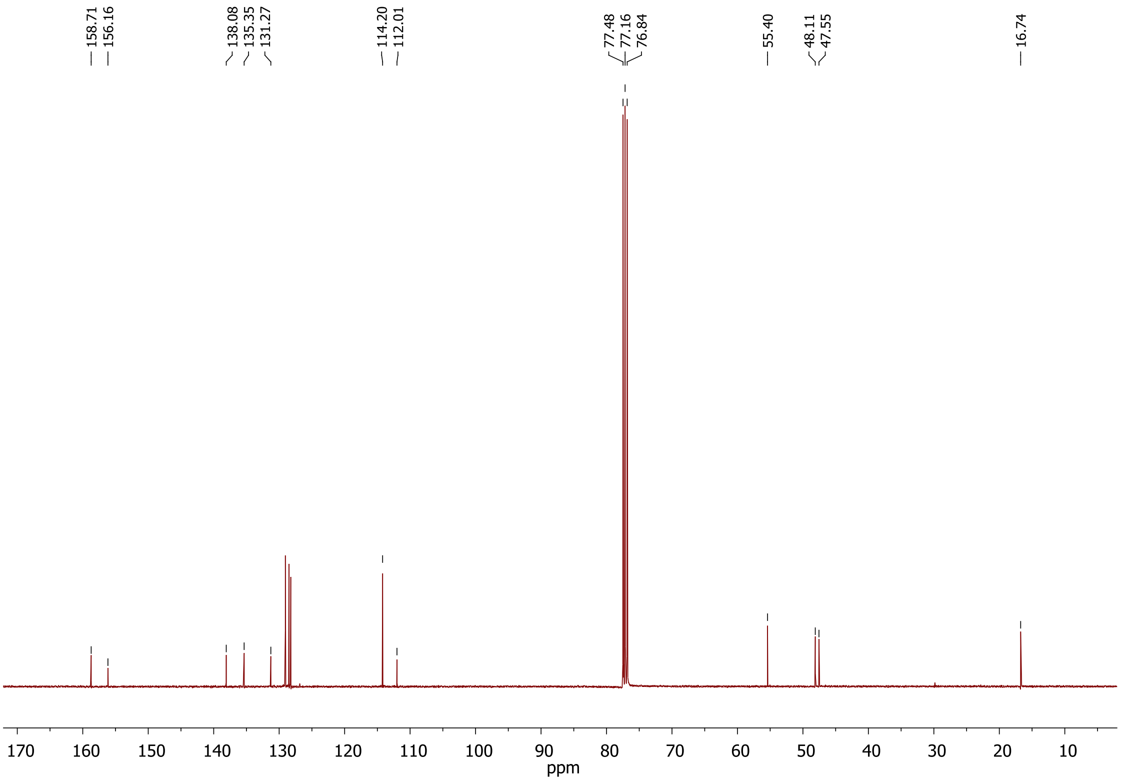

(*E*)-3-Benzyl-5-(1-phenylhexylidene)oxazolidin-2-one (4a)

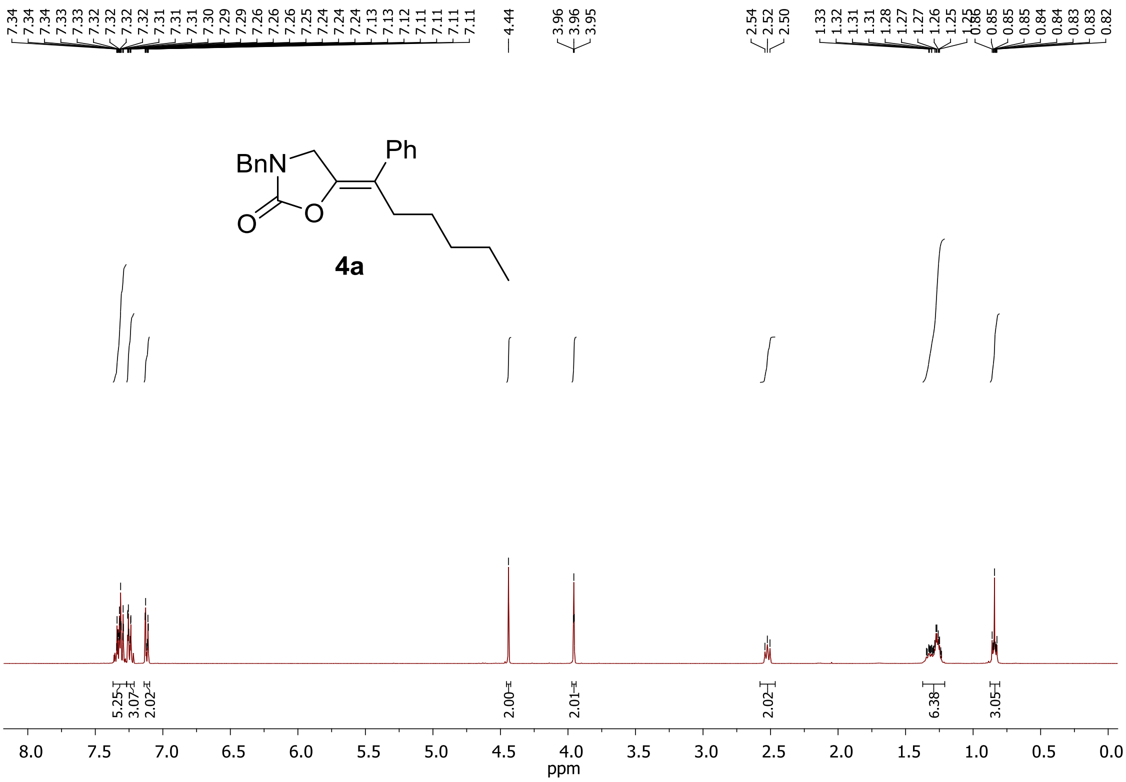

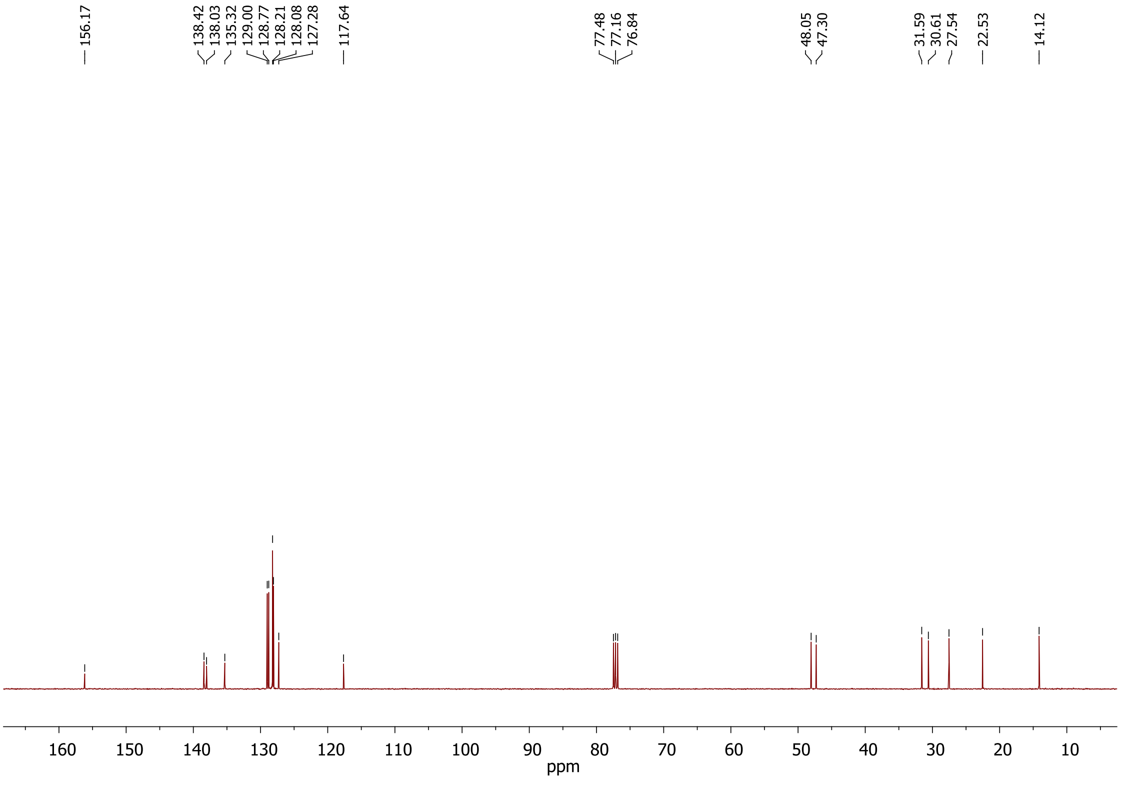

(*E*)-3-Benzyl-5-(1-phenyl-2-methylpropylidene)oxazolidin-2-one (5a)

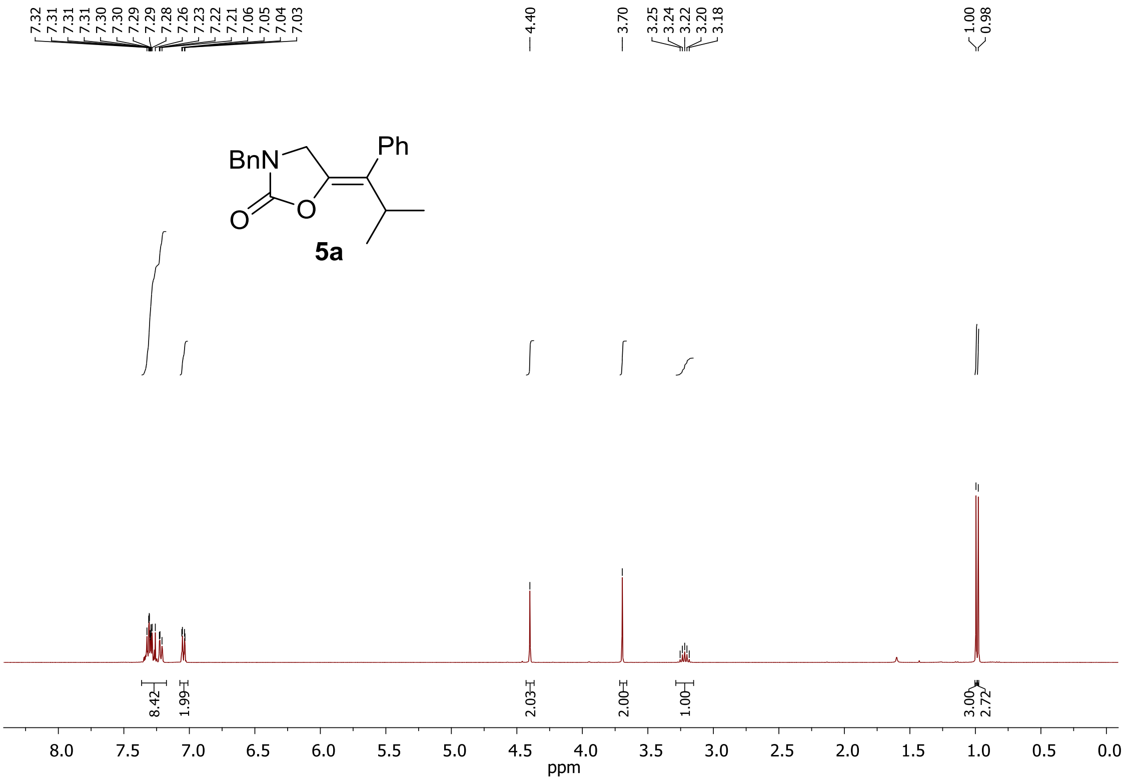

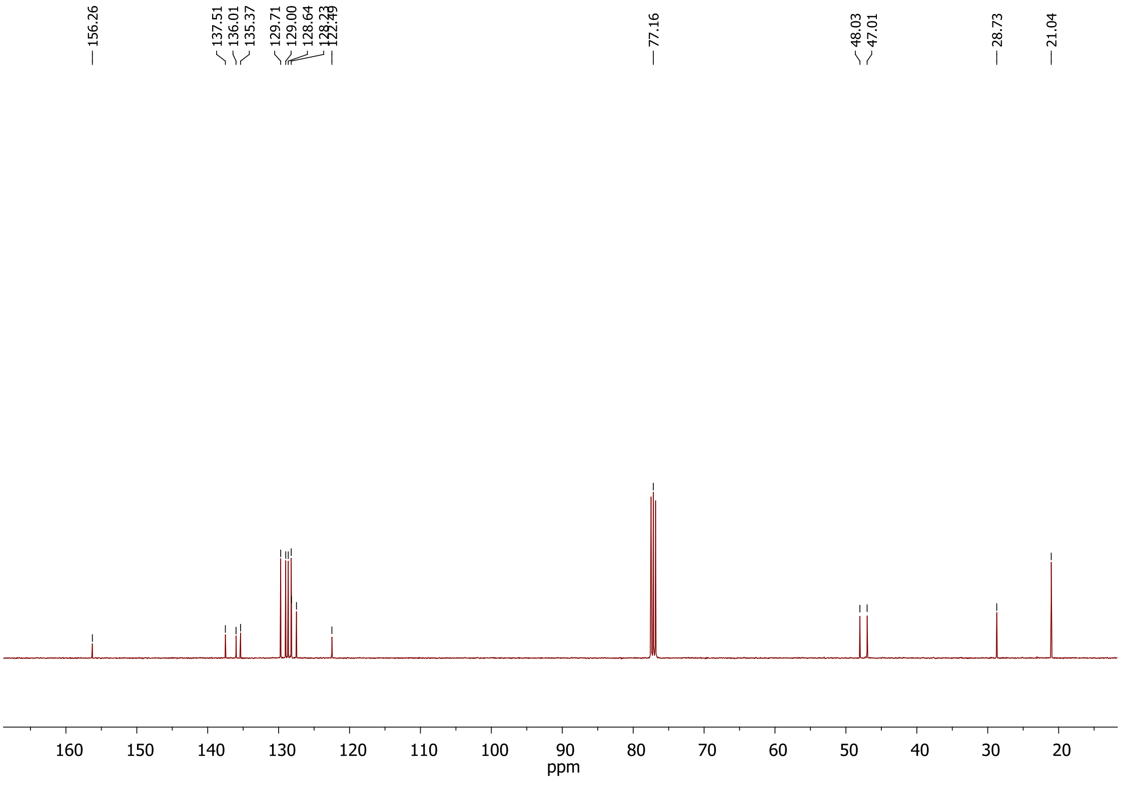

(*E*)-3-Benzyl-5-(diphenylmethylene)oxazolidin-2-one (6a)

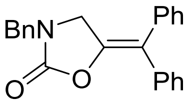

6a

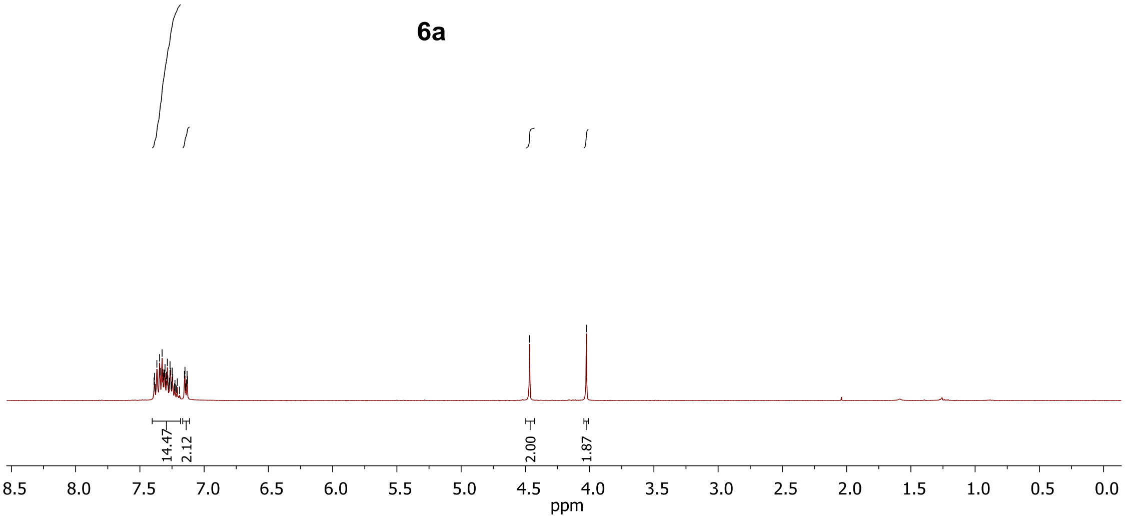

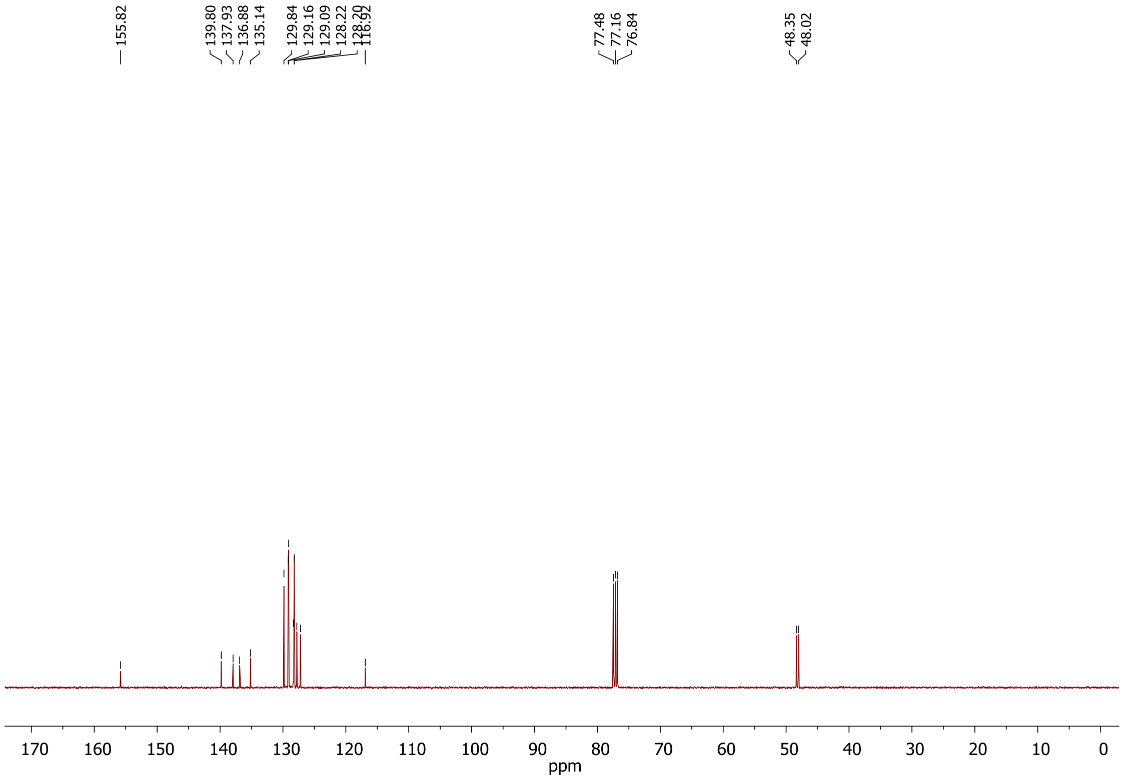

(*E*)-3-Benzyl-5-benzylideneoxazolidin-2-one (7a)

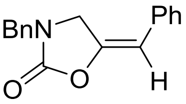

7a

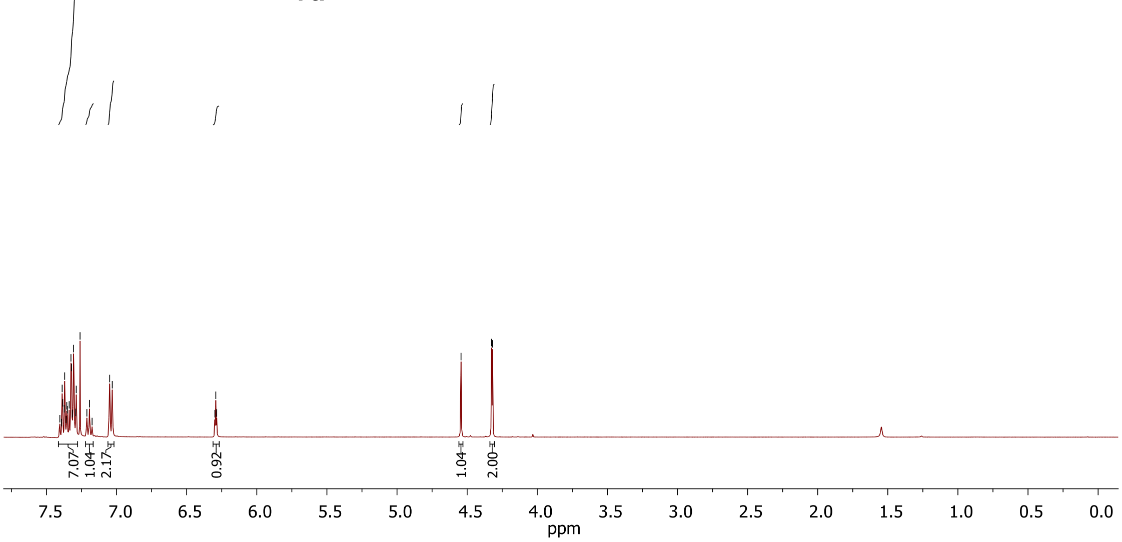

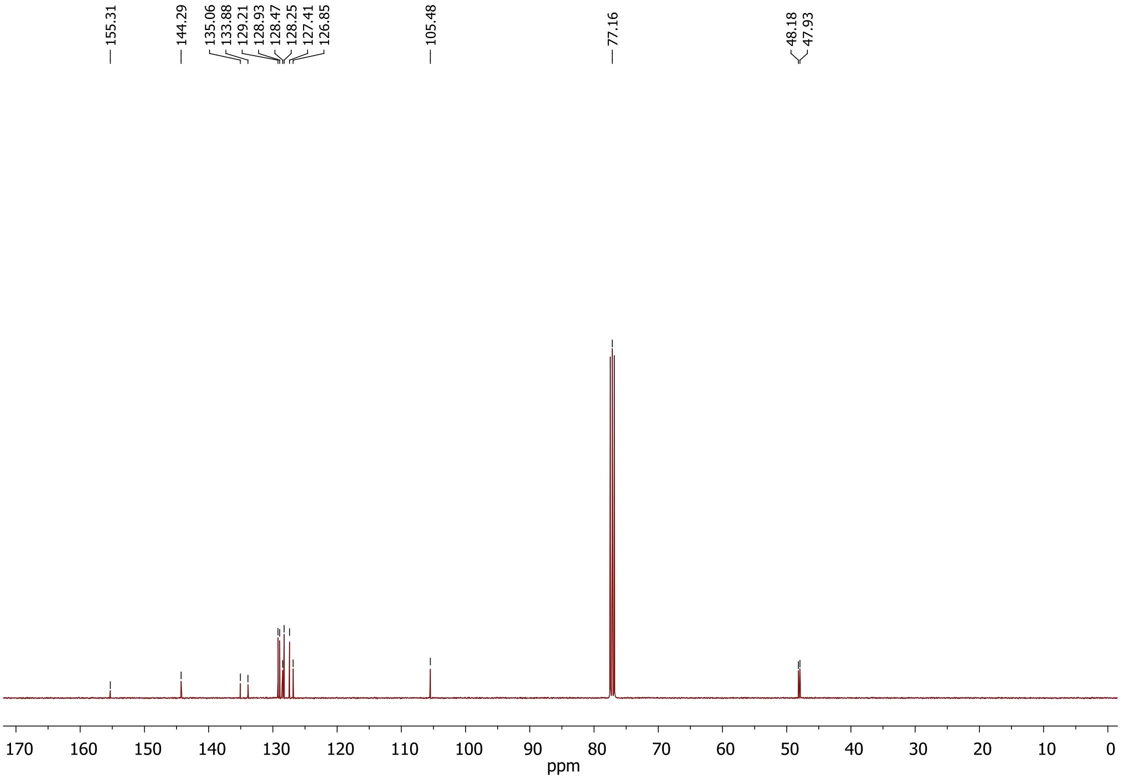

(*E*)-3-Butyl-5-(1-phenylethylidene)-oxazolidin-2-one (8a)

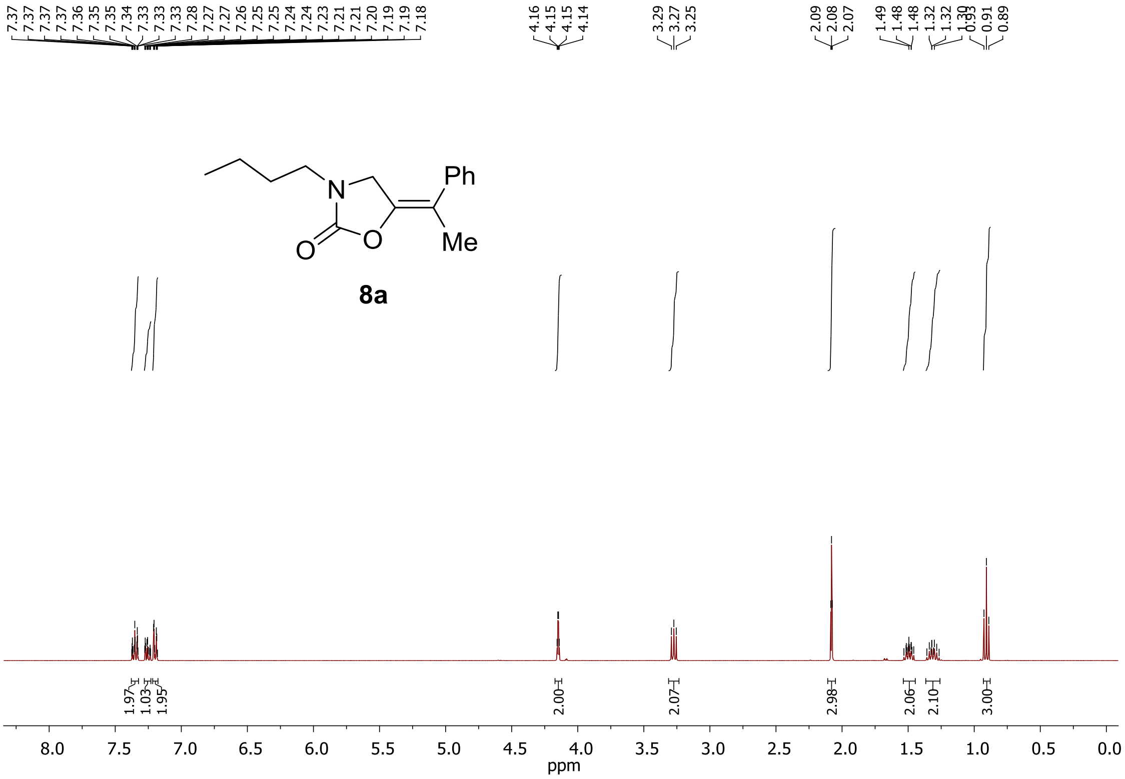

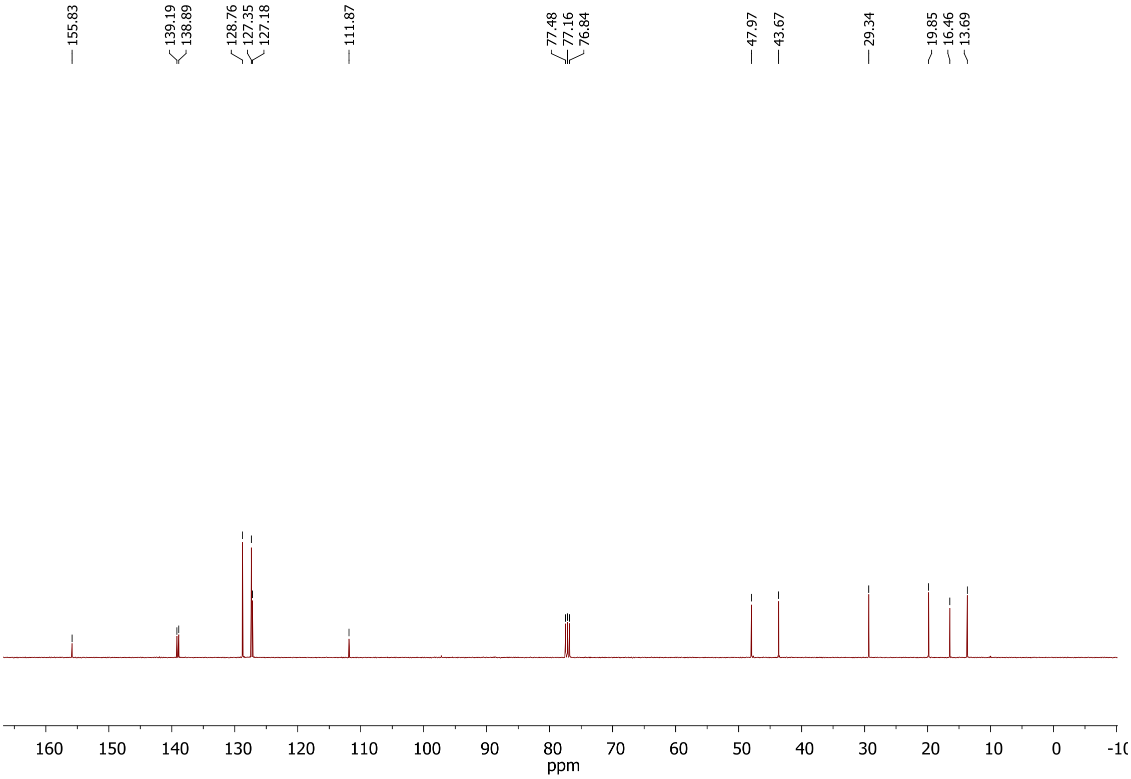

(*E*)-3-*Iso*-propyl-5-(1-phenylethylidene)-oxazolidin-2-one (9a)

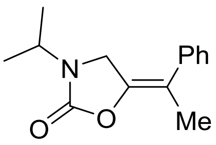

9a

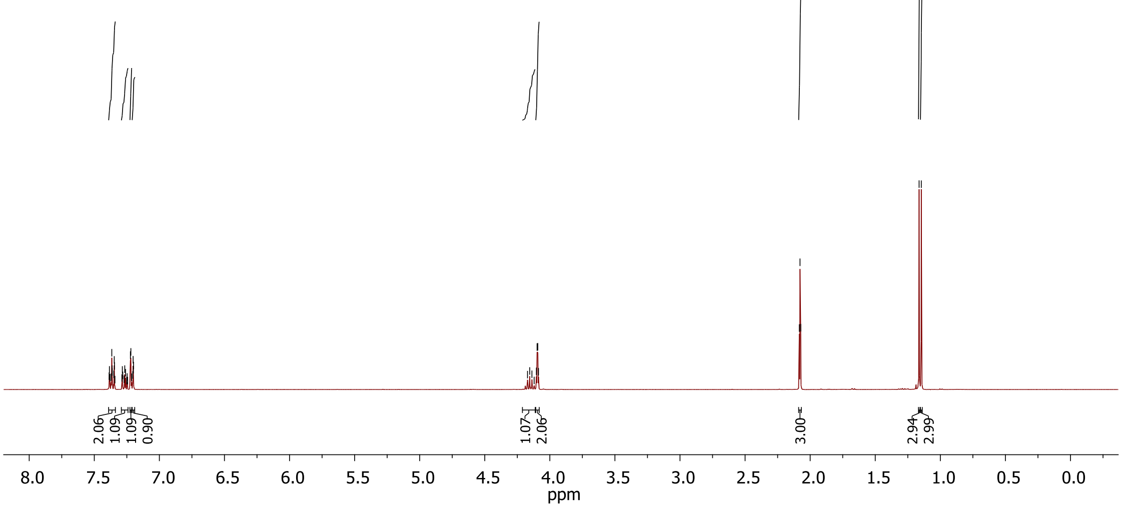

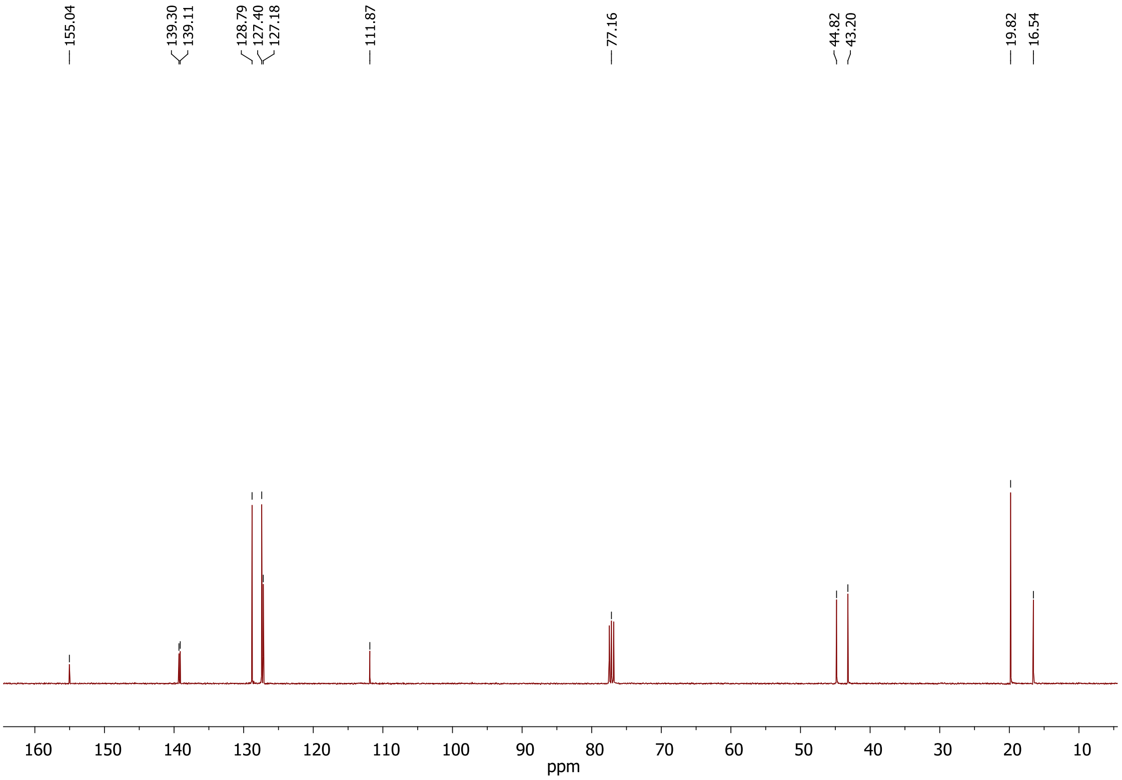

(*E*)-3-Allyl-5-(1-phenylethylidene)-oxazolidin-2-one (10a)

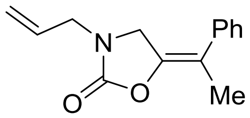

10a

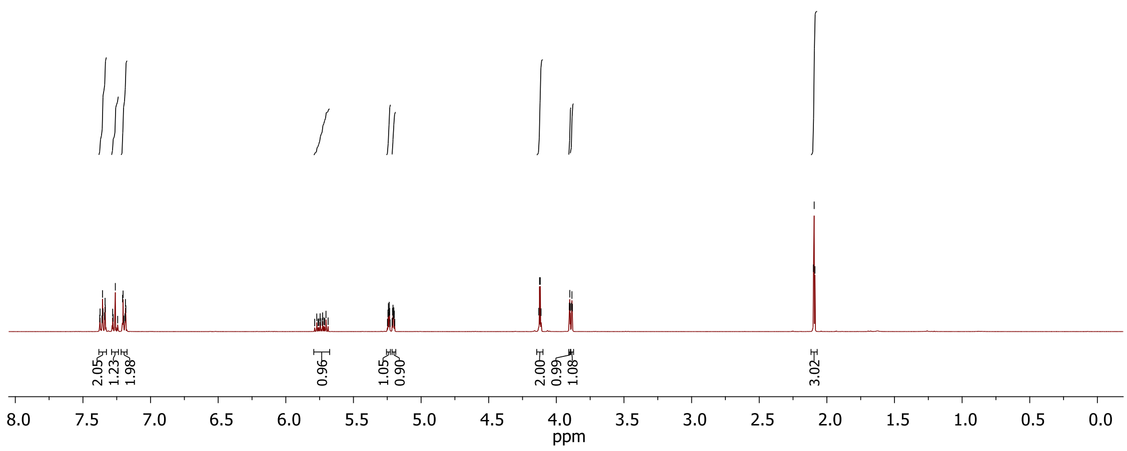

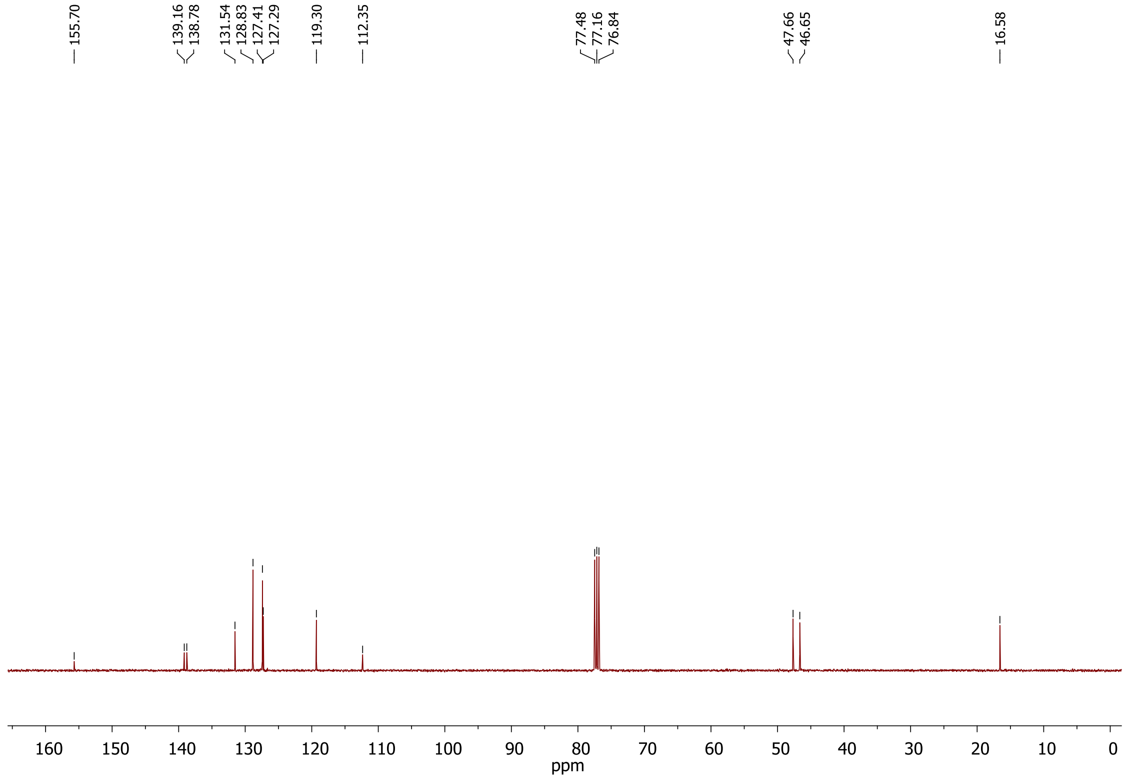

(*E*)-3-*Tert*-butyl-5-(1-phenylethylidene)-oxazolidin-2-one (11a)

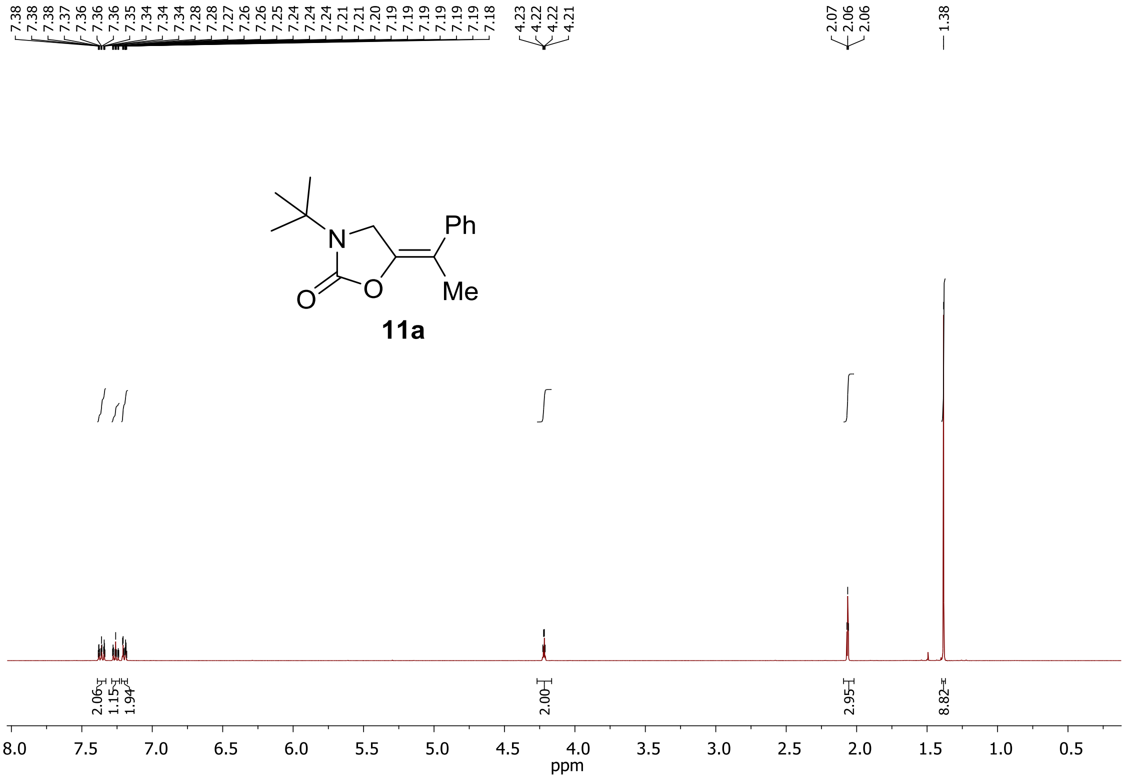

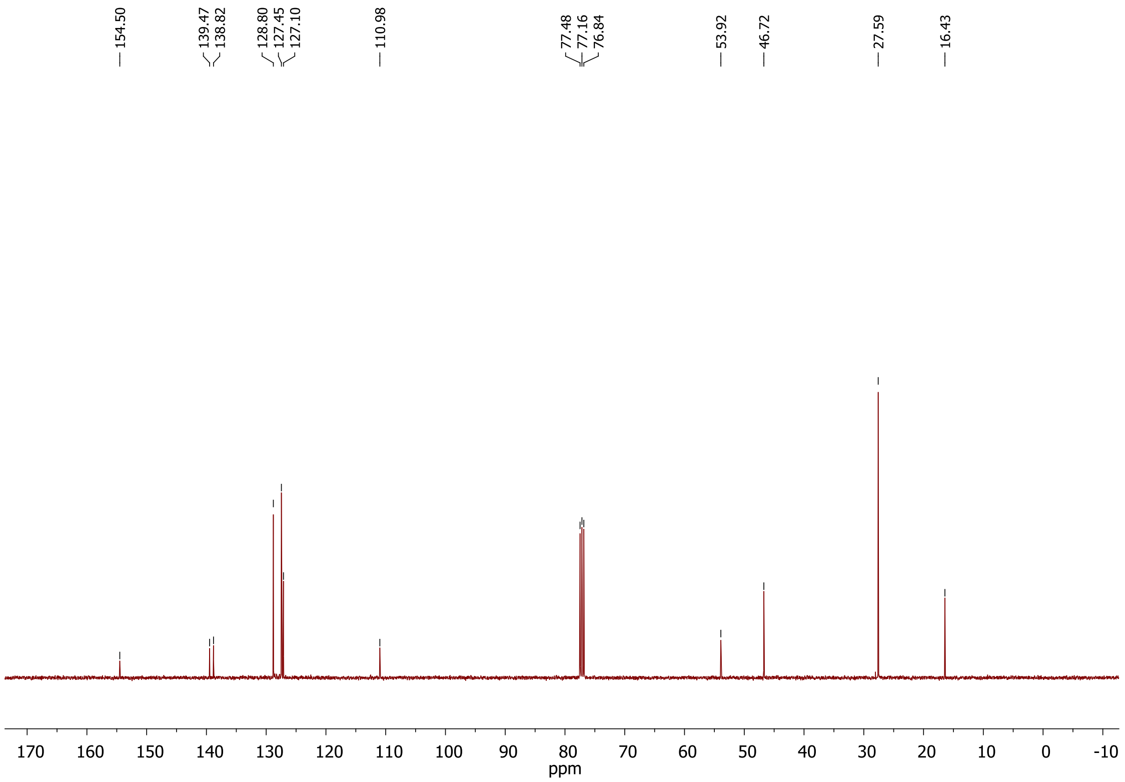

(*E*)-3-Benzyl-5-(diphenylmethylene)-4-methyl-oxazolidin-2-one (12a)

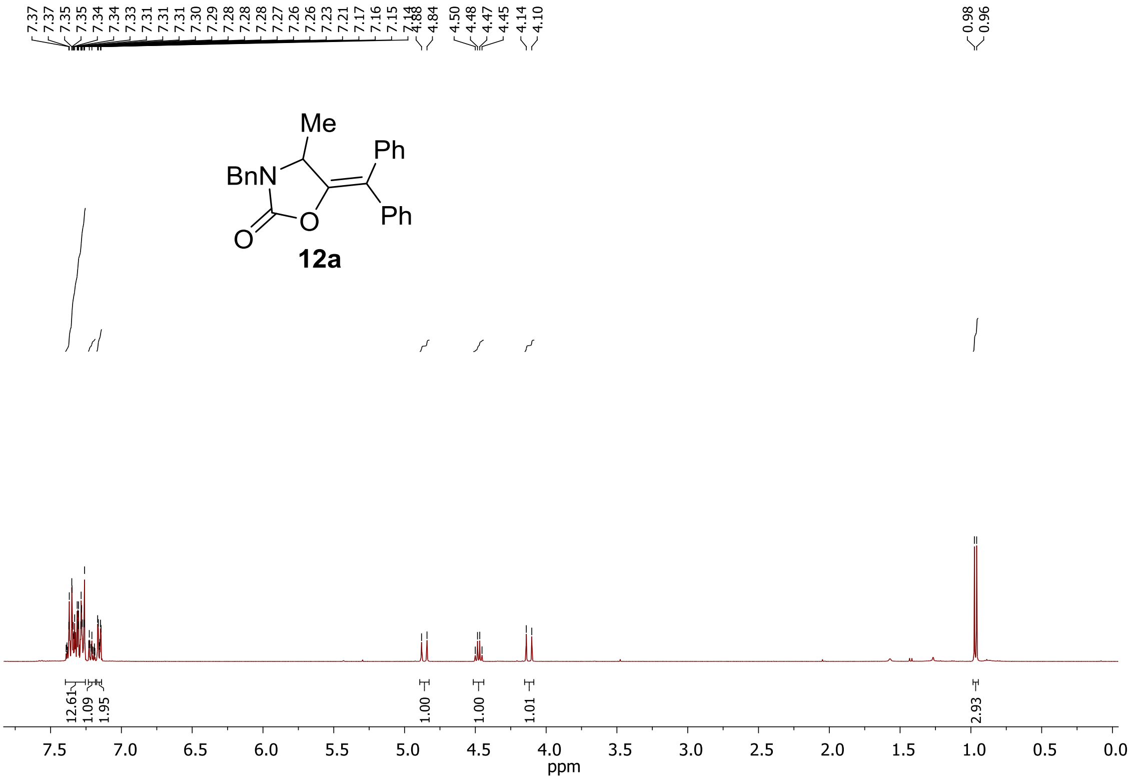

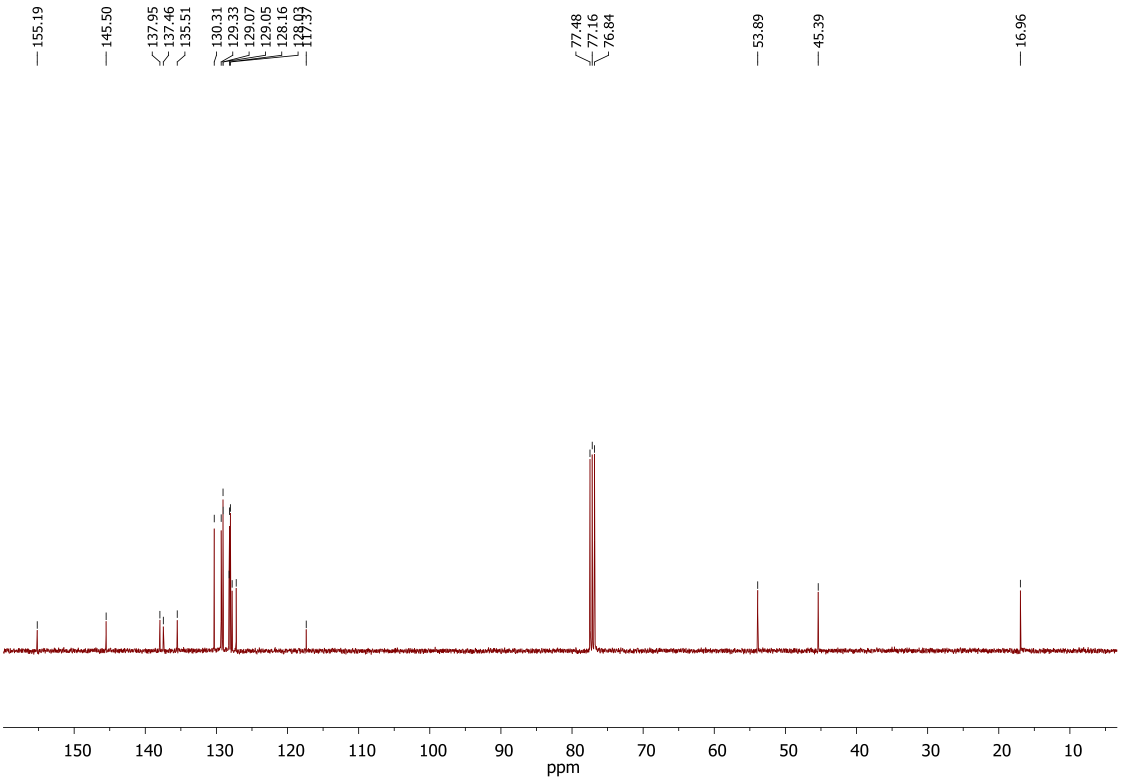

(*E*)-5-benzylideneoxazolidin-2-one (13a)

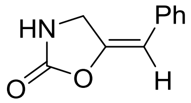

13a

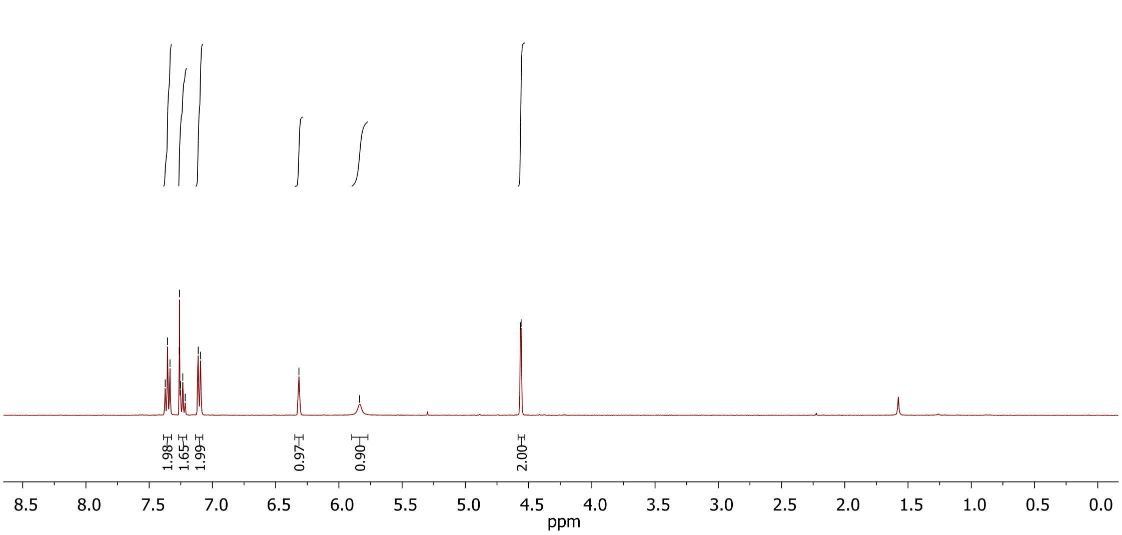

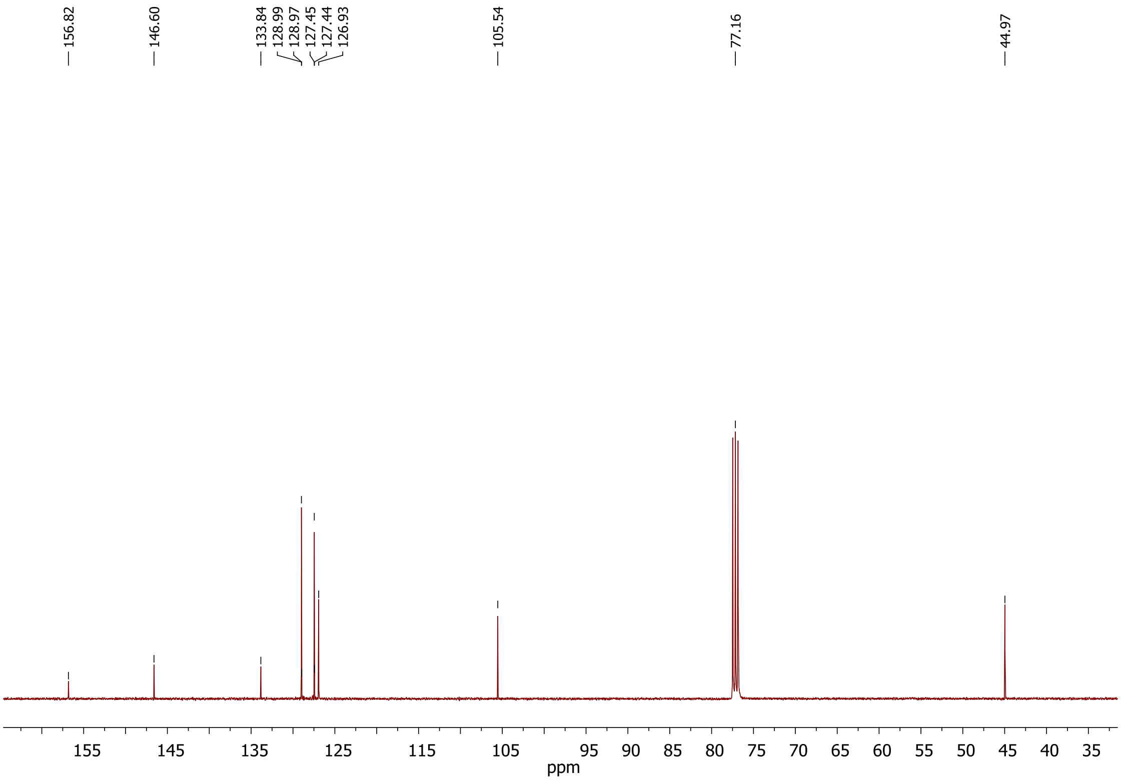

5-(diphenylmethylene)-3-(4-methoxybenzyl)oxazolidin-2-one (15)

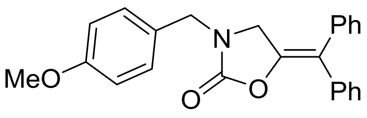

15

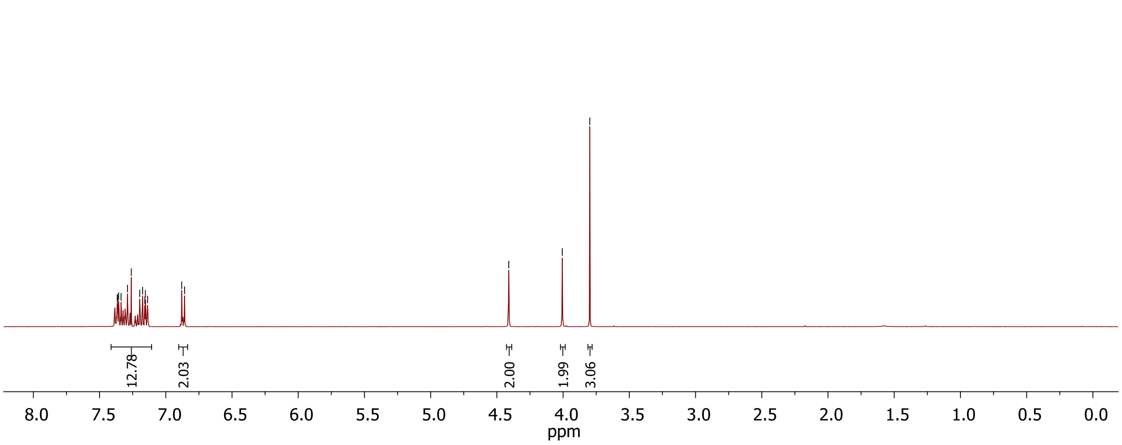

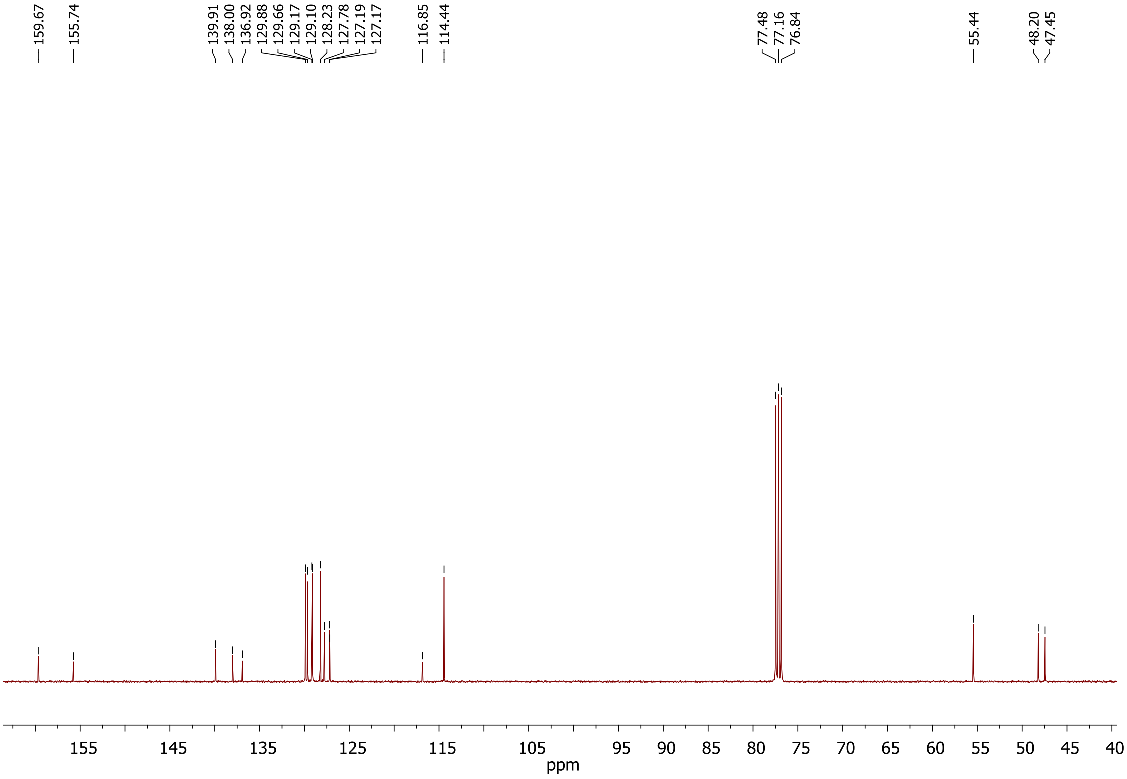

5-(diphenylmethylene)-3-*Iso*-propyloxazolidin-2-one (16)

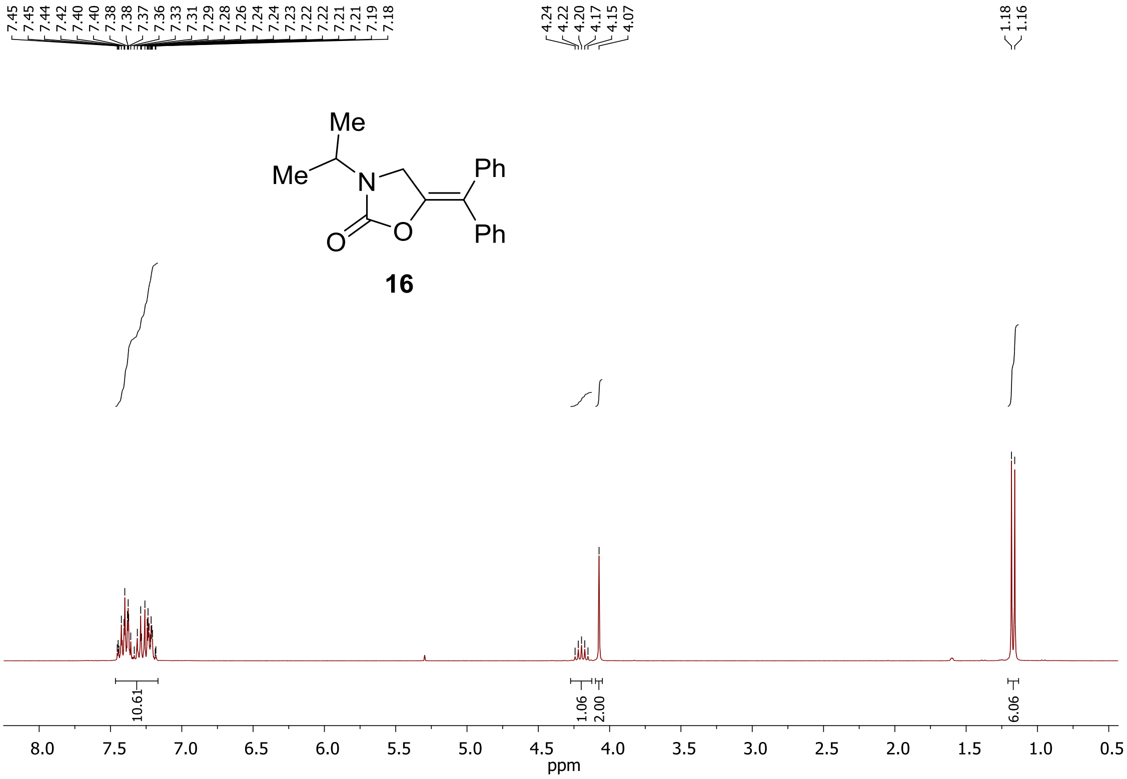

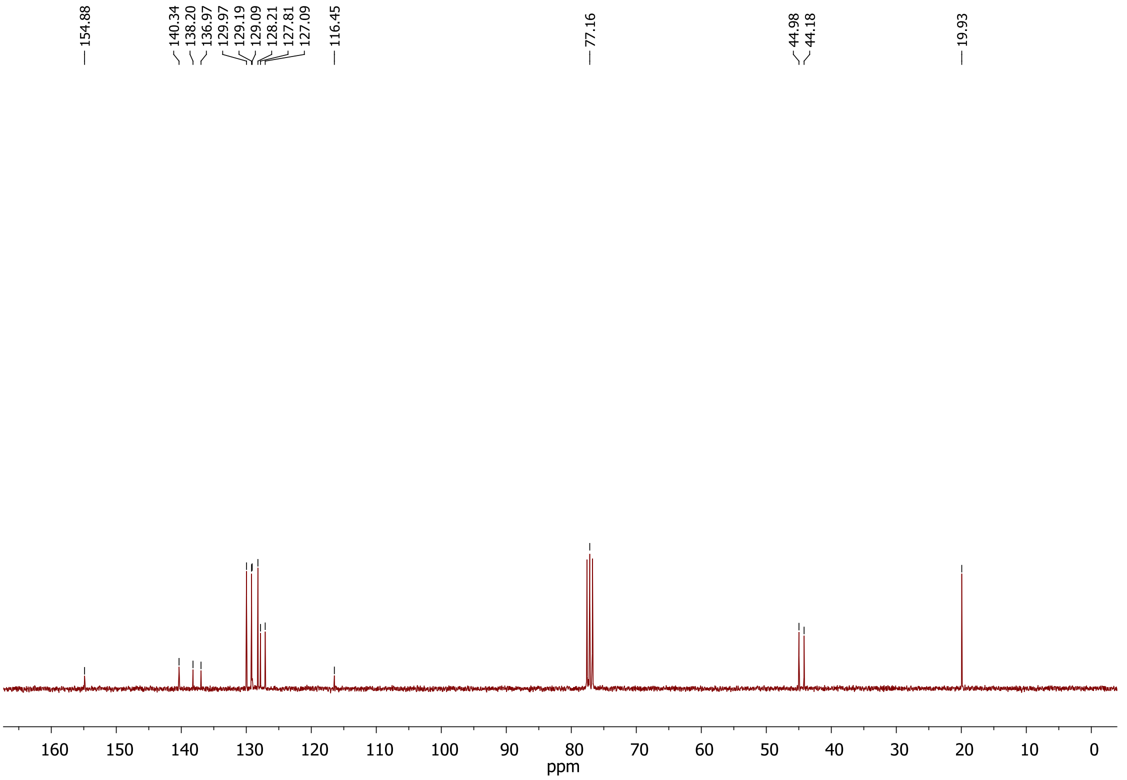

3-Benzyl-5-[bis(3-methoxyphenyl)methylene]oxazolidin-2-one (17)

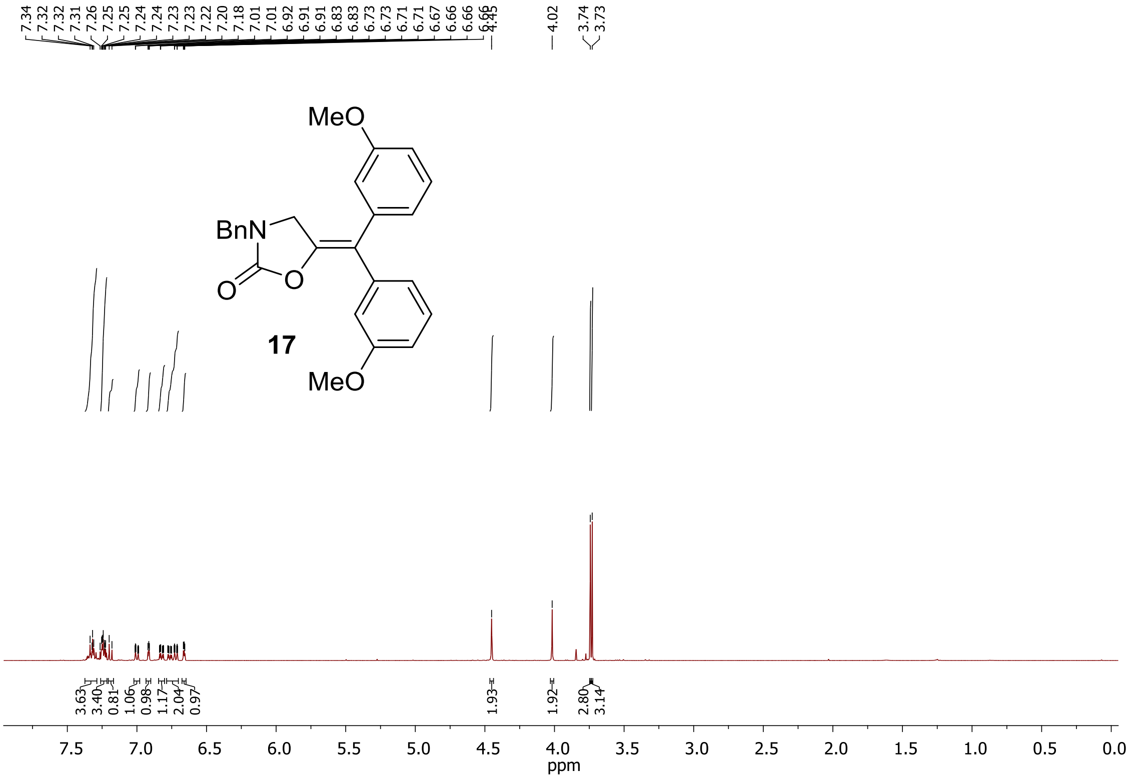

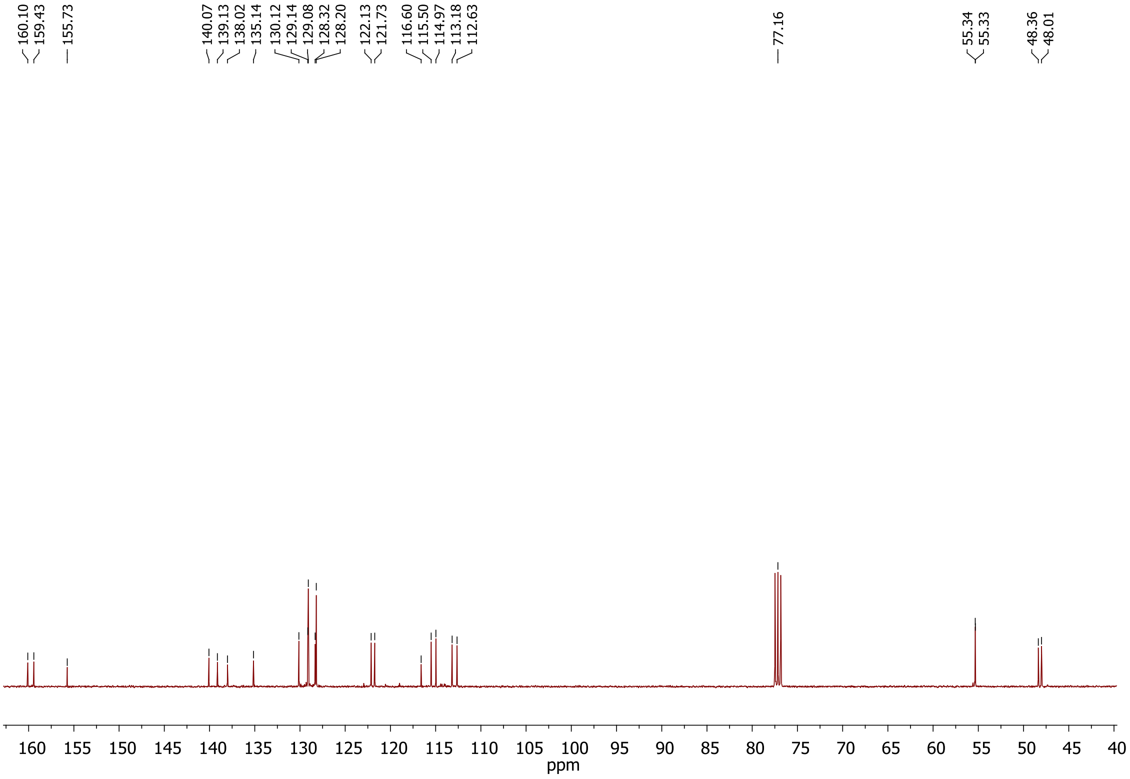

3-Benzyl-5-[bis(3-(methoxymethoxy)phenyl)methylene]oxazolidin-2-one (18)

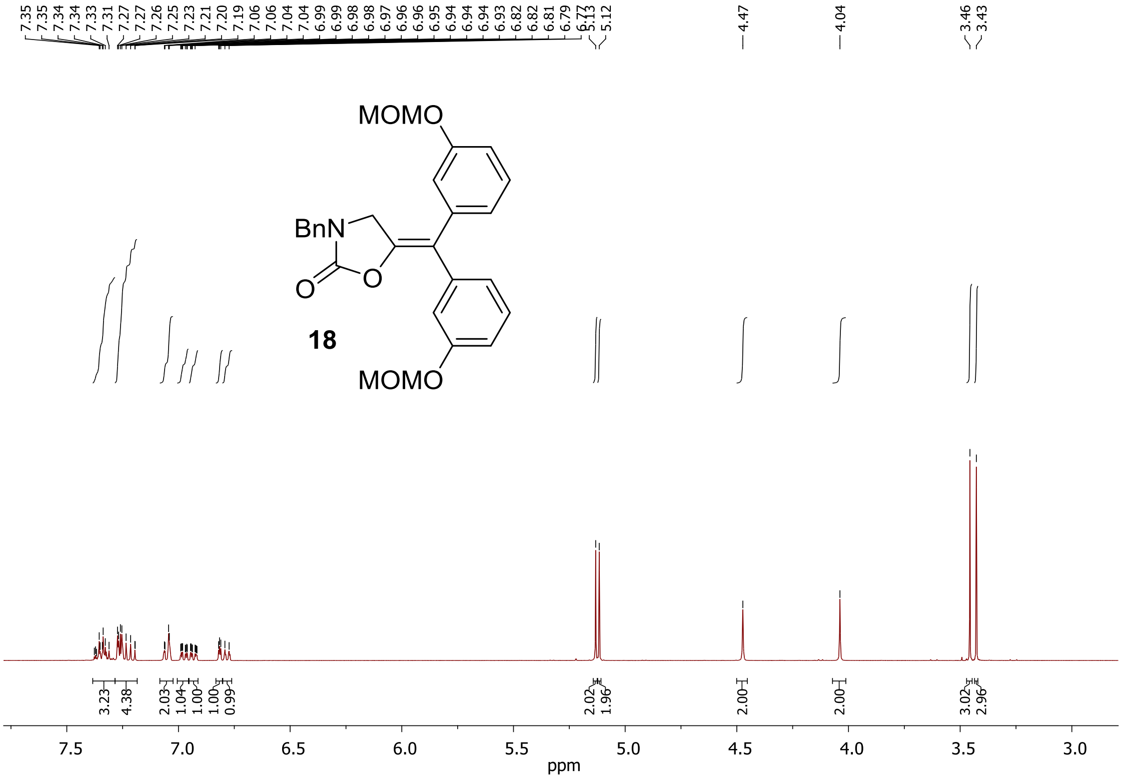

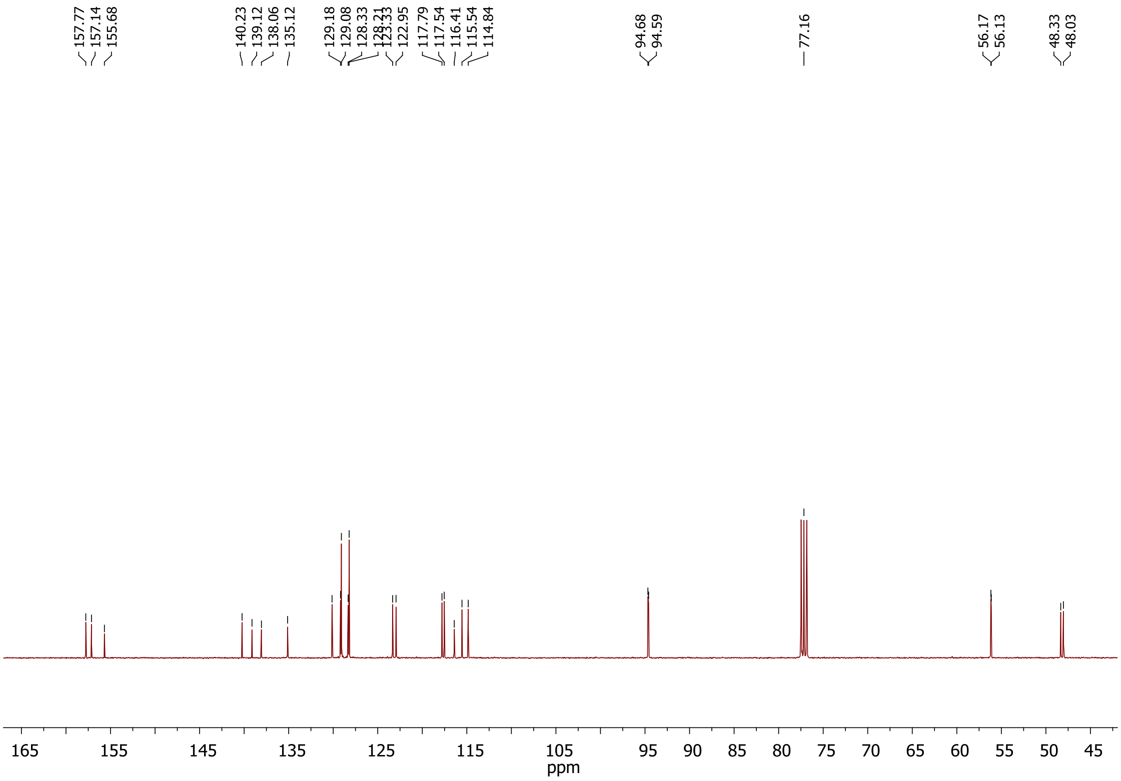

3-Benzyl-5-[bis(3-(bencyloxy)phenyl)methylene]oxazolidin-2-one (19)

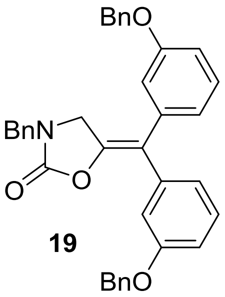

19

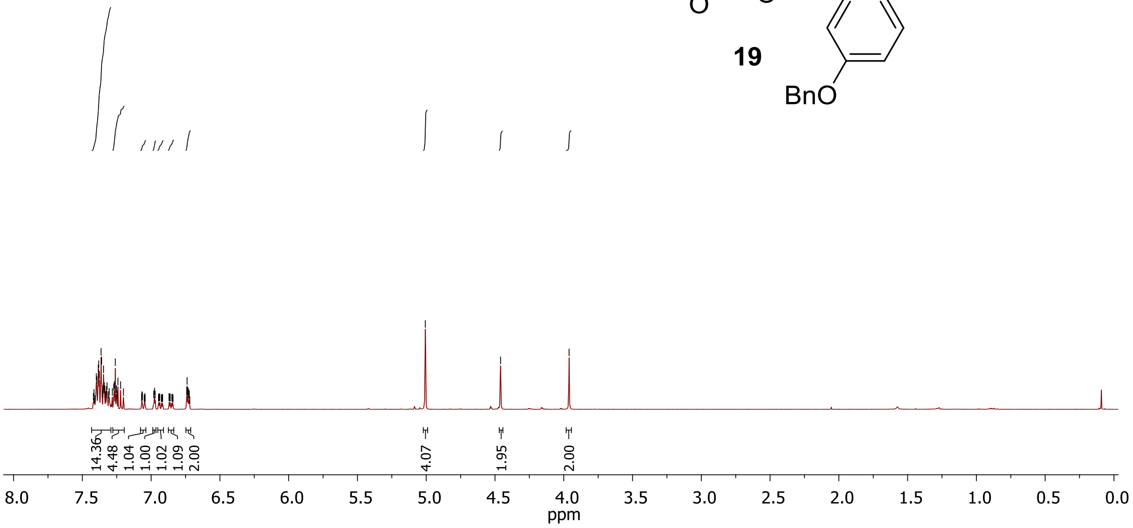

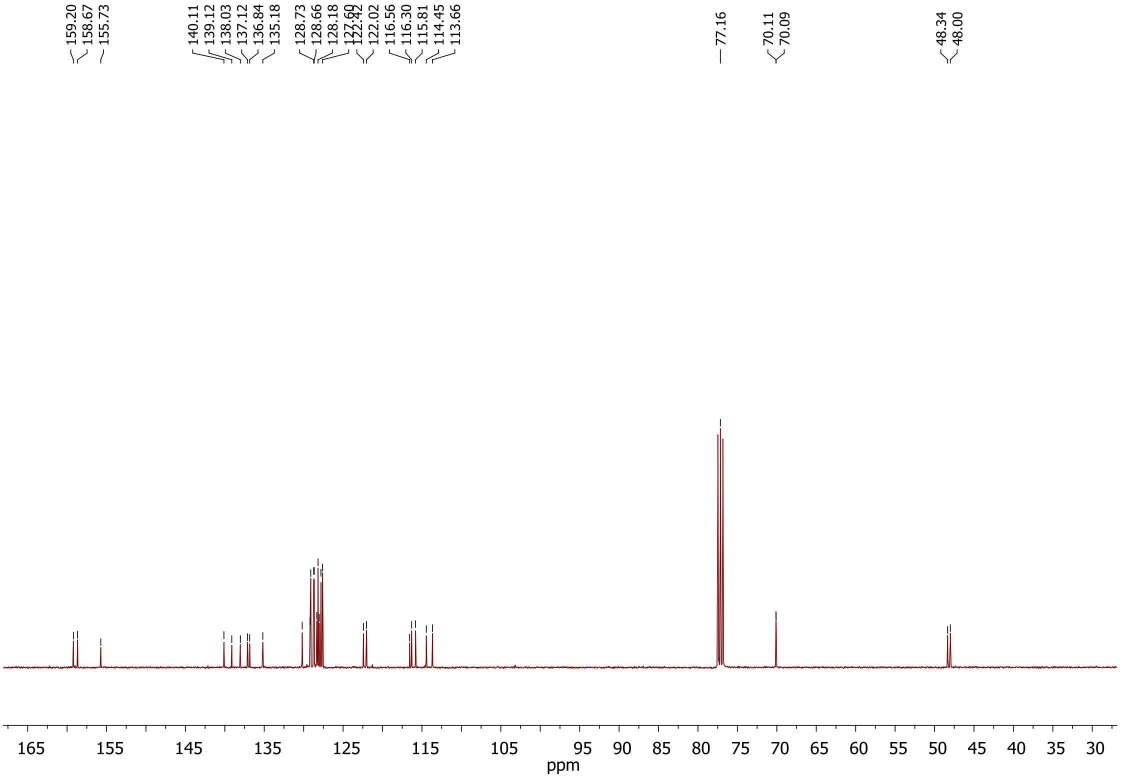

3-Benzyl-5-(bis-m-tolylmethylene)oxazolidin-2-one (20)

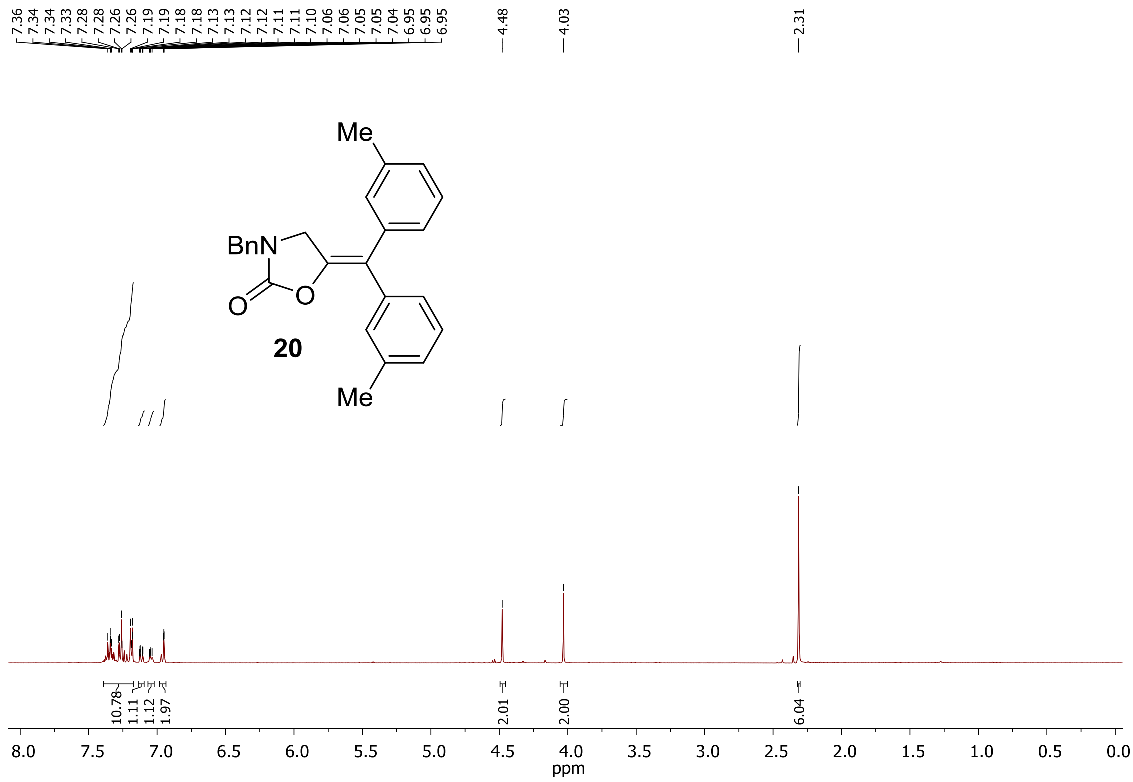

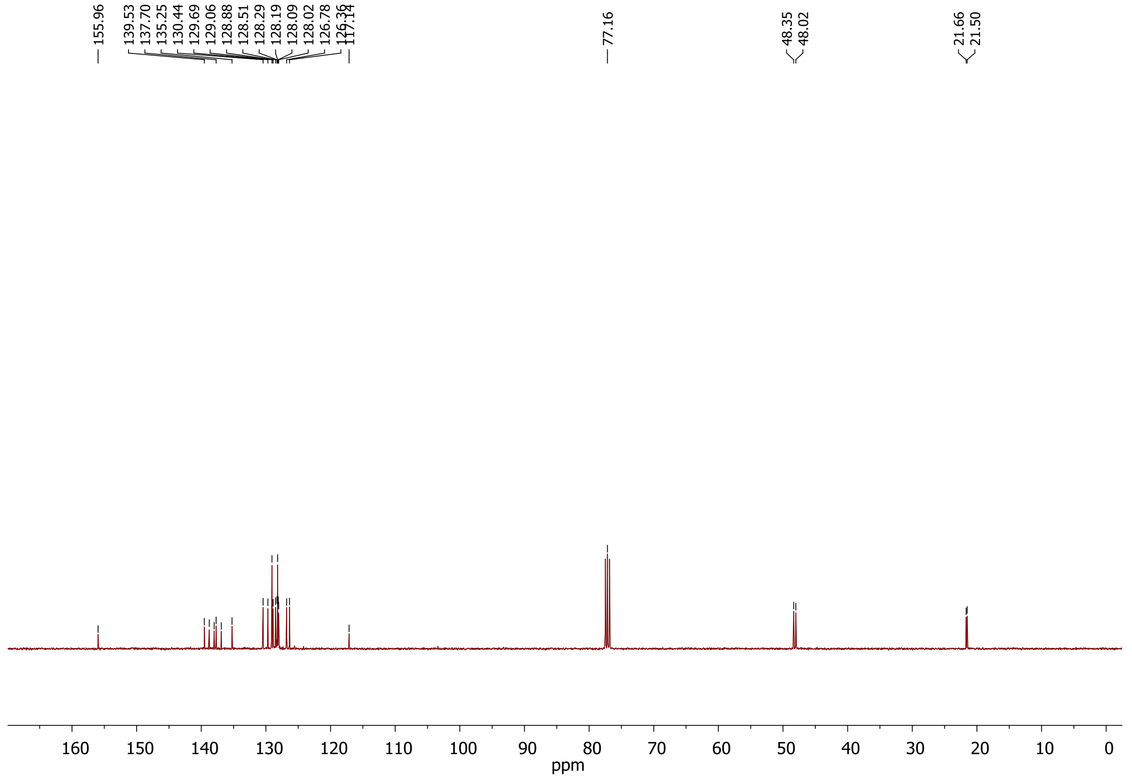

3-Benzyl-5-[bis-(4-bromo-3-methoxyphenyl)methylene]oxazolidin-2-one (21)

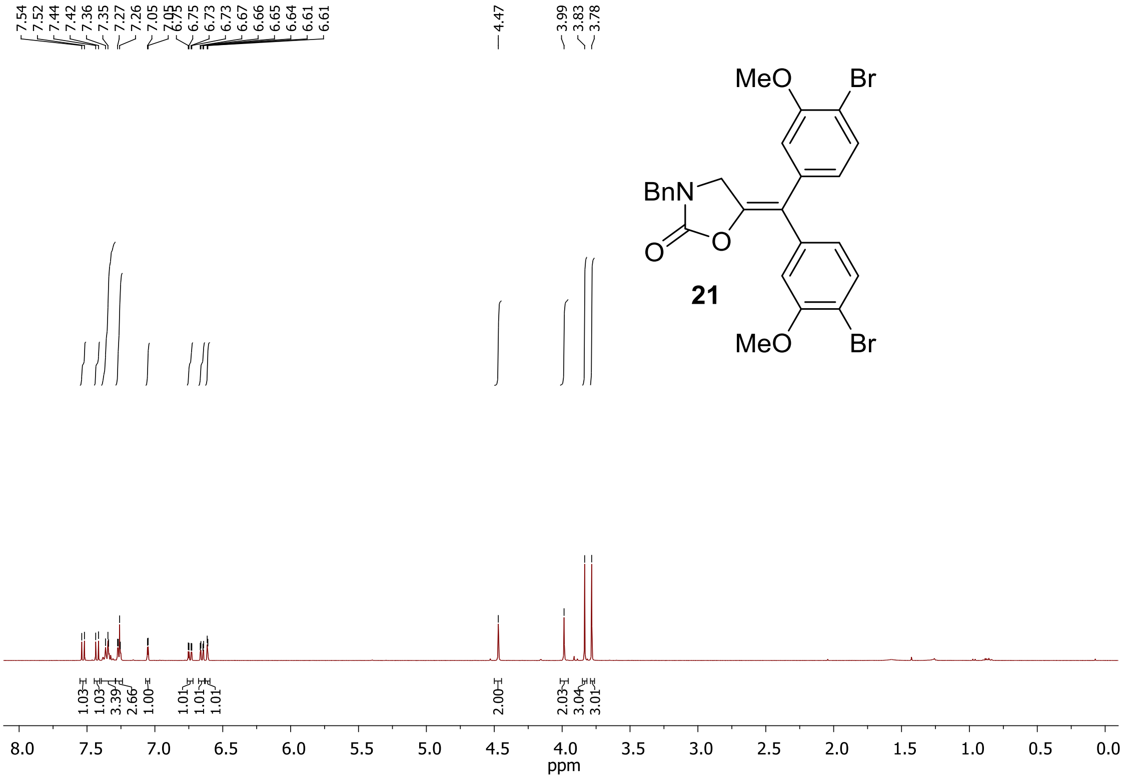

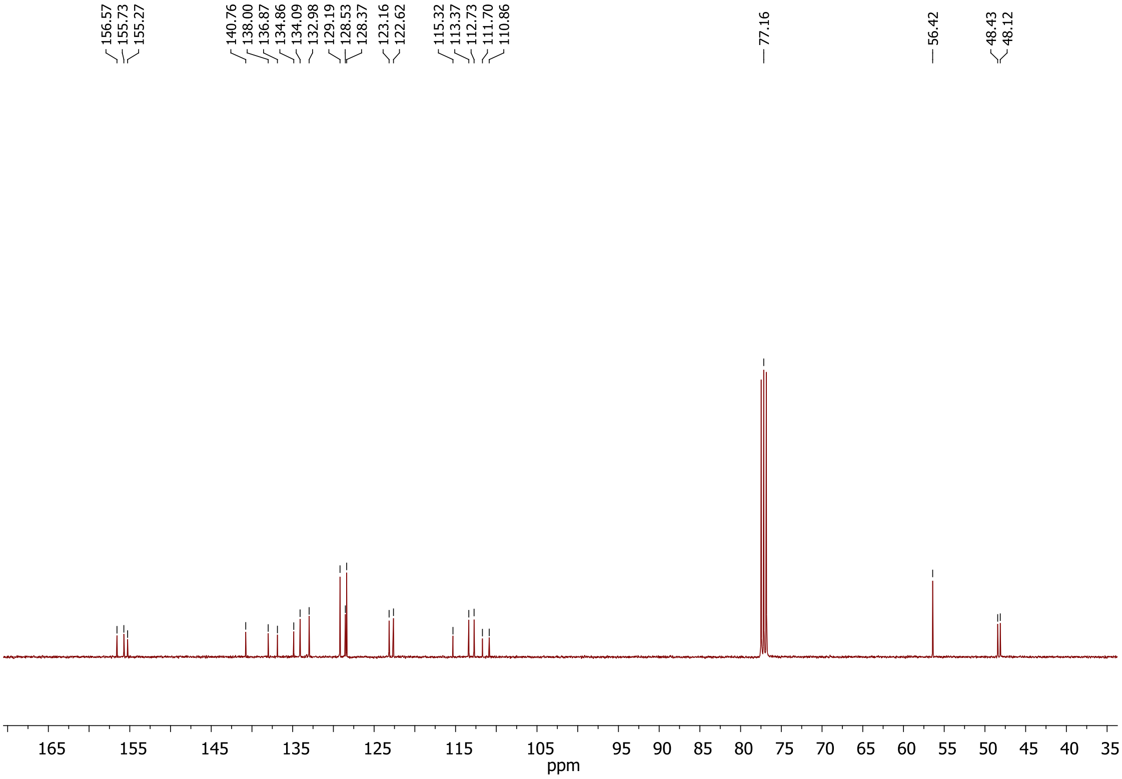

3-Benzyl-5-[bis-(3,5-dimethoxyphenyl)methylene]oxazolidin-2-one (22)

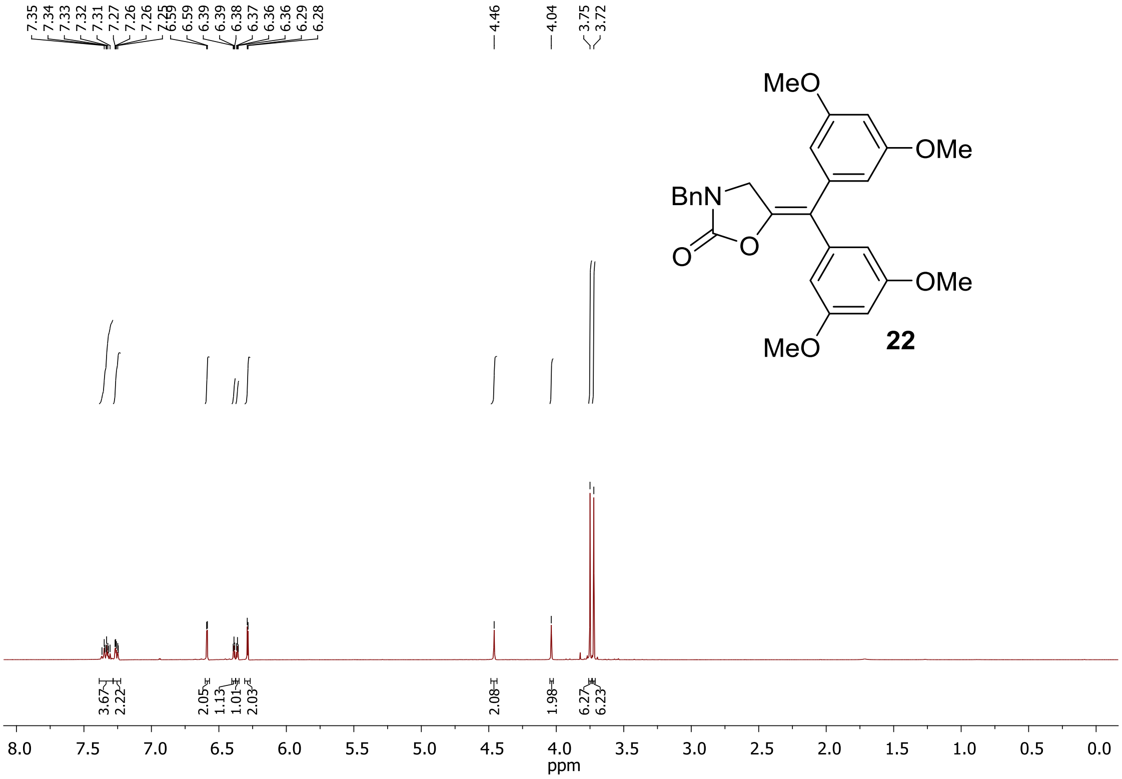

22

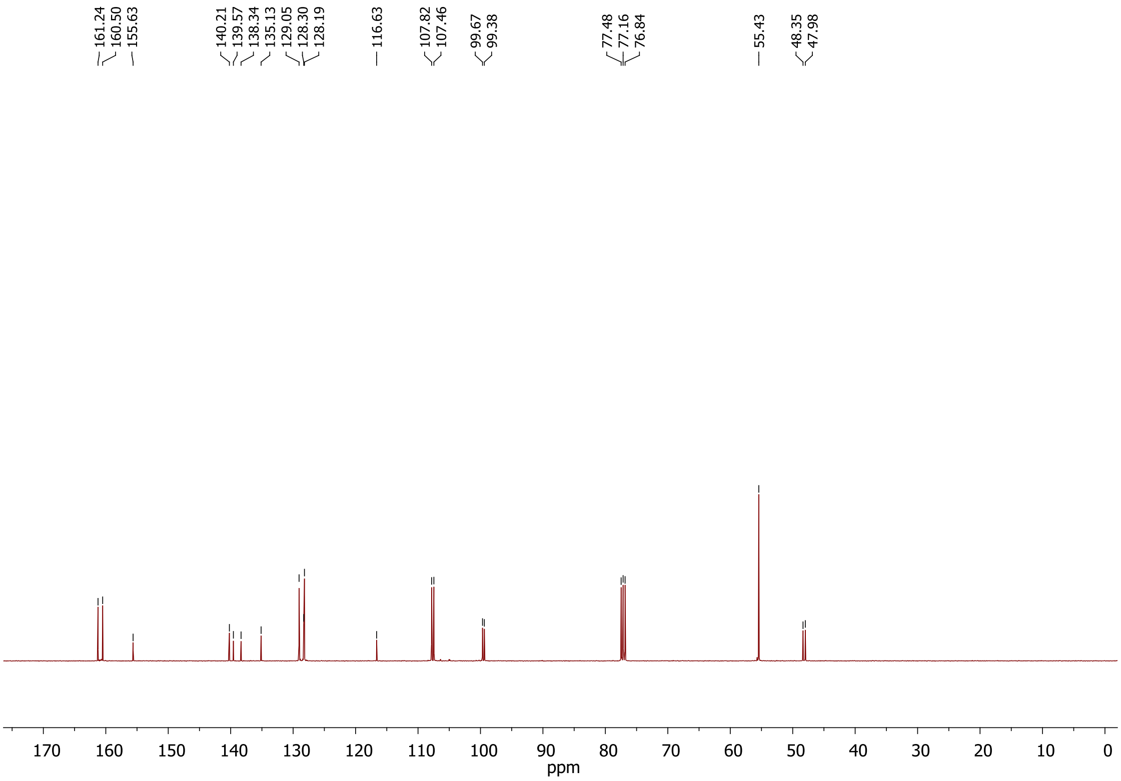

3-Benzyl-5-[bis-(3,4,5-trimethoxyphenyl)methylene]oxazolidin-2-one (23)

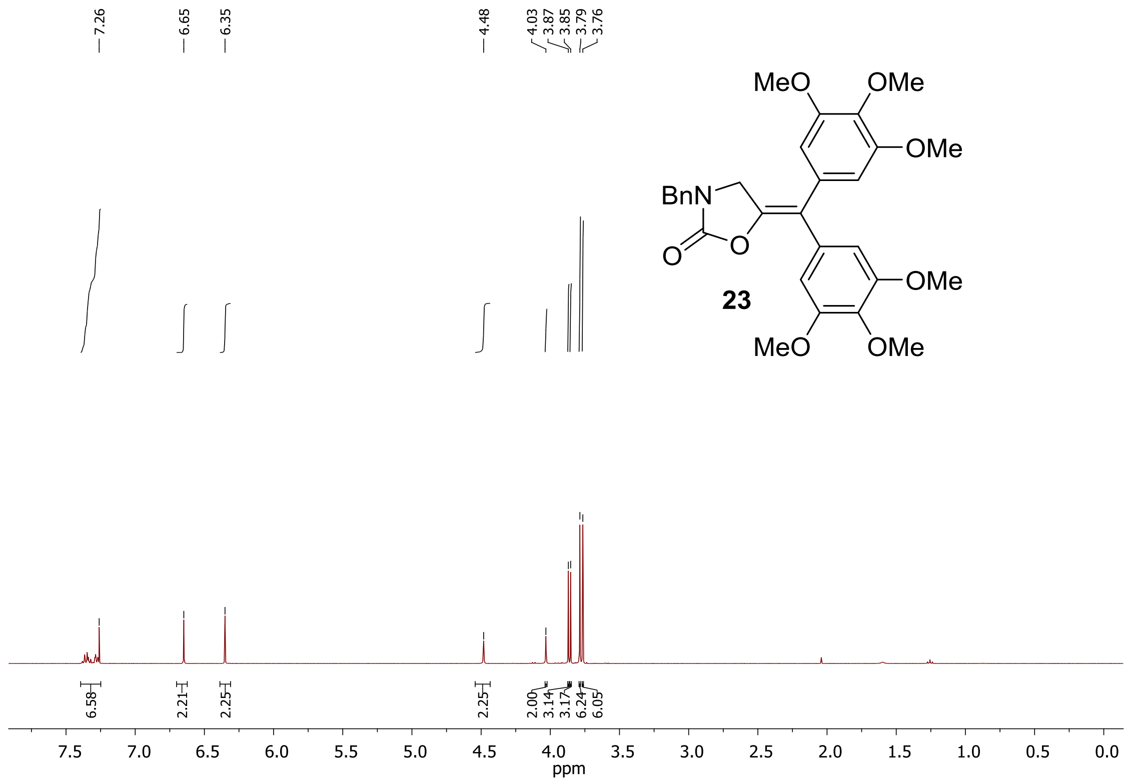

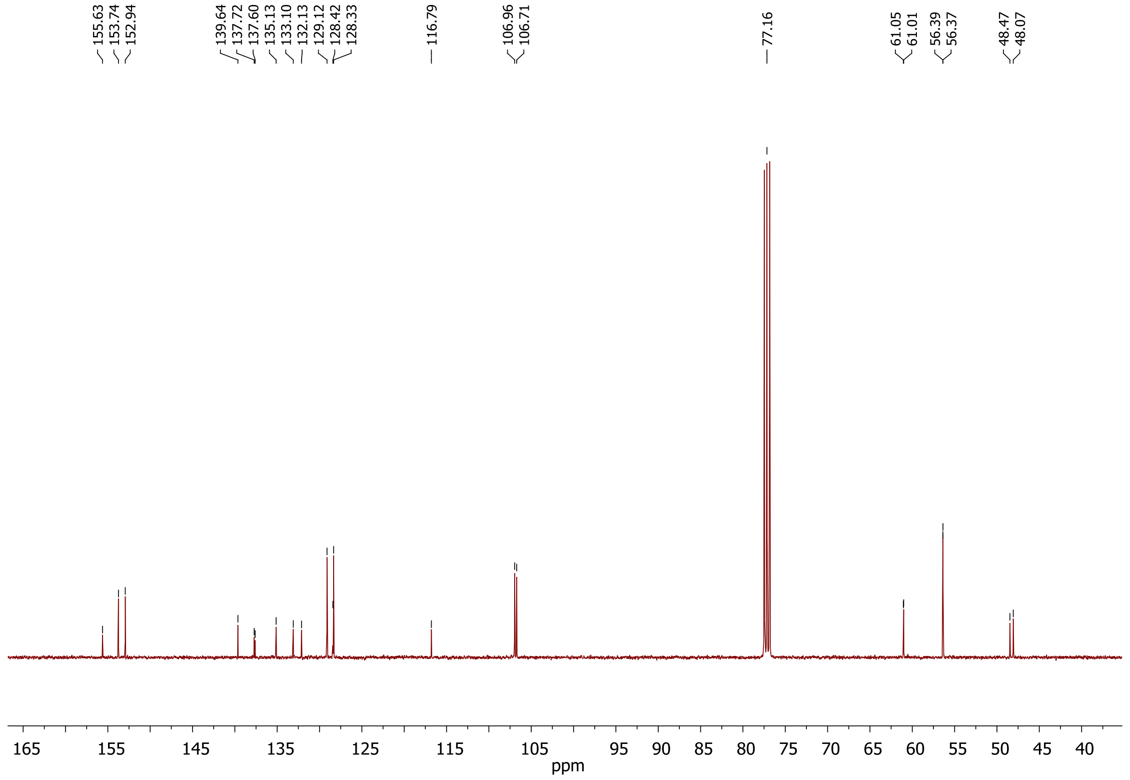

3-Benzyl-5-[bis-(4-methoxyphenyl)methylene]oxazolidin-2-one (24)

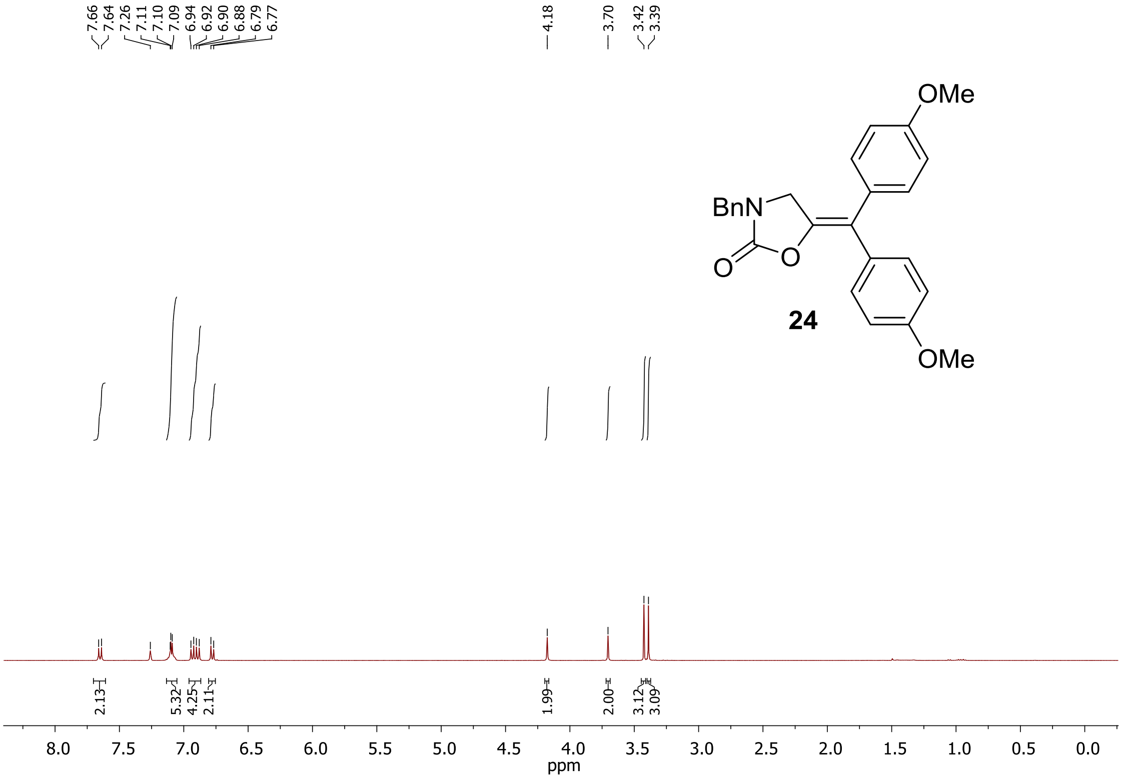

24

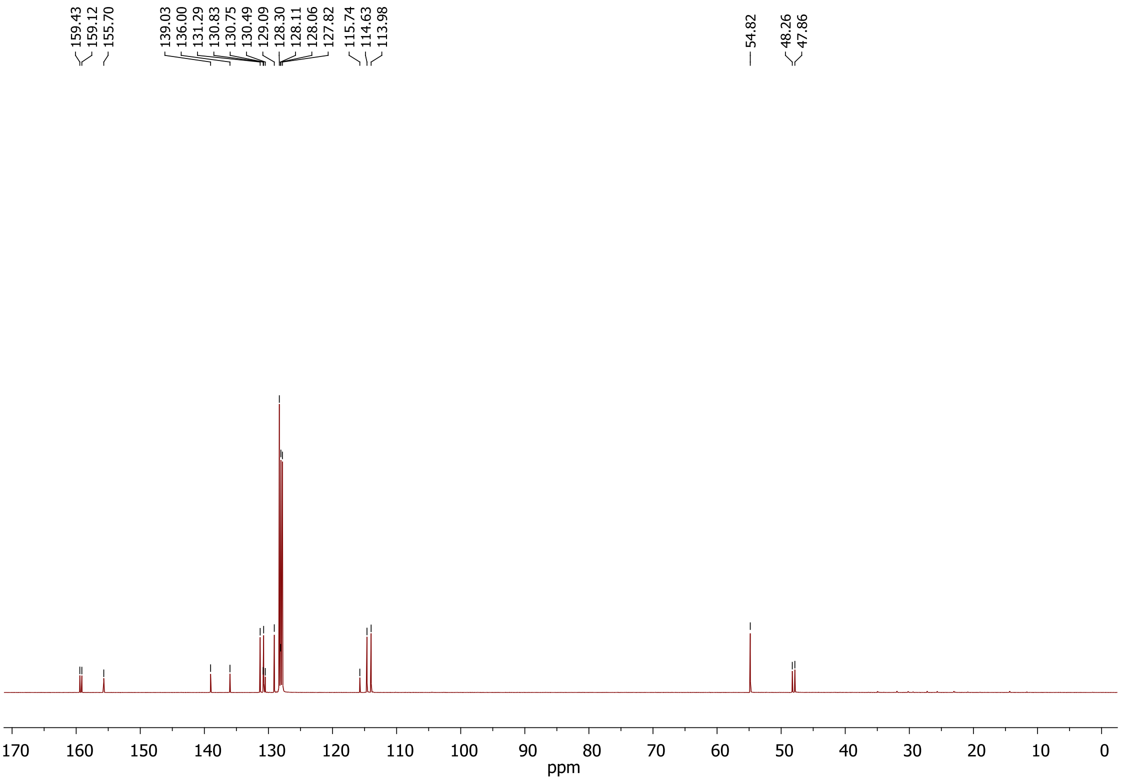

3-Benzyl-5-(bis-(p-tolylmethylene)oxazolidin-2-one (25)

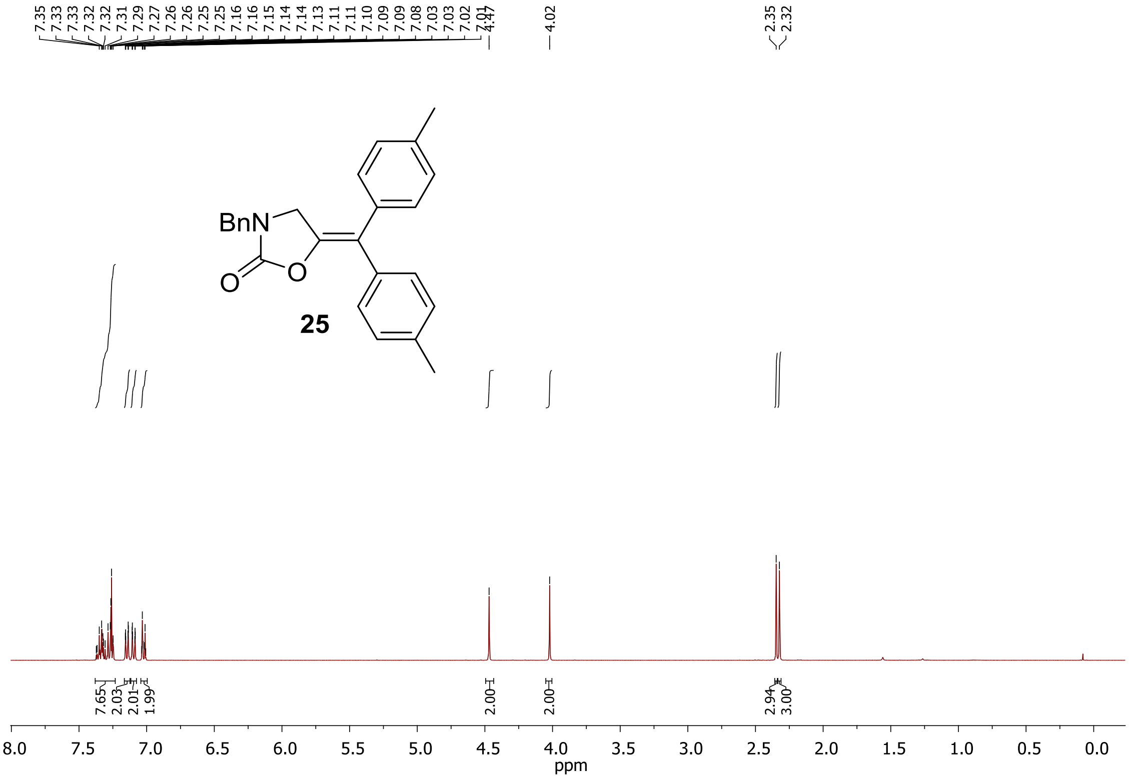

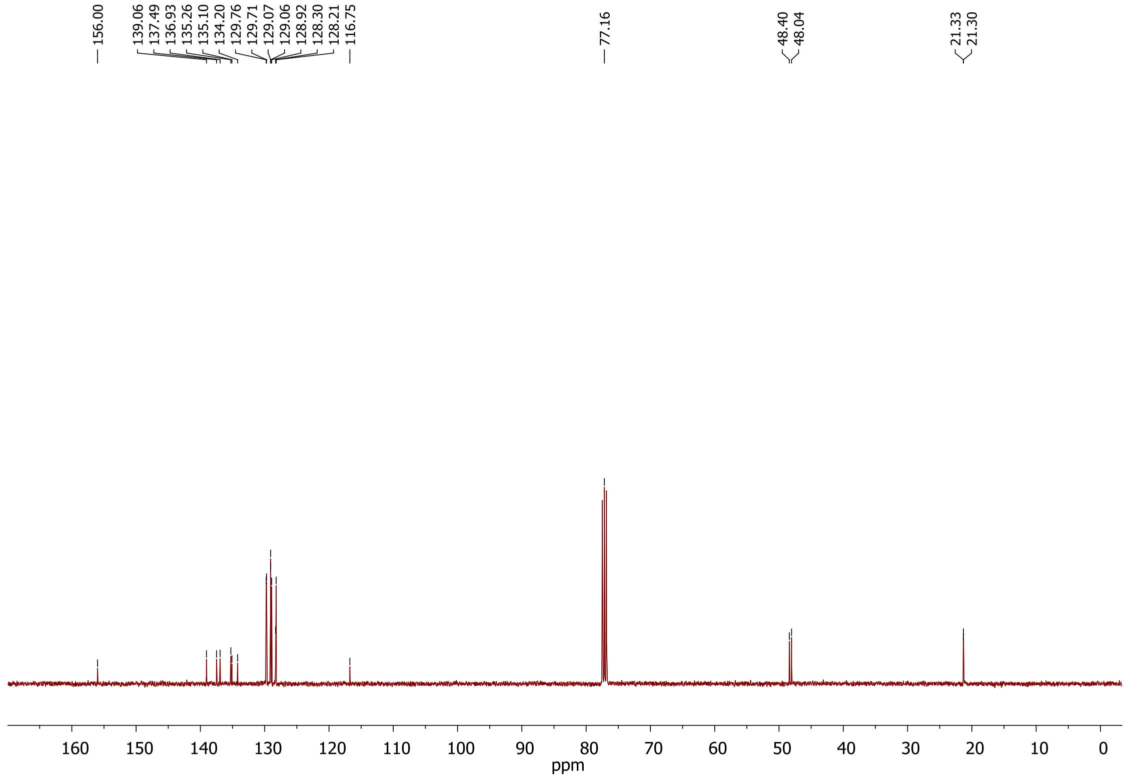

3-Benzyl-5-(di(thiophen-2-yl)methylene)oxazolidin-2-one (26)

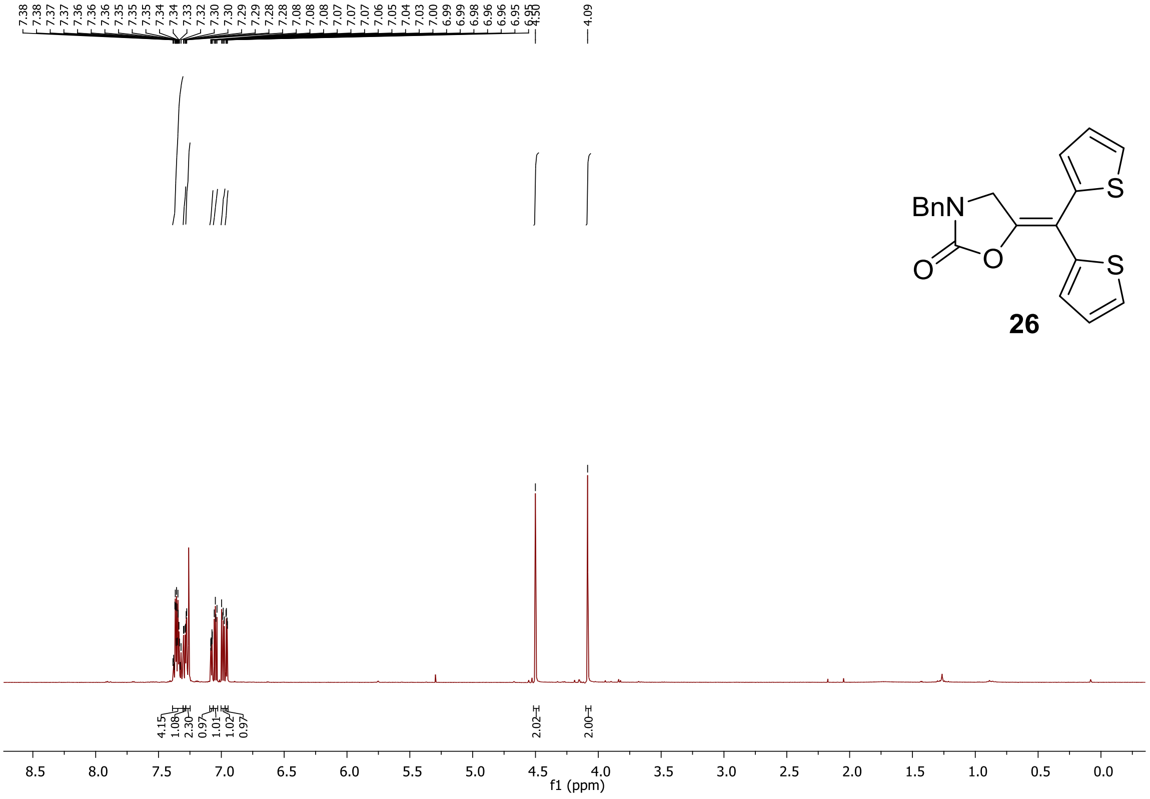

26

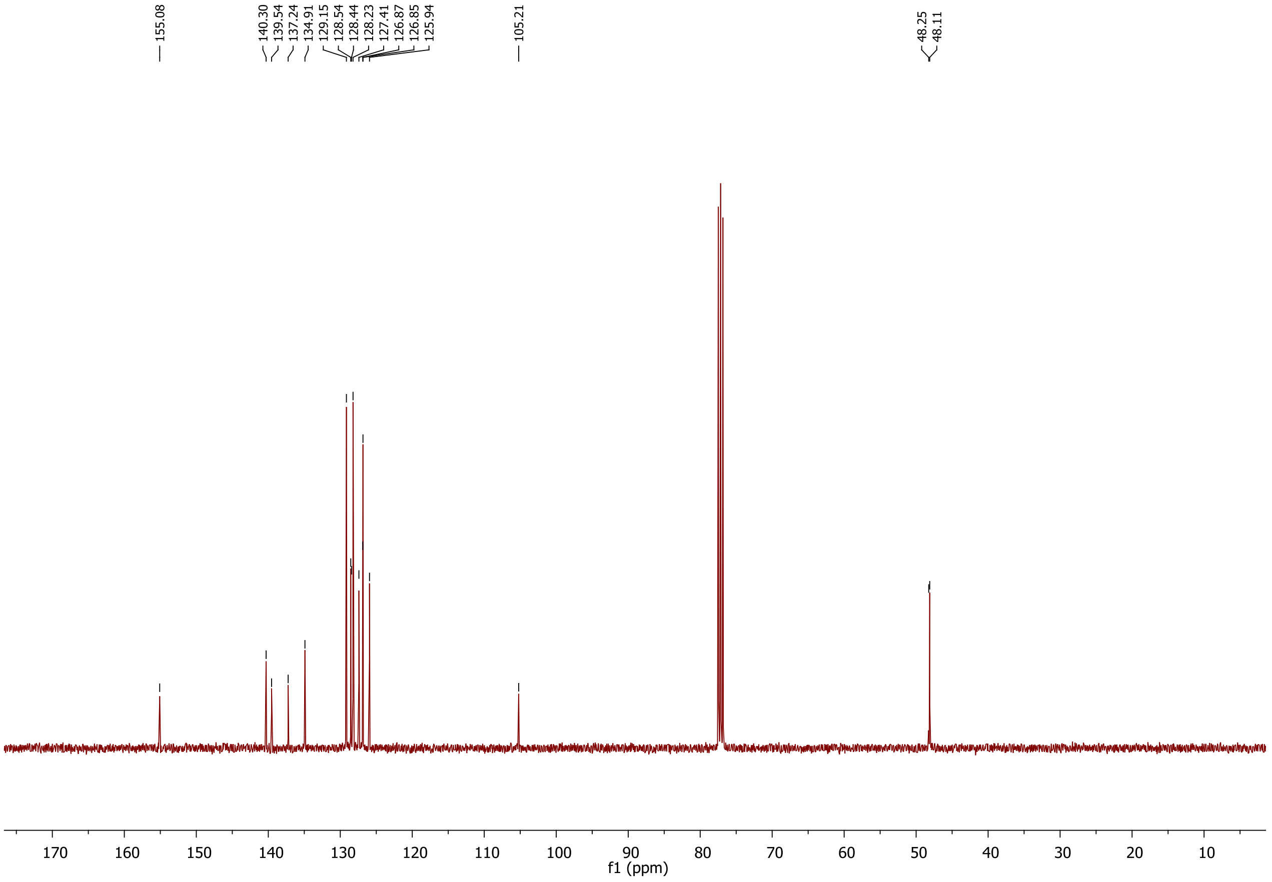

N-Benzyl-3-[4-(trifluoromethyl)phenyl]prop-2-yn-1-amine (30)

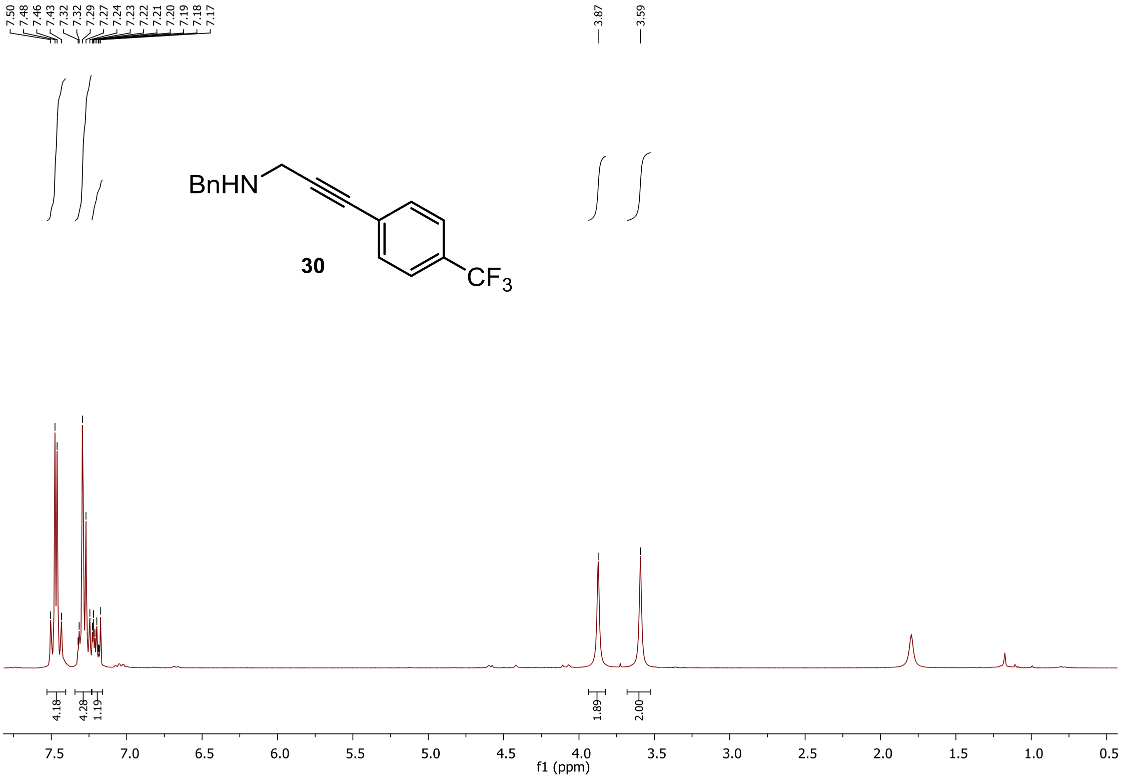

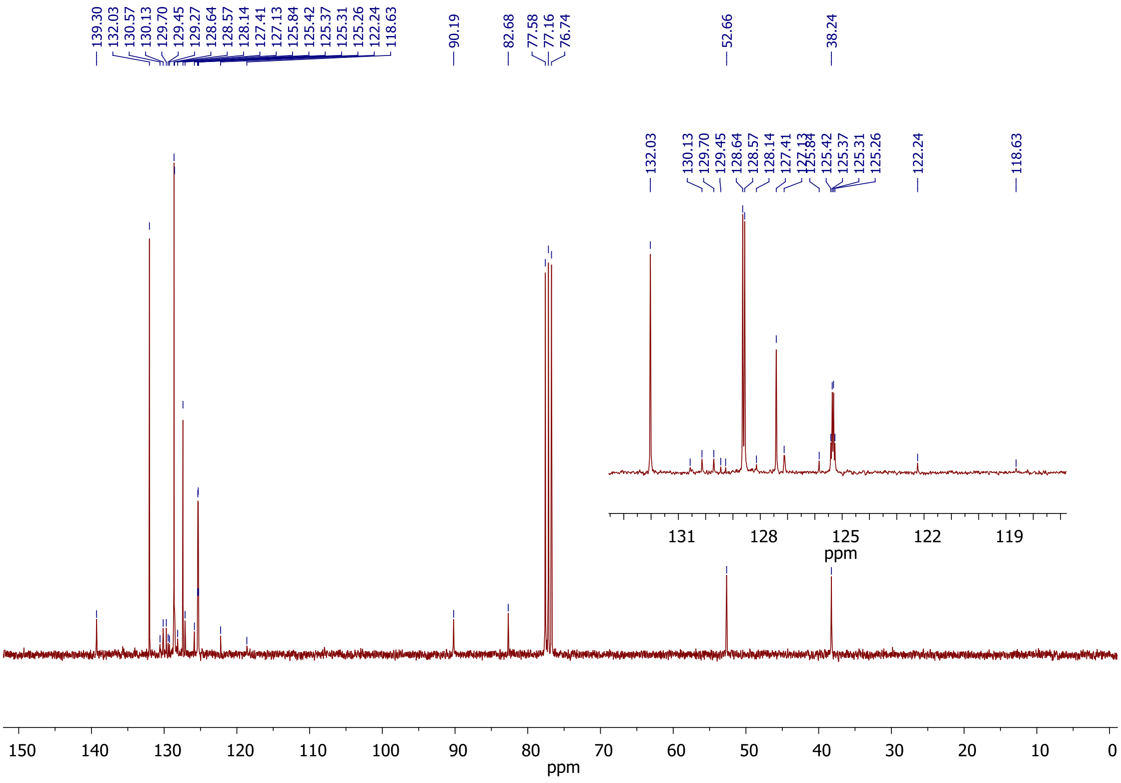

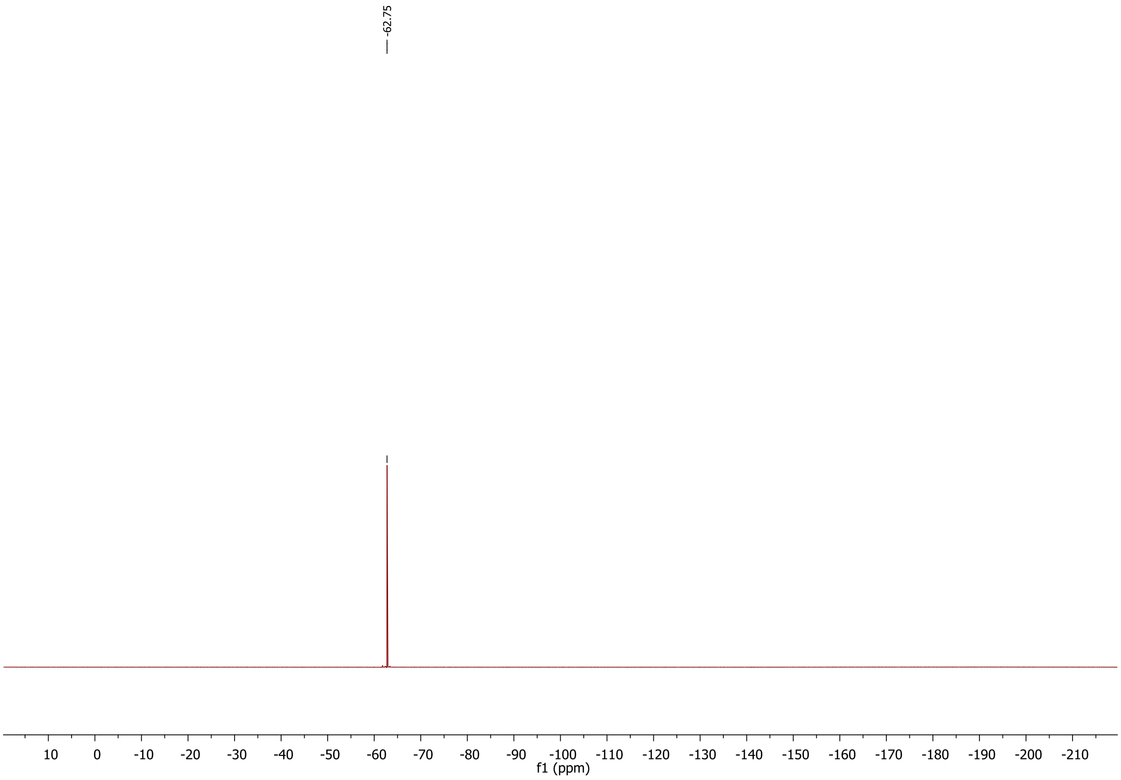

(*Z*)-3-Benzyl-5-[phenyl(4-(trifluoromethyl)phenyl)methylene]oxazolidin-2-one (32)

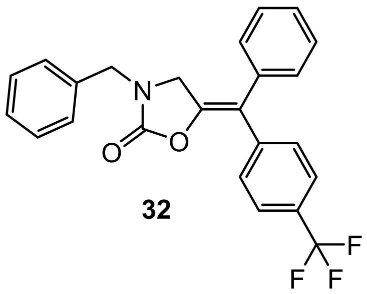

32

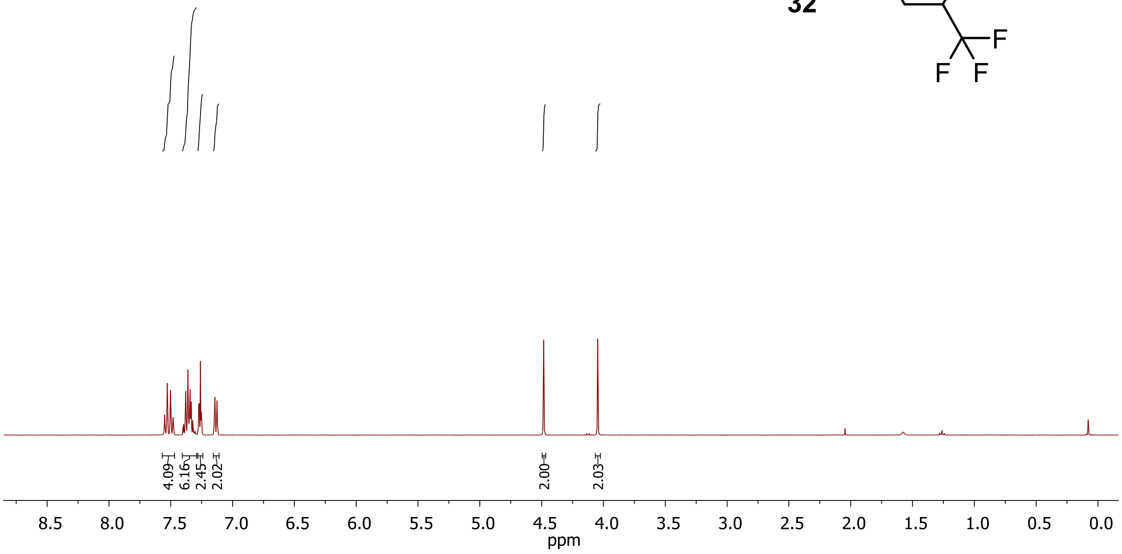

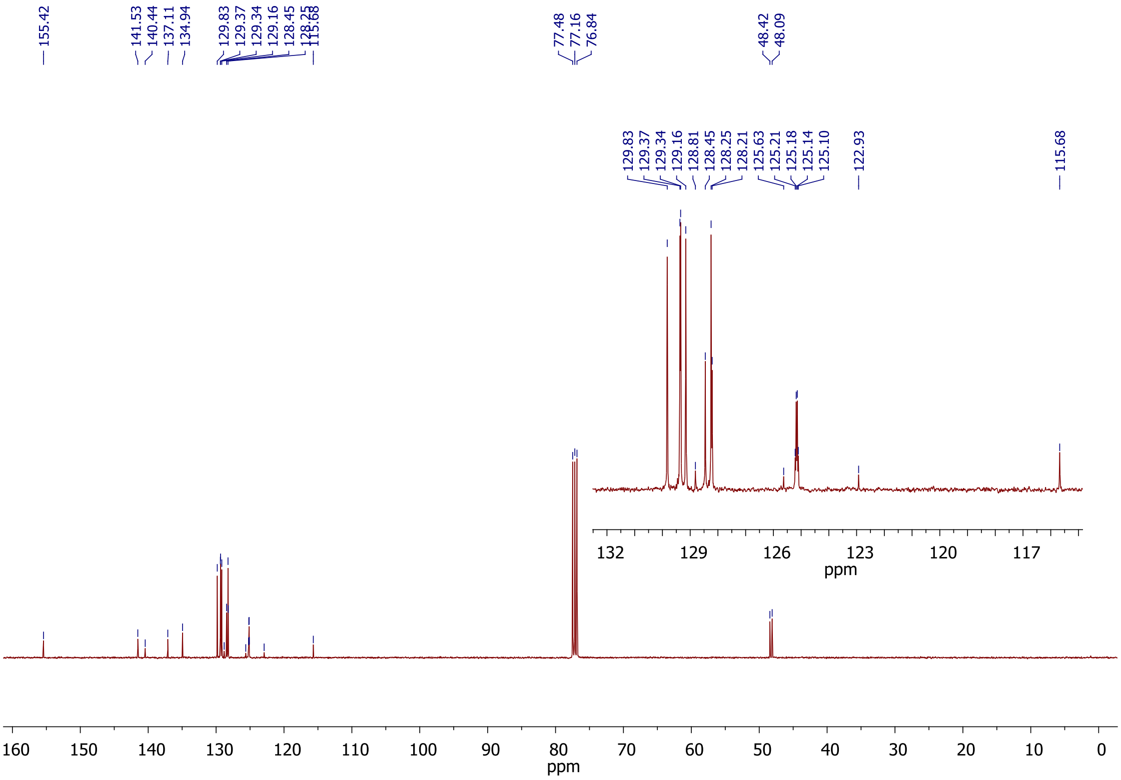
155.42
141.53
140.44
137.11
134.94
129.83
129.37
129.34
129.16
128.45
128.25
115.68
77.48
77.16
76.84
48.42
48.09
129.83
129.37
129.34
129.16
128.81
128.45
128.25
128.21
125.63
125.21
125.18
125.14
125.10
122.93
115.68
132
129
126
123
120
117
ppm
160
150
140
130
120
110
100
90
80
70
60
50
40
30
20
10
0
ppm

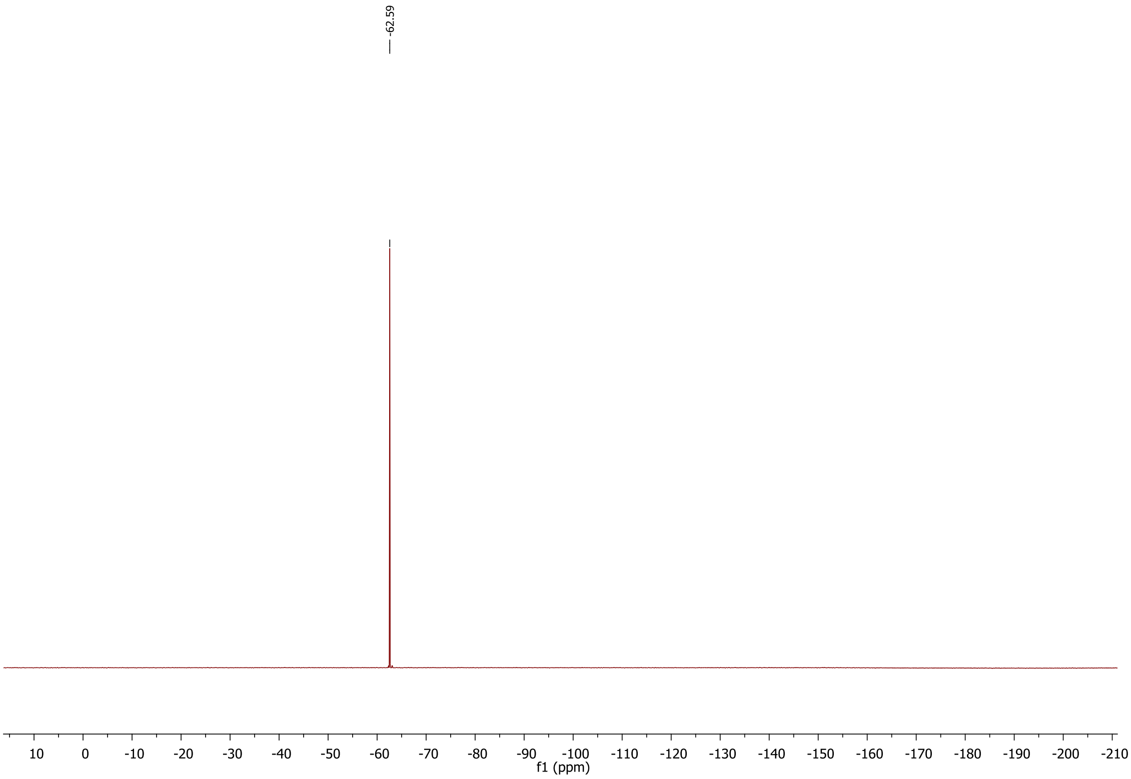
-62.59
10
0
-10
-20
-30
-40
-50
-60
-70
-80
-90
-100
-110
-120
-130
-140
-150
-160
-170
-180
-190
-200
-210
f1 (ppm)

(*Z*)-3-Benzyl-5-[(4-methoxyphenyl)phenylmethylene]oxazolidin-2-one (33)

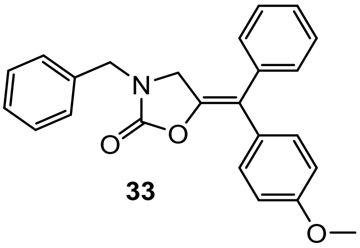

33

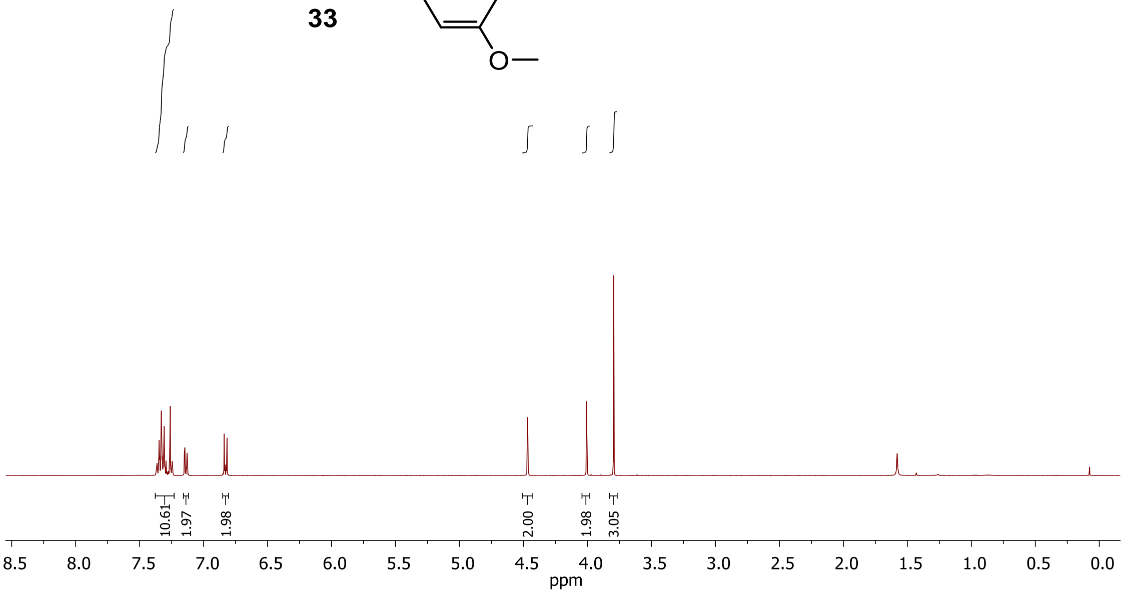

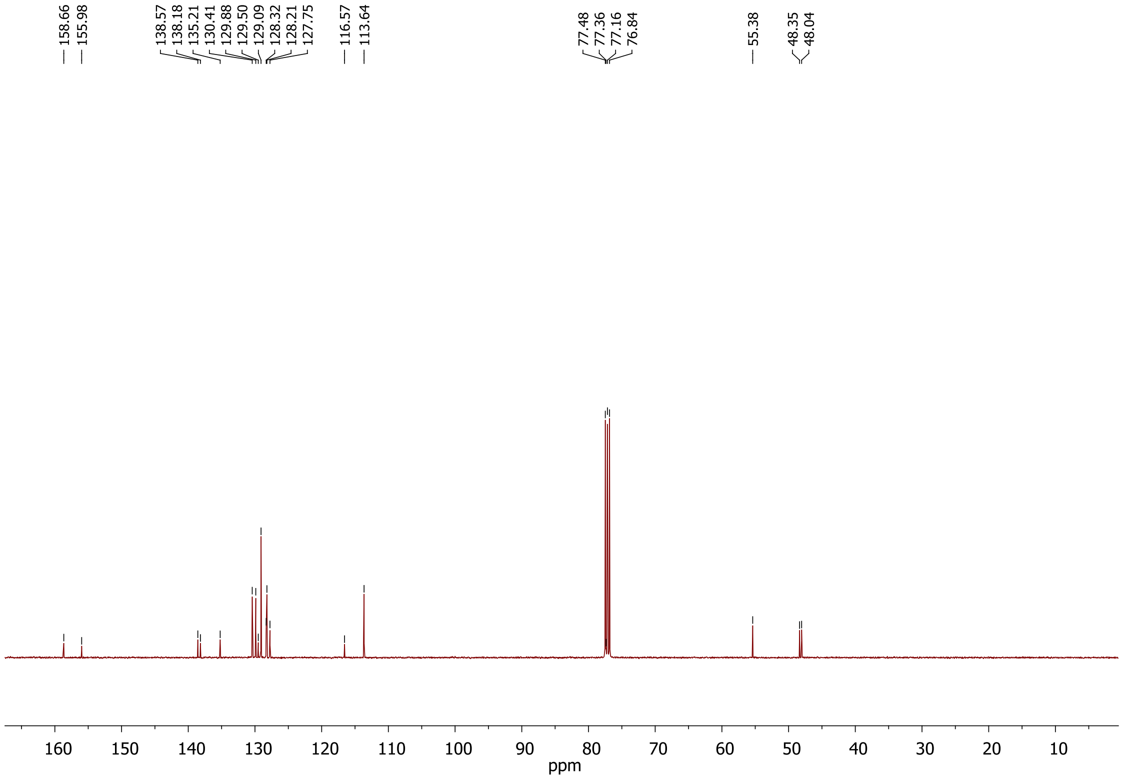

3-Benzyl-5-[bis(4-(trifluoromethyl)phenyl)methylene]oxazolidin-2-one (34)

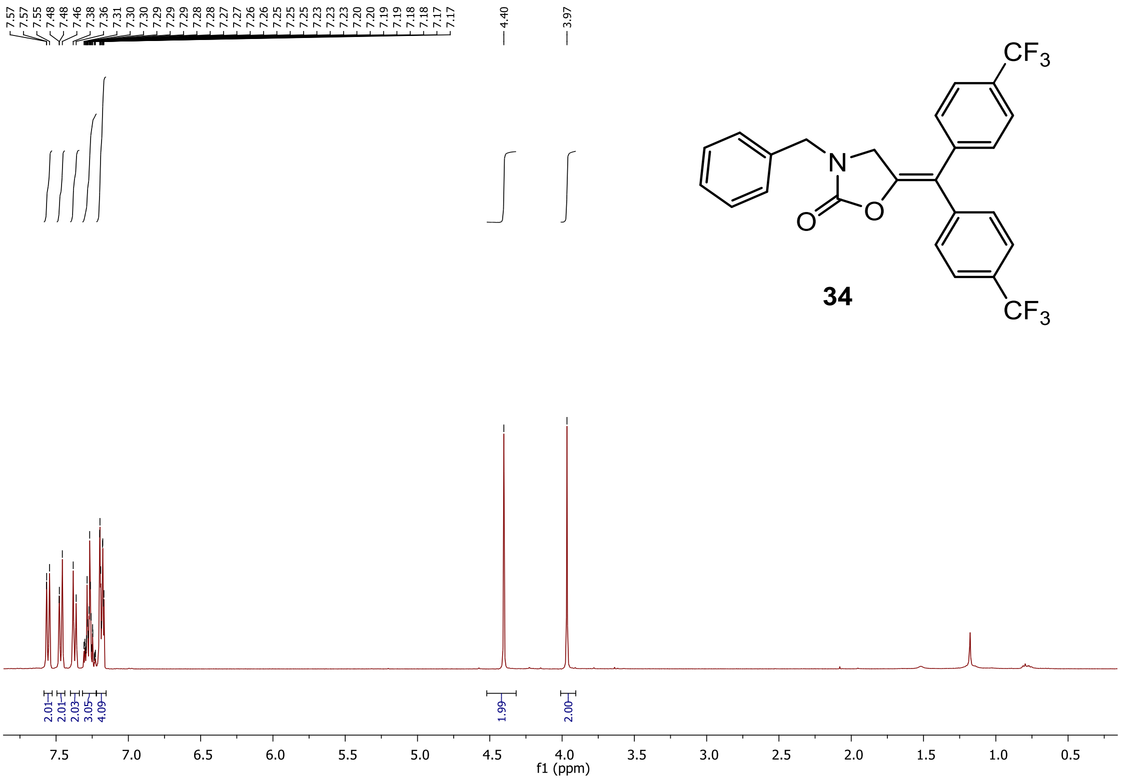

34

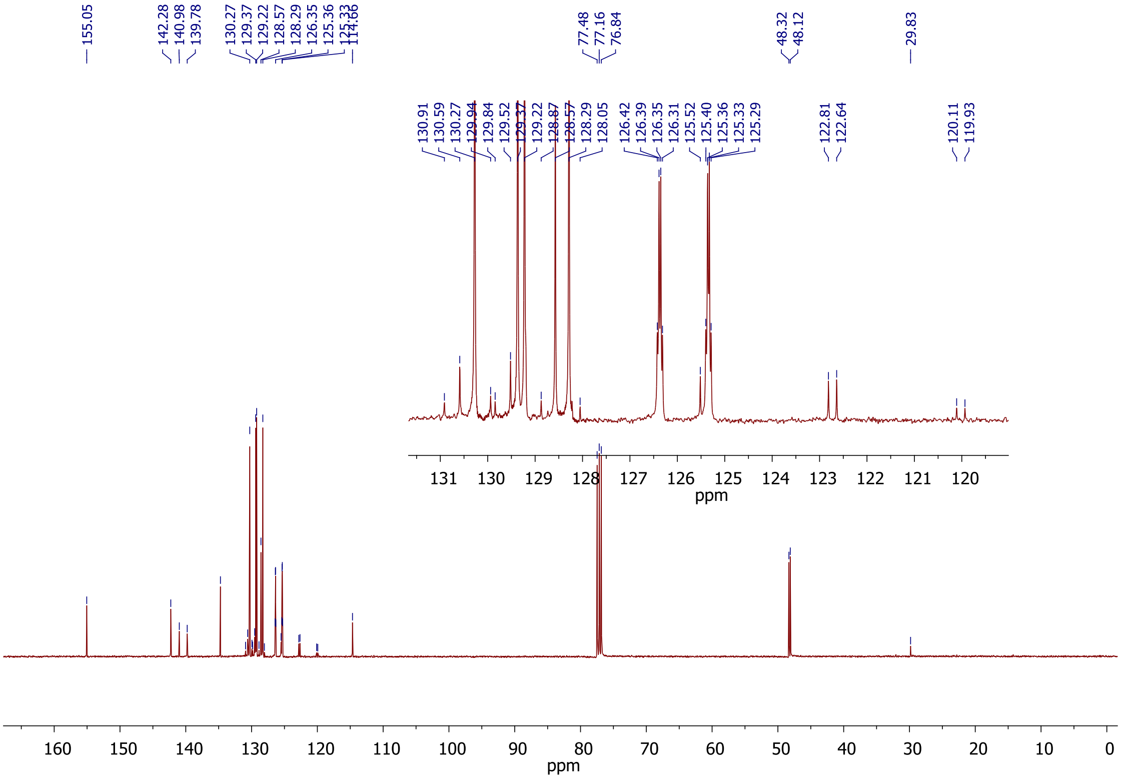

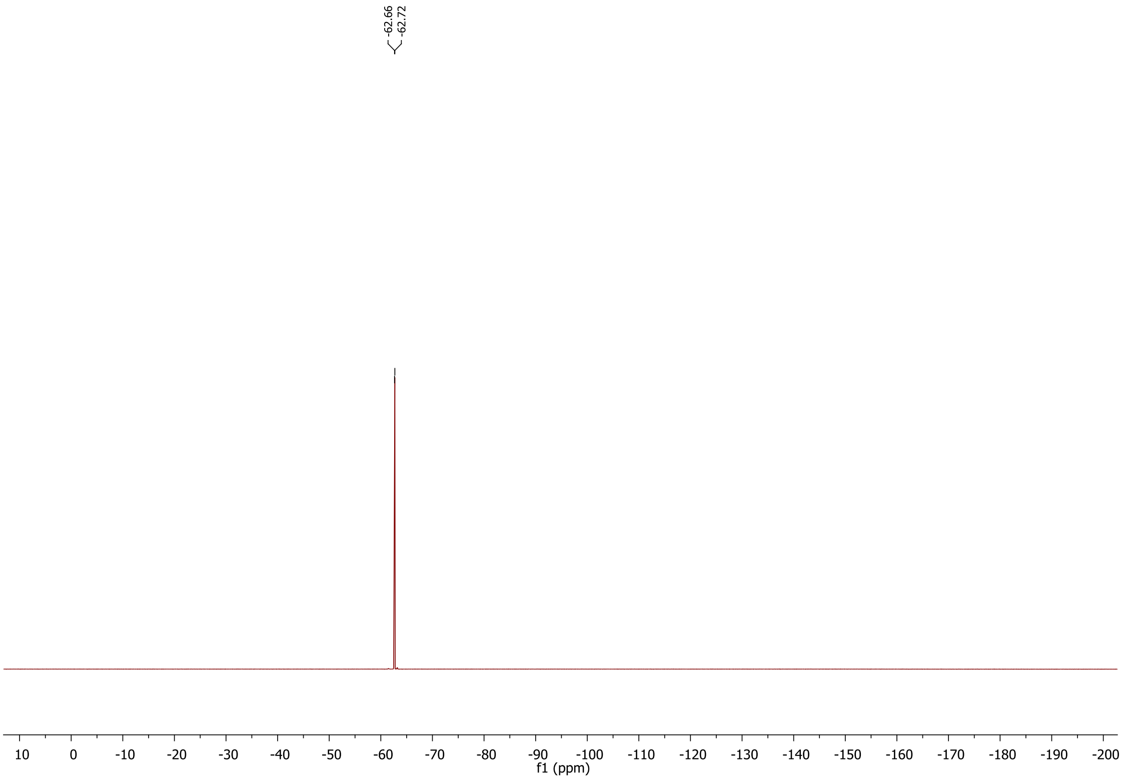
-62.66
-62.72
10
0
-10
-20
-30
-40
-50
-60
-70
-80
-90
-100
-110
-120
-130
-140
-150
-160
-170
-180
-190
-200
f1 (ppm)

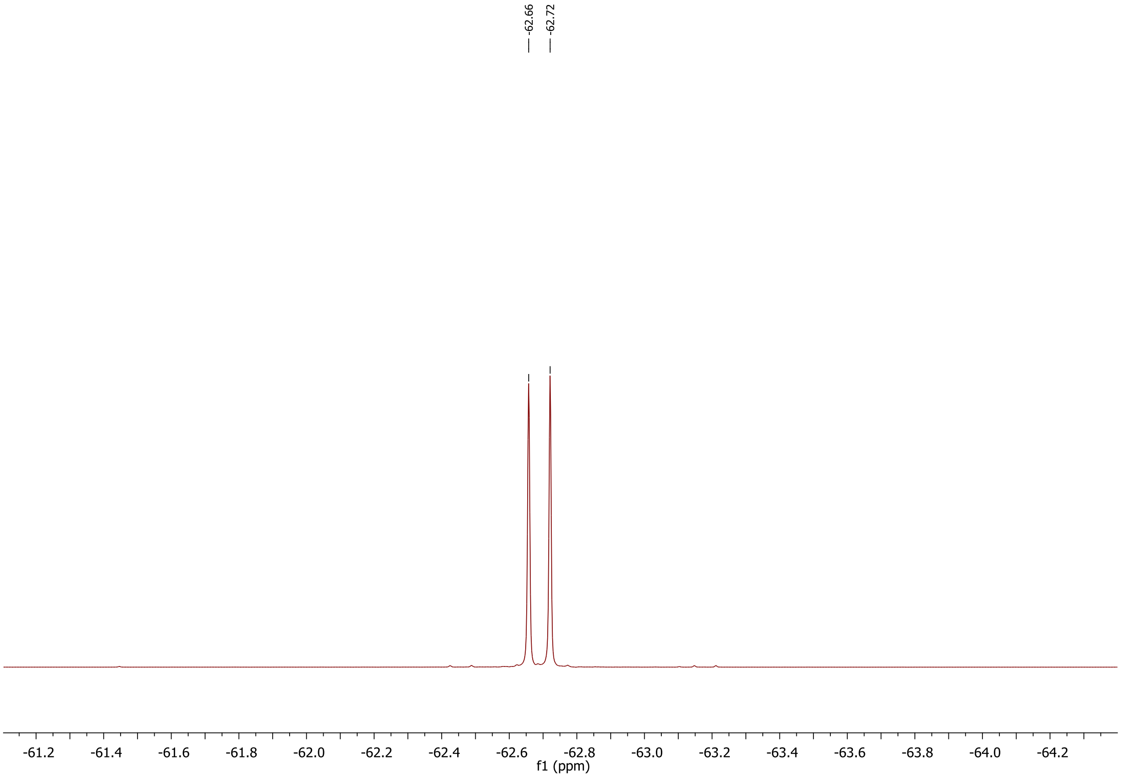
-62.66
-62.72
-61.2
-61.4
-61.6
-61.8
-62.0
-62.2
-62.4
-62.6
-62.8
-63.0
-63.2
-63.4
-63.6
-63.8
-64.0
-64.2
f1 (ppm)

(*Z*)-3-Benzyl-5-benzylideneoxazolidin-2-one (*Z*-7a)

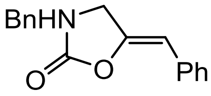

Z-7a

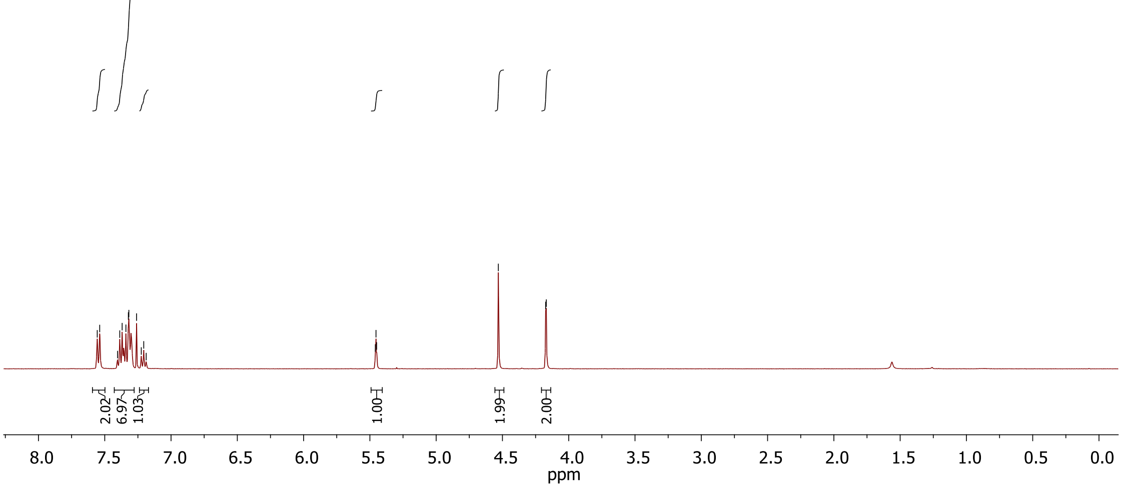

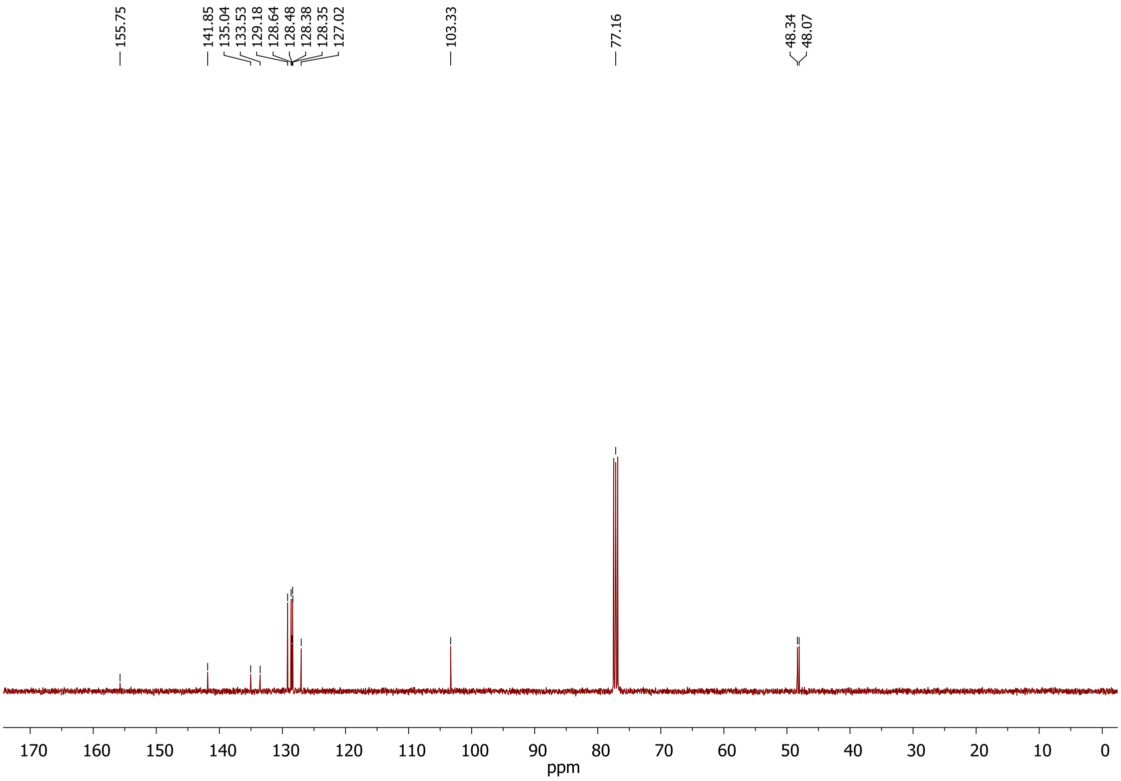

3-Benzyl-5-methyleneoxazolidin-2-one (S1)

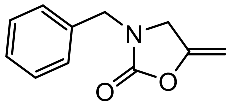

S1

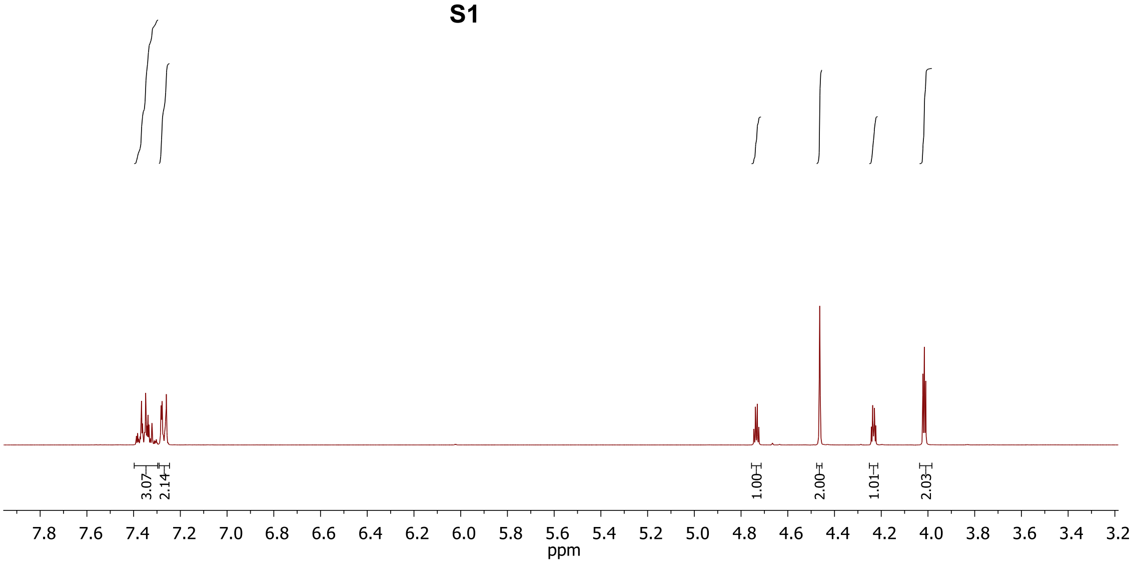

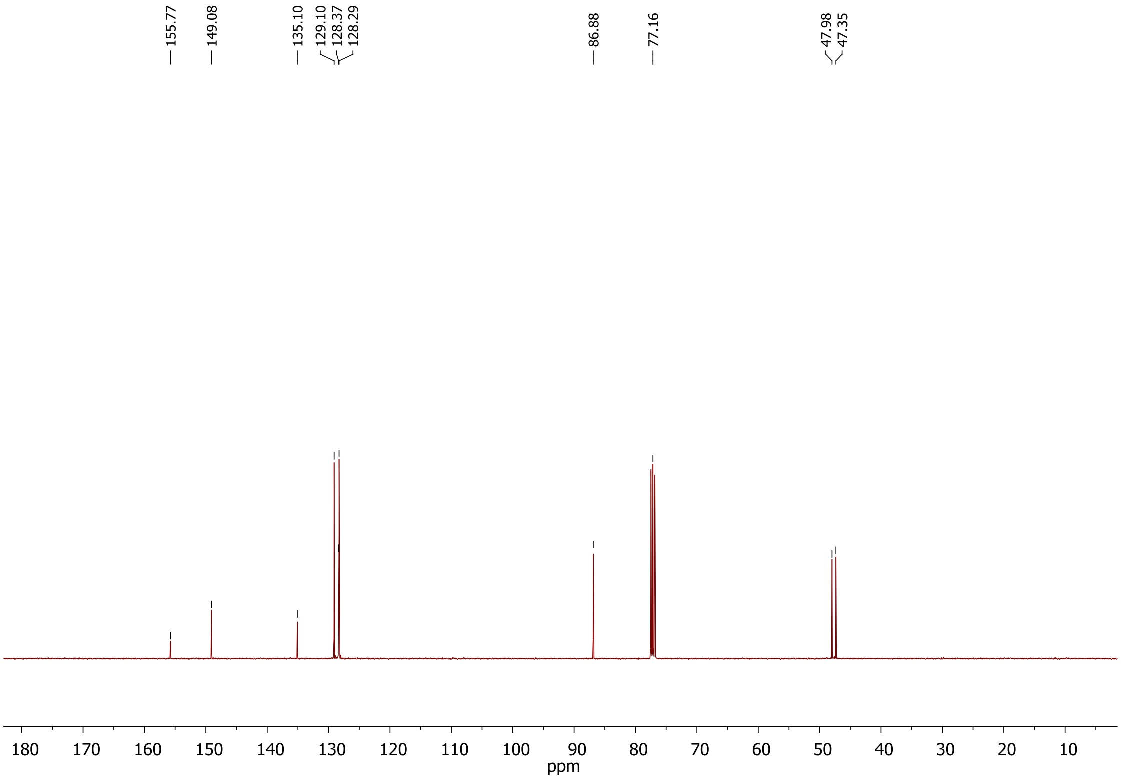

7. X-Ray Structures

7.1 Methyl (*E*)-4-[1-(3-benzyl-2-oxooxazolidin-5-ylidene)ethyl]benzoate (3c)

These crystallographic data for this compound can be obtained free of charge from The Cambridge Crystallographic Data Centre via www.ccdc.cam.ac.uk/data_request/cif.

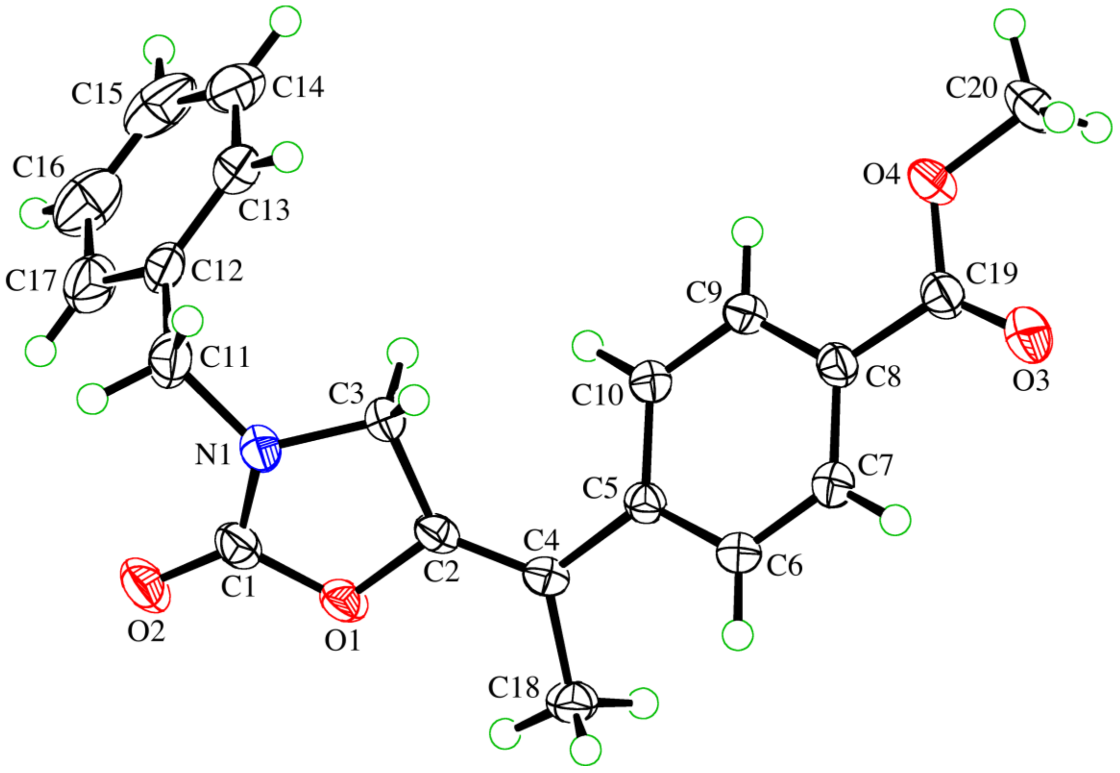

Table 1. Crystal data and structure refinement for oxazolidinone 3c (CCDC 1439789)

Crystallised from	CH_2Cl_2
Empirical formula	$C_{20}H_{19}NO_4$
Formula weight [g mol^{-1}]	337.37
Crystal colour, habit	colourless, prism
Crystal dimensions [mm]	0.13 × 0.20 × 0.27
Temperature [K]	160(1)
Crystal system	triclinic
Space group	$P\bar{1}$ (#2)
Z	8
Reflections for cell determination	31641
2θ range for cell determination [°]	4–149
Unit cell parameters *a* [Å]	12.43088(18)
b [Å]	15.8294(2)

c [Å]	18.0723(3)
α [°]	92.4918(12)
β [°]	101.8823(12)
γ [°]	100.5457(12)
V [Å^3]	3408.98(9)
$F(000)$	1424
D_x [g cm^{-3}]	1.315
μ(Cu $K\alpha$) [mm^{-1}]	0.752
Scan type	ω
$2\theta_{(max)}$ [°]	148.6
Transmission factors (min; max)	0.009; 1.000
Total reflections measured	64083
Symmetry independent reflections	13734
R_{int}	0.022
Reflections with $I > 2\sigma(I)$	12322
Reflections used in refinement	13734
Parameters refined	910
Final $R(F)$ [$I > 2\sigma(I)$ reflections]	0.0365
$wR(F^2)$ (all data)	0.0993
Weights:	$w = [\sigma^2(F_o^2) + (0.0490P)^2 + 0.8725P]^{-1}$ where $P = (F_o^2 + 2F_c^2)/3$
Goodness of fit	1.025
Secondary extinction coefficient	0.00065(6)
Final Δ_{max}/σ	0.001
$\Delta\rho$ (max; min) [e Å^{-3}]	0.25; -0.21
$\sigma(d_{(C-C)})$ [Å]	0.0014 – 0.003

7.2 (*E*)-3-Benzyl-5-benzylideneoxazolidin-2-one (7a)

These crystallographic data for this compound can be obtained free of charge from The Cambridge Crystallographic Data Centre via www.ccdc.cam.ac.uk/data_request/cif.

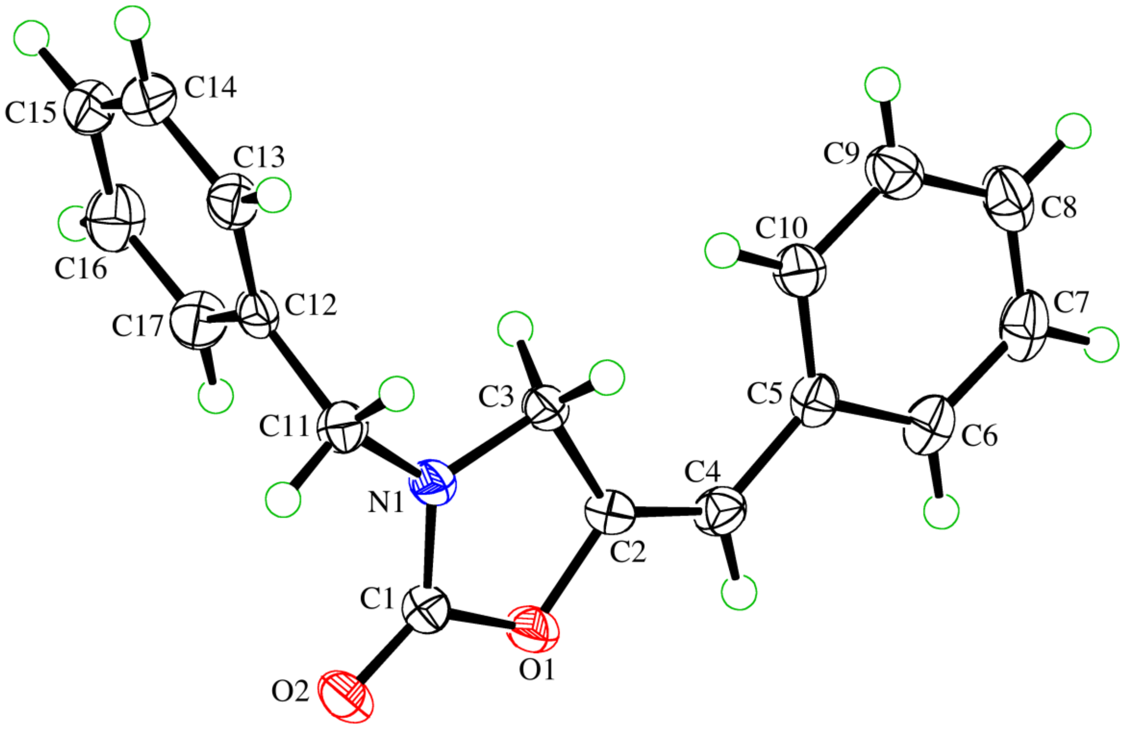

Table 1. Crystal data and structure refinement for oxazolidinone 7a (CCDC 1439720)

Crystallised from	CH_2Cl_2
Empirical formula	$C_{17.5}H_{16}ClNO_2$
Formula weight [g mol^{-1}]	307.78
Crystal colour, habit	colourless, prism
Crystal dimensions [mm]	0.16 × 0.30 × 0.35
Temperature [K]	160(1)
Crystal system	triclinic
Space group	$P\bar{1}$ (#2)
Z	4
Reflections for cell determination	15527
2θ range for cell determination [°]	3–149
Unit cell parameters *a* [Å]	5.98282(12)
b [Å]	11.5033(3)
c [Å]	22.3942(4)
α [°]	83.2783(17)

β [°]	89.5639(15)
γ [°]	85.3431(17)
V [Å^3]	1525.57(6)
$F(000)$	644
D_x [g cm^{-3}]	1.340
μ(Cu $K\alpha$) [mm^{-1}]	2.257
Scan type	ω
$2\theta_{(max)}$ [°]	148.6
Transmission factors (min; max)	0.304; 1.000
Total reflections measured	28793
Symmetry independent reflections	6131
R_{int}	0.020
Reflections with $I > 2\sigma(I)$	5742
Reflections used in refinement	6131
Parameters refined	361
Final $R(F)$ [$I > 2\sigma(I)$ reflections]	0.0433
$wR(F^2)$ (all data)	0.1183
Weights:	$w = [\sigma^2(F_o^2) + (0.0551P)^2 + 0.7012P]^{-1}$ where $P = (F_o^2 + 2F_c^2)/3$
Goodness of fit	1.060
Final Δ_{max}/σ	0.000
$\Delta\rho$ (max; min) [e Å^{-3}]	0.23; -0.33
$\sigma(d_{(C-C)})$ [Å]	0.0017 – 0.002

7.3 (*E*)-5-benzylideneoxazolidin-2-one (13a)

These crystallographic data for this compound can be obtained free of charge from The Cambridge Crystallographic Data Centre via www.ccdc.cam.ac.uk/data_request/cif.

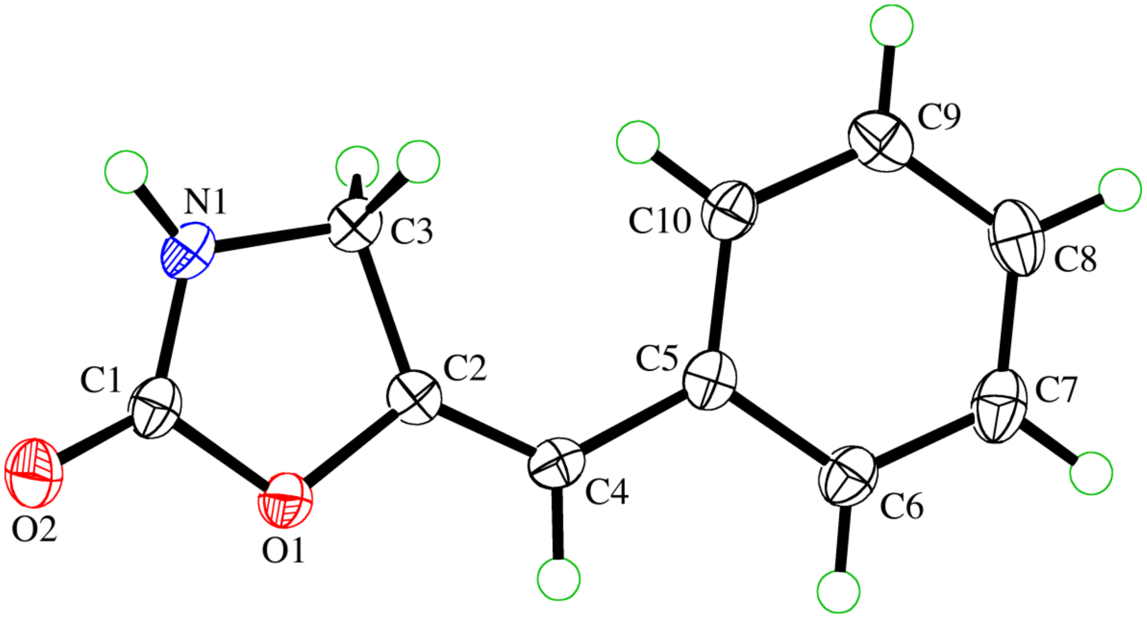

Table 1. Crystal data and structure refinement for oxazolidinone 13a (CCDC 1439788)

Crystallised from		CH_2Cl_2
Empirical formula		$C_{10}H_9NO_2$
Formula weight [g mol^{-1}]		175.19
Crystal colour, habit		colourless, prism
Crystal dimensions [mm]		0.13 × 0.24 × 0.34
Temperature [K]		160(1)
Crystal system		monoclinic
Space group		$P2_1/n$ (#14)
Z		4
Reflections for cell determination		4412
2θ range for cell determination [°]		7–56
Unit cell parameters	*a* [Å]	7.4980(2)
	b [Å]	5.69208(16)
	c [Å]	19.4188(5)
	α [°]	90
	β [°]	90.668(3)
	γ [°]	90

V [Å^3]	828.73(4)
$F(000)$	368
D_x [g cm^{-3}]	1.404
μ(Mo $K\alpha$) [mm^{-1}]	0.0989
Scan type	ω
$2\theta_{(max)}$ [°]	56.6
Transmission factors (min; max)	0.748; 1.000
Total reflections measured	8396
Symmetry independent reflections	1878
R_{int}	0.022
Reflections with $I > 2\sigma(I)$	1587
Reflections used in refinement	1878
Parameters refined	123
Final $R(F)$ [$I > 2\sigma(I)$ reflections]	0.0344
$wR(F^2)$ (all data)	0.0830
Weights:	$w = [\sigma^2(F_o^2) + (0.0301P)^2 + 0.3124P]^{-1}$ where $P = (F_o^2 + 2F_c^2)/3$
Goodness of fit	1.050
Secondary extinction coefficient	0.009(2)
Final Δ_{max}/σ	0.000
$\Delta\rho$ (max; min) [e Å^{-3}]	0.23; -0.19
$\sigma(d_{(C-C)})$ [Å]	0.0015 – 0.0018

8. References

[1] W. L. F. Armareg, D. D. Perrin, *Purification of Laboratory Chemicals*, Butterworth-Heinemann, Oxford, **1996**.

[2] aR. W. Foster, C. J. Tame, H. C. Hailes, T. D. Sheppard, *Adv. Synth. Catal.* **2013**, *355*, 2353-2360; bY. Hirata, T. Yukawa, N. Kashihara, Y. Nakao, T. Hiyama, *J. Am. Chem. Soc.* **2009**, *131*, 10964-10973.

[3] S. Hase, Y. Kayaki, T. Ikariya, *ACS Catalysis* **2015**, *5*, 5135-5140.

[4] S. Yoshida, K. Fukui, S. Kikuchi, T. Yamada, *Chem. Lett.* **2009**, *38*, 786-787.

[5] C. Molinaro, T. F. Jamison, *J. Am. Chem. Soc.* **2003**, *125*, 8076-8077.

[6] R. Parcell, C. Pollard, *J. Am. Chem. Soc.* **1950**, *72*, 3312-3313.

[7] S. Hase, Y. Kayaki, T. Ikariya, *Organometallics* **2013**, *32*, 5285-5288.

[8] T. Howard Black, in *Encyclopedia of Reagents for Organic Synthesis*, John Wiley & Sons, Ltd, **2001**.

[9] M. S. Baird, A. G. W. Baxter, A. Hoorfar, I. Jefferies, *J. Chem. Soc., Perkin Trans. 1.* **1991**, 2575-2581.

[10] J. Barluenga, F. Foubelo, F. J. Fananas, M. Yus, *J. Chem. Soc., Perkin Trans. 1.* **1989**, 553-557.

[11] M. Yoshida, T. Mizuguchi, K. Shishido, *Chem. Eur. J.* **2012**, *18*, 15578-15581.

[12] H. v. Wachenfeldt, F. Paulsen, A. Sundin, D. Strand, *Eur. J. Org. Chem.* **2013**, *2013*, 4578-4585.

[13] H.-L. Cui, F. Tanaka, *Org. Biomol. Chem.* **2014**, *12*, 5822-5826.

[14] aJ. Liu, Y. Liu, *Org. Lett.* **2012**, *14*, 4742-4745; bK. Sakaguchi, M. Ayabe, Y. Watanabe, T. Okada, K. Kawamura, T. Shiada, Y. Ohfune, *Org. Lett.* **2008**, *10*, 5449-5452.

[15] aA. K. Chakraborti, S. V. Chankeshwara, *J. Org. Chem.* **2009**, *74*, 1367-1370; bY. L. Jin, S. Kim, Y. S. Kim, S.-A. Kim, H. S. Kim, *Tetrahedron Lett.* **2008**, *49*, 6835-6837.

[16] J. Chen, A. J. McNeil, *J. Am. Chem. Soc.* **2008**, *130*, 16496-16497.

[17] K. M. Brummond, T. O. Painter, D. A. Probst, B. Mitasev, *Org. Lett.* **2007**, *9*, 347-349.
